# BSA-seq integrated with transcriptomics and metabolomics revealing the candidate genes associated with safflower colors and flavonoid glycosides biosynthesis

**DOI:** 10.1093/hr/uhag068

**Published:** 2026-03-04

**Authors:** Haotian Wang, Beixuan He, Shuyi Qi, Yue Gao, Xin Dong, Meili Guo

**Affiliations:** Department of Pharmacognosy, College of Pharmacy, Naval Medical University, Shanghai 200433, China; School of Pharmacy, Bengbu Medical University, Bengbu 233030, China; Department of Pharmacognosy, College of Pharmacy, Naval Medical University, Shanghai 200433, China; Department of Pharmacognosy, College of Pharmacy, Naval Medical University, Shanghai 200433, China; Department of Pharmacognosy, College of Pharmacy, Naval Medical University, Shanghai 200433, China; School of Medicine, Shanghai University, Shanghai 200444, China; Department of Pharmacognosy, College of Pharmacy, Naval Medical University, Shanghai 200433, China

## Abstract

Safflower is featured with time-honored medical and economic values and developing into diverse phenotypic and genetic variations. In this study, to explore the critical genes associated with color phenotypes and flavonoid derivatives biosynthesis of safflower, BSA-seq, conflated with transcriptomic and metabolic methods were performed in two extreme colors (yellow and white) in the population of ‘ZHH0119’ and ‘XHH007.’ After crossing two parent plants reciprocally, the F_4_ generation of two accessions were used to construct near-isogenic gene pools for the two extreme traits of yellow and white safflower. BSA-seq results located five QTLs regions on chromosomes 2, 8, 9, 10, and 12 including 6 *CtPALs*, 3 *CtC4Hs*, 2 *Ct4CLs*, 1 *CtCHS*, 32 *CtUGTs,* and 70 *CtCYPs*, which tied to the yellow color phenotype of safflower. Through transcriptome analysis of two accessions and at different flowering stages, 1 *CtPAL*, 5 *CtC4Hs*, 4 *CtCHSs*, 3 *CtCHIs*, 3 *CtFLSs*, 48 *CtUGTs*, 51 *CtCYPs,* and 75 transcription factors were revealed as significantly upregulated in the yellow accession compared to the white. Integrated analysis identified eight *CtUGTs* (*CtUGT50-57*) that exhibited significant positive correlations with chalcone glycosides of yellow safflower. Based on functional characterization, *CtUGT52* was found to boost Hydroxysafflor yellow A (HSYA) content in yellow safflower which possessing substrate promiscuity (chalones, flavonols, and flavonoids) and catalytic promiscuity (flavonols and flavonoids), revealing its vital role in the HSYA biosynthesis through transgenic overexpression. Combining catalytic mechanism verification of *Ct*UGT52 towards phloretin, kaempferol, and luteolin, our study to some extent, elucidated the modification function of *Ct*UGTs for flavonoid aglycones in the flavonoid biosynthesis pathway of safflower.

## Introduction

Safflower (*Carthamus tinctorius* L.), as a renowned traditional Chinese medicine also oilseed plant, has been cultivated around the world. With its high pharmaceutical value for promoting blood circulation and removing blood stasis, it has been widely used for more than two thousand years in China [1–3]. Flavonoids and polyunsaturated fatty acids are the main bioactive chemical constituents of safflower [4], thereinto, Hydroxysafflor yellow A (HSYA), a quinochalcone C-glycoside, has been marked as the characterized component which show versatile effects on cardiovascular and cerebrovascular system [5] as well as osteoprotective and anti-diabetic effects in recent studies [6–8]. Meanwhile, safflower has developed into thousands of accessions with various traits as a result of genetic and environmental diversities, providing valuable resources for breeding superior accessions with high medicinal and economic value [9].

Corolla color is a prominent phenotype and agronomic trait of safflower and color transition from yellow to red always happens along with its growing stages. This dynamic change reflects the versatility of metabolites molecular mechanism and accumulation, especially flavonoids components. Although the phenotypic traits of safflower are also affected by epigenetic regulation, environmental factors, and other elements, the genetic variations including structural genes, modification genes, and transcription factors are inferred to affect the biosynthesis of color-related components in safflower [4, 10]. Recent study has demonstrated that the modification enzymes such as *Ct*F6H (flavanone 6-hydroxylase), glycosyltransferase, and cytochrome P450 could the key factors responsible for their inability to produce HSYA [11]. While the intricate regulation of flavonoid biosynthesis underlying various colors, which involves coordinated gene network rather than single genetic determinant, remains to be comprehensively elucidated through integrated analysis of distinct safflower phenotypes.

Multi-omics studies could elucidate gene functions and reveal the regulatory networks underlying the synthesis of Chinese traditional medicine hallmark components [12–15]. In 2021, a high-quality reference genome at chromosome-scale of safflower was reported for the first time, combined with transcriptomic analysis, the study provided insights into the biosynthesis of flavonoid and linoleic acid [16]. Meanwhile, with the onward march of sequencing technology, more methods have been employed to provide a genome understanding of the molecular mechanism underlying the traits. Bulk Segregation Analysis (BSA) could map quantitative trait loci (QTL) rapidly and locate the candidate genes that control extreme phenotypes via constructing mixed pools and it has been increasingly employed in the studies of different species, including grain crops, economic crops, horticultural crops, trees, aquatic animals, etc., to precisely define the QTLs that coupled with the traits [17–19].

In our previous studies, two extreme phenotypic and genetically isolated safflower germplasm resources have been obtained: a yellow accession with high HSYA content (ZHH0119) and a white accession with HSYA-absent but high nicotiflorin content (XHH007). In the light of the very correlation between color and flavonoids composition of safflower, for the first time, BSA-seq was conducted to elucidate the genes associated with yellow color phenotype of safflower in this study. By comparing the transcriptomic and metabolomic data, the candidate genes which were believed leading to discrepancies flavonoid derivatives expression in different safflower, of which eight *CtUGTs* functions have been characterized. Overall, the identification of key glycosyltransferases, particularly *Ct*UGT52, represented critical steps in understanding the molecular mechanisms of flavonoid biosynthesis including HSYA in safflower. It has important implications for both safflower breeding and the biomanufacturing of medicinal compounds. The following is our first report of the study.

## Results

### Qualitative analysis of BSA-Seq data and single-nucleotide polymorphism detection

**Figure 1:**
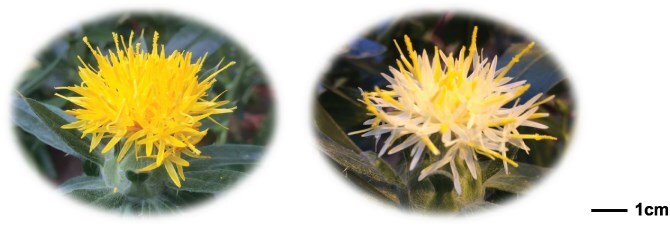
Morphological description of accessions ZHH0119 (left) and XHH007 (right).

To decipher the differences of safflower color and metabolite accumulation at genomic level, two accessions (as shown in Fig. 1) were collected for BSA-Seq.

**Figure 2:**
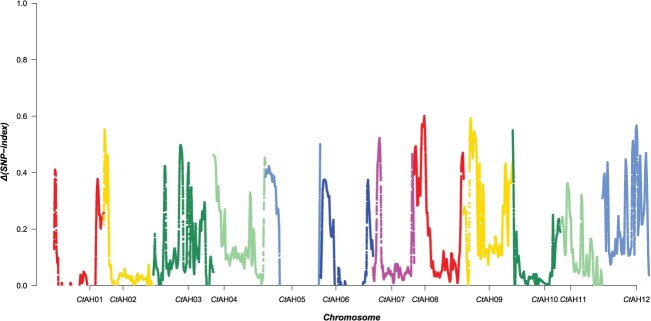
Δ(SNP-index) plot of the extreme pools.

A total of 377 097 227 base pairs aligned successfully to the reference genome (PRJNA642978) by BWA, while 4 039 125 base pairs did not align. The average coverage depth was 46.88104, with the proportions of bases covered at or above the specified depths being 0.983362 (1×), 0.975526 (5×), and 0.968039 (10×) in the reference genome (as shown in Table S1). Then, 2 202 977 (Yellow accession) and 1 373 482 (White accession) SNPs have been identified and filtrated from two constructed extreme pools. Δ(SNP-index) were calculated to identify QTL regions that associated with the yellow phenotype (as shown in Fig. 2). Five regions including 12.5–19.9 Mb of chromosome 8, 10.6–16.4 Mb of chromosome 9, 58.5–62.1 Mb of chromosome 12, 11.4–31.2 Mb of chromosome 2, and 0.002–0.083 Mb of chromosome 10 were most likely contains genes that being attribution of different phenotypes (as shown in Table 1). Gene annotation of SNP sites within the candidate regions revealed 1923 genes containing these SNPs. Based on alignment with the reference genome and gene annotation, genes associated with the biosynthesis of flavonoid compounds were identified including 6 *CtPALs*, 3 *CtC4Hs*, 2 *Ct4CLs*, 1 *CtCHS*, 32 *CtUGTs,* and 70 *CtCYPs*, as well as transcription regulatory factors encoding genes including 67 *CtbHLHs*, 248 *CtMYBs*, 101 *CtWRKYs*, and 50 *CtWD40s*.

**Table 1:** Top5 QTL regions.

CHROM	start	end	length	nSNPs	DeltaSNP
*Ct*AH02	1 144 455	3 129 284	1 984 829	1344	0.552024
*Ct*AH08	12 501 664	19 972 316	7 470 652	12 090	0.600301
*Ct*AH09	10 649 708	16 409 714	5 760 006	5854	0.592525
*Ct*AH10	28 665	830 148	801 483	1511	0.550139
*Ct*AH12	58 560 864	62 121 641	3 560 777	11 797	0.566725

### Transcriptome analysis of different flower color accessions and various growing stages in safflower

#### Overview of transcriptome sequencing analysis and annotation

Two safflower accessions, including one white and four different growing stages yellow safflower (as shown in Fig. S1), were sequenced by Illumina platform, and a total of 221.53Gb quality-filtered data were obtained. All samples GC contents varied from 46% to 48% with Q30 > 92.9%. The reads were aligned to reference genome showed mapping ratios of the samples ranged from 86.05% to 95.41%. Reference-based assembly with StringTie identified 77 240 transcripts, of which 45 331 were matched with intron chains and 31 909 were potentially novel isoforms. Based on the comparison with six databases (NR/Swiss-Prot/Pfam/EggNOG/GO/KEGG), all transcripts comprising novel transcripts were assigned putative annotations. Further screening was conducted for genes encoding enzymes involved in the flavonoid biosynthesis, resulting in the identification of 5 *CtPALs*, 25 *Ct4CLs*, 6 *CtCHSs*, 6 *CtCHIs*, 4 *CtFNSs*, 4 *CtDFRs*, 169 *CtUGTs,* and 343 *CtCYPs*.

Comparative analysis of significantly differentially expressed genes (DEGs) among various safflower phenotypes revealed 117 up-regulated and 46 down-regulated DEGs when compared Y_III with W_III group. When it came to the DEGs among various growing stages, there were 1933 up-regulated and 1114 down-regulated DEGs when Y_IV compared to Y_I group, while no up-regulated and 1 down-regulated DEG when Y_III compared to Y_II group (as shown in Fig. S2).

The largest number of significantly DEGs with catalytic activity was found between different color phenotypes when classified according to molecular function (as shown in Fig. S3a and b). The KEGG metabolic pathway classification results showed the largest number of DEGs assigned to metabolic processes, with a significant proportion involved in carbohydrate and lipid metabolism. There were 38 DEGs annotated with glycosyltransferase activities within the different flower color accession, with an enrichment factor of 0.208 (as shown in Fig. S3c and d). KEGG enrichment analysis revealed a greater enrichment in genes involved in the biosynthesis of unsaturated fatty acids and monoterpenoids, while the highest number of DEGs were associated with the phenylpropanoid metabolism pathway (a total of 39) and 14 were related to flavonoid biosynthesis. The significant enrichment in the phenylpropanoid and flavonoid metabolic pathways of DEGs suggested that there are some DEGs could affect the metabolic flux of phenylpropanoid and the propensity of flavonoids, leading to the differential flavonoid accumulation.

#### Weighted correlation network analysis of DEGs

**Figure 3:**
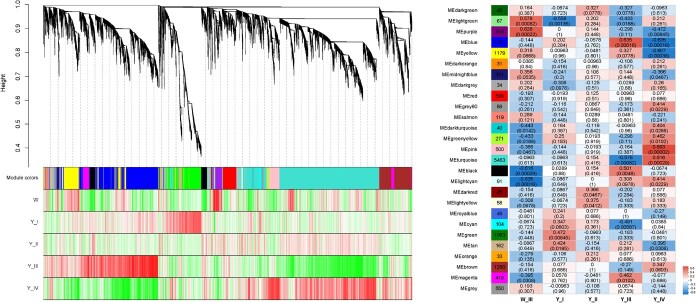
WGCNA of DEGs and heatmap of correlation between different groups and modules.

As shown in Fig. 3, weighted correlation network analysis (WGCNA) analysis revealed that the highest correlation of green module with Y_I, lightyellow module with Y_II, blue module with Y_III, pink module with Y_IV, and purple module with W_III respectively. The genes in blue module that showed higher correlation with Y_III were enriched in plant signaling hormone transduction and α-linolenic acid metabolism, including 21 *CtUGTs*, 40 *CtCYPs*, and 102 transcription factor-encoding genes. In contrast, the purple and lightgreen modules, which showed higher correlation with W_III, predominantly enriched in pathways of monoterpene metabolism and valine degradation including only 2 *CtUGTs*, 2 *CtCYPs,* and 16 transcription factor-encoding genes.

### Nontargeted and targeted metabolomic comparison between yellow and white safflower

#### Overview of nontargeted metabolomic analysis

Principal Component Analysis (PCA) in both ionization modes showed significant separation differences between yellow and white safflowers (as depicted in Fig. S4a), while Y_II to Y_IV stages clustered from Y_I suggesting that the dynamic process of different stages in yellow accession. Venn analysis indicated that the white safflower contained 27 unique metabolites compared to the yellow accession. Disparities in metabolite profiles were observed among different growing stages of the yellow accession, with unique metabolites 3, 2, and 7 for stages Y_I, Y_III, and Y_IV, respectively. No unique metabolites were identified for stage Y_II compared to the other three stages. A total of 2305 shared metabolites were found between the two distinct accessions (as shown in Fig. S4b).

### Differentially expressed metabolites of nontargeted metabolomic analysis

Comparative analysis of significantly differentially expressed metabolites (DEMs) among various safflower phenotypes showed 248 up-regulated and 240 down-regulated DEMs when compared Y_I with W_III group, 203 up-regulated and 113 down-regulated DEMs when compared Y_II with W_III group, 346 up-regulated and 190 down-regulated DEMs when compared Y_III with W_III group, and 334 up-regulated and 185 down-regulated DEMs when compared Y_ IV with W_III group. When it came to the DEMs among various growing stages, there were 291 up-regulated and 170 down-regulated DEMs when Y_II compared to Y_I group, 410 up-regulated and 124 down-regulated DEMs when Y_III compared to Y_I group, 448 up-regulated and 132 down-regulated DEMs when Y_IV compared to Y_I group, 113 up-regulated and 68 down-regulated DEMs when Y_III compared to Y_II group, 126 up-regulated and 56 down-regulated DEMs when Y_IV compared to Y_II group, and 26 up-regulated and 23 down-regulated DEMs when Y_IV compared to Y_III group (as shown in Fig. S5).

**Figure 4:**
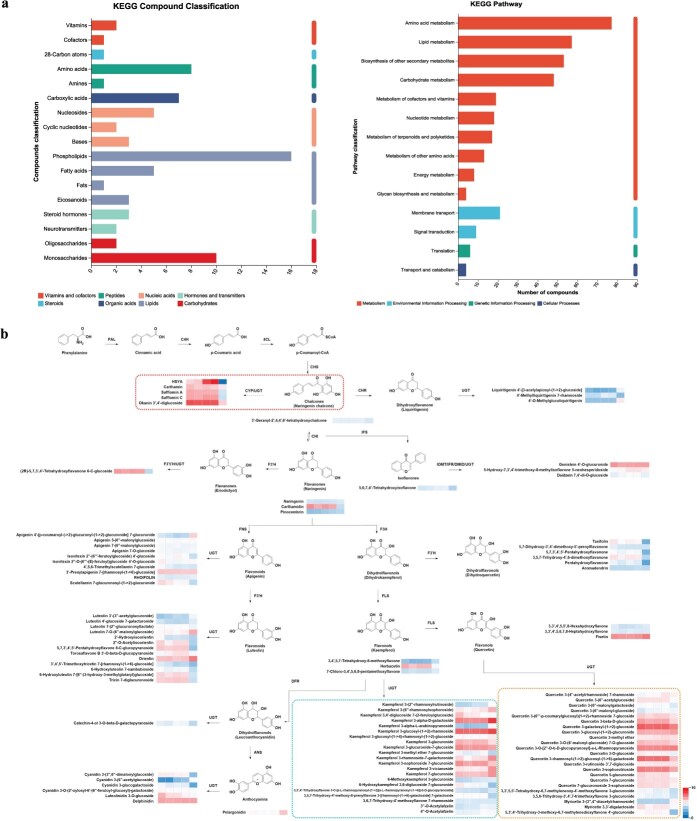
(a) KEGG analysis of DEMs. (b) Heatmap of flavonoids accumulation levels in different safflower accessions (from left to right: Y_I, Y_II, Y_III, Y_IV, and W_III).

As shown in Fig. 4a, KEGG compound classification and functional pathway statistics indicated that phospholipids and monosaccharides account for the largest proportion of DEMs. And these DEMs were primarily involved in the biosynthetic of amino acids, lipids, and other secondary metabolites. And metabolic profiles identified 130 flavonoid derivatives in two safflower accessions while 41 of the them showed significant expression difference.

Based on established biosynthetic pathways of flavonoids [20–22] and to better understand the potential regulatory mechanisms, the synthesis processes and accumulation patterns of flavonoid derivatives between two accessions have been mapped (Fig. 4b). Notably, the consistent elevation of chalcone glycosides (HSYA/ carthamin/ safflomin A and C) in the yellow accession across developmental stages compared to the white confirmed the presence of hub proteins (e.g., *Ct*UGT/*Ct*CYP), which trigger selective structural modifications towards to the chalcones in the yellow accession.

As a critical intermediate, naringenin could be responsible for the conversion of flavonoid derivatives [23] while metabolomic profiling showed no significant difference of naringenin between two accessions. And carthamidin exhibited significantly higher enrichment in the yellow accession. Particularly, the derivatives of kaempferol were more abundant prevalently in the white accession and the quercetin derivatives expression levels were higher in the yellow accession in contrast, which further suggested that there were certain *Ct*UGTs and *Ct*CYPs exhibit different substrate selectivity in different safflower, leading to discrepancies flavonoid derivatives expression.

#### Targeted metabolomic analysis

The accumulation levels of 13 target metabolites including phenylalanine/HSYA/nicotiflorin/naringenin/eriodictyol/apigenin/kaempferol/luteolin/astragalin/scutellarin/quercetin/isoquercitrin and rutin were profiled in two different safflower color accessions and various growing stages (as shown in Fig. S6). In yellow safflower, HSYA tended to increase from Y_I to Y_IV stage and there was an obvious climb during Y_III to Y_IV, while no HSYA had been detected in white safflower. The relative content of nicotiflorin in white safflower was twenty times much more than yellow safflower. The content variation of its aglycone kaempferol, as well as astragalin (kaempferol-3-*O*-glucoside) and scutellarin, were consistent with those of nicotiflorin. The accumulation differences of phenylalanine and other common flavonoids were less pronounced.

### Characterization of the genes related to flavonoid glycosides biosynthesis

To identify candidate genes associated with flavonoid glycoside biosynthesis, Pearson correlation analysis was performed between differentially expressed *CtUGTs* in yellow safflower and quantitative metabolite data of flavonoid glycosides including HSYA, with genes exhibiting a high positive correlation coefficient (*r* > 0.9) preliminarily screened (as shown in Fig. S7); additionally, based on the target genomic regions identified by BSA-seq, genes encoding glycosyltransferases were further selected as target genes for subsequent investigation. In all, sixteen *CtUGTs* were identified as the potential candidate genes that dictate the biosynthesis of glycosides in yellow safflower and ultimately contributing to the formation of flower color. The expression profiles of the 16 screened *CtUGTs* were depicted in Fig. S8. The full-length sequences of eight *CtUGTs* (*CtUGT50*-*57*) have been cloned and their basic information and sequences were listed in Tables S2 and S3.

Phylogenetic analysis (shown in Fig. S9) revealed that *CtUGT50* and *CtUGT56* belonged to the UGT87A subfamily, *CtUGT51*, *CtUGT53,* and *CtUGT54* belonged to the UGT71 family, *CtUGT52* belonged to the UGT74B subfamily, *CtUGT55* belonged to the UGT708 family and *CtUGT57* belonged to the UGT76 family. Meanwhile, most of them exhibited higher homology (75.68%–87.26%) with *UGTs* from *Cynara cardunculus*, which was consistent with the safflower genome evolutionary analysis.

#### Functional characterization of *Ct*UGTs *In vitro*

After expressed in *E. coli* the recombinant *Ct*UGTs were purified and confirmed by SDS-PAGE analysis (as shown in Fig. S10). Enzymatic assays were conducted with UDP-glucose serving as the sugar donor and seven different flavonoids acting as sugar acceptors. The enzymatic reactions products were characterized by HPLC and LC–MS/MS (as shown in Fig. S11).

**Figure 5:**
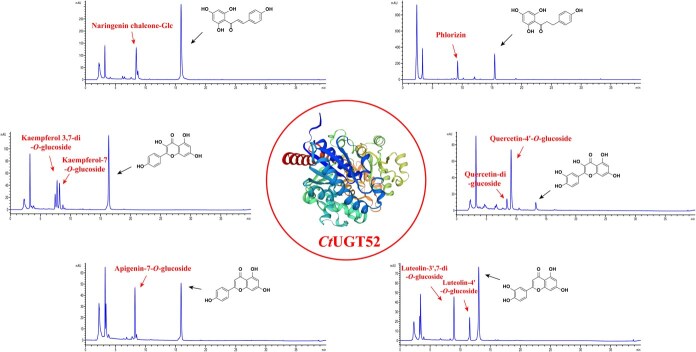
Catalytic promiscuity for glycosylation of *Ct*UGT52 *in vitro*.

It was intriguing that *Ct*UGT52 showed significant substrate promiscuity and catalytic promiscuity. As shown in Fig. 5, the prokaryotic expressed *Ct*UGT52 could catalyze the formation of monoglucosides from chalcone compounds such as naringenin chalcone and phloretin, as well as synthesizing monoglucosides and diglucosides from flavonols (kaempferol, quercetin) and flavones (apigenin, luteolin), which further confirmed that *Ct*UGT52 might be involved in the biosynthesis of chalcone glycosides and other various flavonoid glycosides in yellow safflower accession. *Ct*UGT50 was demonstrated to exhibit glycosylation activity specifically towards luteolin, catalyzing the glycosylation at the 7-OH position, suggesting that *Ct*UGT50 might involve in the luteolin-7-*O*-glucoside biosynthesis of safflower. Two UGT71 family glycosyltransferases, *Ct*UGT51 and *Ct*UGT54, with substrate promiscuity were identified and their activities were found to be similar *in vitro*. They might participate in the biosynthesis of flavonols and flavones monoglucosides in safflower. *Ct*UGT56 and *Ct*UGT57 could only catalyze kaempferol to produce Astragalin. *Ct*UGT55, which belonged to UGT708 family, could catalyze the glycosylated reaction of 2-Hydroxynaringenin (Table 2).

**Table 2:** Analysis of the enzymatic reaction of recombinant *Ct*UGTs.

Name	Naringenin chalcone	Phloretin	Kaempferol	Quercetin	Apigenin	Luteolin	2-Hydroxy naringenin
*Ct*UGT50	−	−	−	−	−	**+**	−
*Ct*UGT51	−	−	**+**	**+**	**+**	**+**	**+**
*Ct*UGT52	**+**	**+**	**+**	**+**	**+**	**+**	−
*Ct*UGT53	−	−	−	−	−	−	−
*Ct*UGT54	−	−	**+**	−	**+**	**+**	**+**
*Ct*UGT55	−	−	−	−	−	−	**+**
*Ct*UGT56	−	−	**+**	−	−	−	−
*Ct*UGT57	−	−	−	**+**	−	−	−

‘+’ indicated that the substrate can be catalyzed by *Ct*UGTs to form the corresponding glycosides.

#### Relative expression levels of *CtUGT52* during different developmental stages and its functional studies *in vivo*

Quantitative Real-time PCR analysis indicated that the expression level of *CtUGT52* was relatively low from the Y_I to Y_II stage in yellow safflower, while it significantly increased during the Y_III stage and continued to rise in the Y_IV stage (as shown in Fig. S12). It values that the pattern was found to be consistent with the accumulation trend of HSYA.

**Figure 6:**
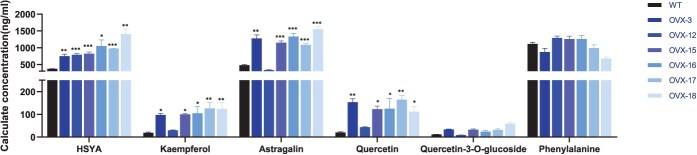
The accumulation of flavonoid derivatives in *CtUGT52-ovx* transgenic plants. (Mean ± SEM (*n* = 3), ^*^P < 0.05, ^**^P < 0.01, ^***^P < 0.001).

Making use of *Agrobacterium*-mediated transformation, six transgenic safflower plants which overexpressed *CtUGT52* were constructed and identified. The morphology of transgenic plants showed no difference compared with the wild-type (as shown in Fig. S13). Quantitative detection analysis showed that HSYA content in *CtUGT52*-overexpressed safflower had significant increased 2.01 to 3.81 times compared with wild-type safflower (as shown in Fig. 6). Astragalin was observed increasing up to 2.25-fold. The content of isoquercetin did not show a significant soar. While the important precursor for the flavonoids biosynthesis, phenylalanine, showed no significant difference between *CtUGT52*-overexpressed and wild-type plants. These confirmed that *Ct*UGT52 promote the glycosylation of flavonoids *in vivo*, resulting in an increased accumulation of chalcone and flavonol glycosides in safflower.

### Catalytic mechanism of *Ct*UGT52

**Figure 7:**
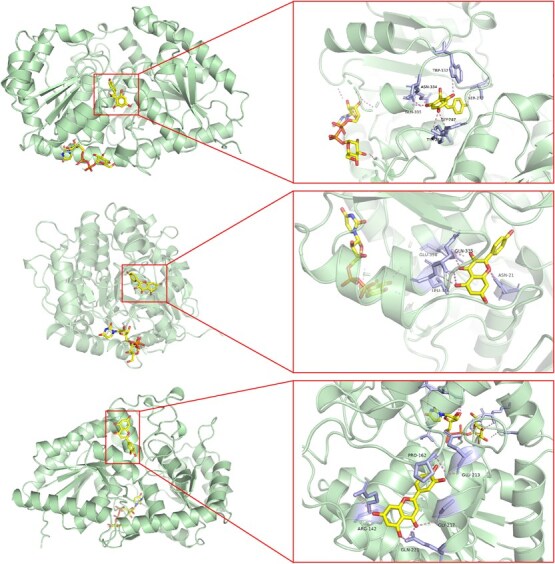
Analysis of key sites of Phloretin/Kaempferol/Luteolin and UDP-glucose binding to *Ct*UGT52.

Simulating and visualizing the interactions of *Ct*UGT52 with different types of flavonoids (phloretin, kaempferol, and luteolin) and UDP-glucose simultaneously using AutoDockVina and PyMOL. As shown in Fig. 7, the results indicated a binding free energy of −11.44 kcal/mol for the interaction of *Ct*UGT52 with phloretin and UDP-glucose, −11.84 kcal/mol for the interaction of *Ct*UGT52 with kaempferol and UDP-glucose. Y246, G247, S277, W332, D334, and Q335 of *Ct*UGT52 showed interactions with phloretin, D21, Q335, L336, and E358 showed interactions with kaempferol, while R142, P162, E213, G217, and Q221 showed interactions with luteolin. Based on the docking results, all *Ct*UGT52 mutants were cloned and expressed.

Further site-directed mutagenesis experiments (as shown in Fig. S14) revealed the responsible residues for the binding of *Ct*UGT52 with phloretin were S277 and Q335, with kaempferol at residues Q335 and E358, with luteolin at residues P162 and E213, respectively.

## Discussion

Until now, a *C*-glycosyltransferase that involved in the biosynthesis of HSYA has been elucidated recently while most *Ct*UGTs were charactered as flavonol or flavonoid *O*-glucosyltransferases [11, 24–27]. And it has become increasingly prevalent to further hone in on the targets genes through multi-omics analysis, enabling a more precise prediction of candidate genes and metabolic pathways for subsequent biological validation [28].

In this study, the construction of near-isogenic gene pools for the two extreme traits of yellow and white safflower from two parent strains (ZHH0119 and XHH007) laid a solid foundation for effectively filtering out the genes associated with the safflower color trait and the biosynthesis of chalcone glycosides, which localized to five regions on chromosomes 2 (11.4–31.2 Mb), 8 (12.5–19.9 Mb), 9 (10.6–16.4 Mb), 10 (0.002–0.083 Mb) and 12 (58.5–62.1 Mb) based on Δ(SNP-index). Combined with gene function annotations, flavonoid biosynthesis genes which most likely directly related to the yellow corolla of safflower were screened out, including 6 *CtPALs*, 3 *CtC4Hs*, 2 *Ct4CLs,* and 1 *CtCHS*, structural modification enzymes including 32 *CtUGTs* and 70 *CtCYPs*, as well as transcription regulatory factors encoding genes including 67 *CtbHLHs*, 248 *CtMYBs*, 101 *CtWRKYs*, and 50 *CtWD40s*. These results revealing the biosynthesis of chalcone glycosides could be regulated by multiple genes, aiding in the development of molecular markers for safflower and guiding safflower breeding efforts.

To further elucidate the genetic basis of chalcone glycoside biosynthesis in safflower, we constructed profiles of DEGs and DEMs from two safflower accessions with distinct floral colors. Focusing on the same flowering stage, the *CtUGTs* exhibited significant differential expression in yellow accession compared to the white implying their potential involvement in the biosynthesis of chalcone glycosides. Furthermore, metabolic profiling revealed that the content of HSYA increased dramatically from the Y_II to Y_III flowering stages in the yellow accession. This pronounced HSYA accumulation pattern, coupled with the core logic linking floral color variation to stage-specific metabolic shifts, prompted us to extend the analysis to DEGs across different flowering stages within the yellow accession. This two-tiered screening strategy—cross-accession and within-accession—enabled the precise identification of target *CtUGTs* associated with HSYA conversion and accumulation in yellow safflower.

Based on the aforementioned analysis and experiment, we have preliminarily demonstrated the *Ct*UGTs which involved in the biosynthetic pathways of different flavonoid glycosides in yellow safflower (as shown in Fig. S15).

Most glycosyltransferases are characterized by a broad substrate promiscuity and current research on flavonoid UGTs mainly focuses on 3-O-GT and 7-O-GT [27]. For example, there has been reported that a *Ct*OGT1 could catalyze the 7-O-mono-glycosylation of various flavonoid substrates and exhibited strong substrate affinity toward kaempferol [29]. While studies on the *in vitro* catalytic mechanism confirmed that the diverse amino acid sequence of *Ct*UGT52 endows it with more than one active site, thereby enabling its 3-O, 7-O, 3′-O, 4′-O-glycosylation activities, unveiling the versatile functions of safflower glycosyltransferases. More importantly, *Ct*UGT52 stimulate the formation of naringenin chalcone glycoside HSYA in safflower that could also catalyze the mono- and di-glycosylation of various chalcones, flavonols, and flavonoids. Since the aglycone of HSYA remains unavailable, we could not confirm whether *Ct*UGT52 was involved in the glycosylation of HSYA through *in vitro* assays. *In vitro* experiments demonstrated that *Ct*UGT52 could catalyze the glycosylation of naringenin chalcone, however, the experiment showed that glycosyltransferase expressed in different hosts exhibit inconsistent glycosylation functions due to its coiling and folding into specific tertiary structures, potentially leading to function discrepancies. Through *in vivo* and *in vitro* verification, combined with the expression pattern of *CtUGT52* which consistent with the accumulation levels of compound HSYA across various stages, we believed that *Ct*UGT52 has involved in the biosynthesis of HSYA.

The enzyme study allows for achieving the engineering or manipulation of biosynthetic pathways for the production of target metabolites in heterologous systems. The reconstitution of the glycosides biosynthesis from plants into microorganism offers a more sustainable production alternative to these high-value compounds. Based on the analysis and experiment above, the characterization of *Ct*UGTs in this study paved the way for their potential applications and development. Additionally, a deep understanding of UGT functional mechanisms are required by efficient manipulation of plant UGTs [30]. By identifying the key residues of the promiscuous glycosyltransferase *Ct*UGT52 through site-directed mutagenesis, we can target mutations to optimize its properties for specific purposes.

## Materials and methods

### Plant materials

The yellow safflower line (ZHH0119) was obtained from Safflower Germplasm Resource Bank of Xinjiang Academy of Agricultural Sciences in China, the white safflower variety (XHH007) was jointly bred by our team and the team led by research professor Yaohua Chen in Xinjiang Academy of Agricultural Sciences. Both lines(varieties) were repeatedly purified prior to the experiments. They were grown in the School of Pharmacy of Naval Medical University. After crossing the two parents reciprocally, resulting in the F_4_ generation of two accessions. Plants from both homozygous lines were grown in the greenhouse at 23°C ± 2°C under light of circadian rhythm (16-hour/8-hour light–dark cycle) respectively to construct two extreme pools for BSA-Seq. Four different growing stages of yellow safflower corolla (Y_I: 3 days before blooming, Y_II: flowering onset, Y_III: later day after blooming, Y_IV: 3 days after blooming) and white safflower corolla (W_III: later day after blooming) were collected for transcriptomic and metabolomic analysis.

### Extraction of genomic DNA and BSA-Seq analysis

The pools were constructed with the F_4_ population from two extreme-phenotype parental lines and two bulks (with 30 extreme-phenotype individuals) by mixing of high-quality genomic DNA. Parental lines serve as controls. Genomic DNA extraction and sequencing were performed on Illumina Hiseq 2000 at Shanghai Majorbio Bio-Pharm Technology Co., Ltd. Based on the alignment between clean reads and reference genome (PRJNA642978) by BWA (Version 0.7.10), then using Picard-tools to mark duplicates, using GATK to process InDel realignment and base recalibration successively. The SNPs and InDels were filtered with the GATK UnifiedGenotyper before further analysis with the following settings: QD < 2.0, MQ < 40.0, FS > 60.0, SOR > 3.0, MQRankSum < −12.5, ReadPosRankSum < −8.0.Δ(SNP-index) was performed by QTLseqr (https://github.com/bmansfeld/QTLseqr) to screen out QTLs and candidate genes.

### RNA sequencing and data analysis

For the five groups of two accessions corolla (three biological replicates were sampled per group), total RNA were extracted and sequenced at Shanghai Majorbio Bio-Pharm Technology Co., Ltd. The RNA Libraries were constructed using the Illumina TruSeq™ RNA Library Prep Kit. Sequencing was performed using an Illumina NovaSeq 6000 sequencer (2 × 150 bp read length). Clean reads were aligned to the reference genome (PRJNA642978) using TopHat2 (http://tophat.cbcb.umd.edu/). By means of StringTie (http://ccb.jhu.edu/software/stringtie/), the mapped reads of each group were assembled and all transcripts including novel transcripts had been annotated.

### Differential expressed genes analysis

Gene expression was calculated with Fragments Per Kilobase of exon model per Million mapped fragments (FPKM). Based on the quantified expression data, using DESeq2 to perform differential analysis among groups. Differential expressed genes were identified with following parameters: |log2 (fold change)| ≥ 1, *P*-adjust ≤ 0.05. GO functional enrichment analysis of DEGs was performed using Goatools (https://github.com/tanghaibao/Goatools) and KEGG pathway enrichment analysis was performed using KOBAS.

### Weighted gene co-expression network analysis

Using RSEM to complete gene expression background correction and normalization, filtering out genes with anomalies or low coefficients of variation and ensure the correlation strengths between processed genes conform to a scale-free distribution. After preprocessing, classifying genes and transcripts and clustering genes and transcripts with similar expression patterns into modules. Conducting identification for each module, as well as module correlation and module clustering analysis. Set the soft threshold β to 14, the module merging threshold to 30, the minKMEtoStay value to 0.3, and the merge cut height to 0.25. Calculate the correlation coefficients between modules and different varieties and flowering times using the Spearman correlation coefficient method.

### Targeted metabolomic analysis

The collected corolla of each group for RNA sequencing were also used for metabolite extractions. Each sample was dried and ground into powder and extracted with 60% methanol before UHPLC-Q-TOF/MS analysis. Targeted metabolic profiling comprising 13 chemical standards which purchased from Aladdin Reagent (Shanghai) Co., Ltd or Shanghai yuanye Bio-Technology Co., Ltd. UHPLC-Q-TOF/MS analysis were carried out for each sample on an Agilent 6538 Accurate Mass Quadrupole Time-of-Flight mass spectrometer (Agilent, Santa Clara, CA, USA). XBridge TM BEH C18 column (2.5 μm, 2.1 × 100 mm; Waters, Milford, MA) was used for chromatographic separations. Metabolites data were proceed with Qualitative Analysis B.06.00 software (Agilent).

### Untargeted metabolomic analysis

Untargeted metabolic profiles were measured at Shanghai Majorbio Bio-Pharm Technology Co., Ltd. Data pre-procession was accomplished through missing value recoding and normalization. All metabolites had been annotated after alignment with KEGG COMPOUND and HMDB databases. The R package ‘ropls’ (Version 1.6.2) was used to perform principal component analysis (PCA). Differential metabolites among two groups were mapped into their biochemical pathways through metabolic enrichment and pathway analysis based on KEGG database (http://www.genome.jp/kegg/). The Python package ‘scipy.stats’ (https://docs.scipy.org/doc/scipy/) was used to perform enrichment analysis to obtain the most relevant biological pathways for experimental treatments.

### Recombinant *Ct*UGTs expression and purification in *Escherichia coli*

The full length of the *CtUGTs* were amplified using designed primers (Table S4) and inserted into the pET-28a (+) vector (Weidi). Recombinant plasmid pET-28a(+)-*CtUGTs* were transformed to Rosetta(DE3) chemically competent cell (Weidi) and expressed by IPTG. The recombinant protein was purified according to previously reported procedures and analyzed using SDS-PAGE. The purified proteins were concentrated and stored for *in vitro* assays.

### Enzymatic assays

The reaction system contained 80 μL Tris–HCl buffer (pH 7.4), 50 μg recombinant protein, 1 mM UDP-Glc and 100 μM of sugar receptors, incubated at 30°C for 2 h and added 100 μL of methanol to end the reaction.

### Quantitative real-time PCR

The cDNA was synthesized from 1 μg of total RNA using the PerfectStart® Green qPCR SuperMix (TransGen Biotech) according to the protocol. The qRT-PCR was conducted with the diluted cDNA on a QuantStudio™3 (ThermoFisher Scientific). *Ct60S* was employed as an endogenous control gene to normalize the gene expression data and to calculate the 2^−ΔΔCT^ values. Each sample was subjected to three separate experimental replicates. The primers sequences were provided in Table S1.

### Construction and determination of *CtUGT52* overexpressed safflower

Using pMT39 (pCAMBIA-1380-CaMV35S-MCS-EGFP-NOS) which purchased from ShiAo (Shanghai) Biotechnology Co., Ltd as the expression vector [5, 31] to construct *CtUGT52* overexpression plasmid and through *Agrobacterium*-mediated pollen tube pathway method to breed overexpressed *CtUGT52* safflowers. After the infiltration treatment, selected 30–40 seeds of fully mature plants and sow them under greenhouse conditions (16 hours of light and 8 hours of darkness). Collected fresh leaves from the plants and extracted total DNA. Using wild-type safflower as negative control for the identification of positive plants.

### Flavonoids content quantification of *CtUGT52* overexpressed safflower

The sample treatment and analysis method were consistent with those in ‘Targeted metabolomic analysis’.

### Site-directed mutagenesis experiment

Mut Express® II Fast Mutagenesis Kit V2 was purchased from Vazyme Biotech Co., Ltd. Mutant primer sequences were listed in the Table S5. The recombinant *Ct*UGT mutants were obtained using the same methods described in ‘Recombinant *Ct*UGTs expression and purification in *Escherichia coli*.’ .

### Statistical analysis

The data were presented as standard error of the mean analyzed using SPSS statistic 17.0 software. One-way ANOVA was performed to determine the statistical significance of differences between groups. *P* < 0.05 was regarded as statistically significant.

## Supplementary Material

Web_Material_uhag068

## Data Availability

The transcriptome data have been uploaded on NCBI under BioProject: PRJNA1262321.
