# Supplementary material for: BSA-seq integrated with transcriptomics and metabolomics revealing the candidate genes associated with safflower colors and flavonoid glycosides biosynthesis
Source: Hortic Res. 2026 Mar 4;13(6):uhag068. doi: 10.1093/hr/uhag068 (PMC13253347; doi:10.1093/hr/uhag068)
Supplement: Web_Material_uhag068 [file web_material_uhag068.zip › Figure S3. Functional and Enrichment analysis of DEGs..pdf]

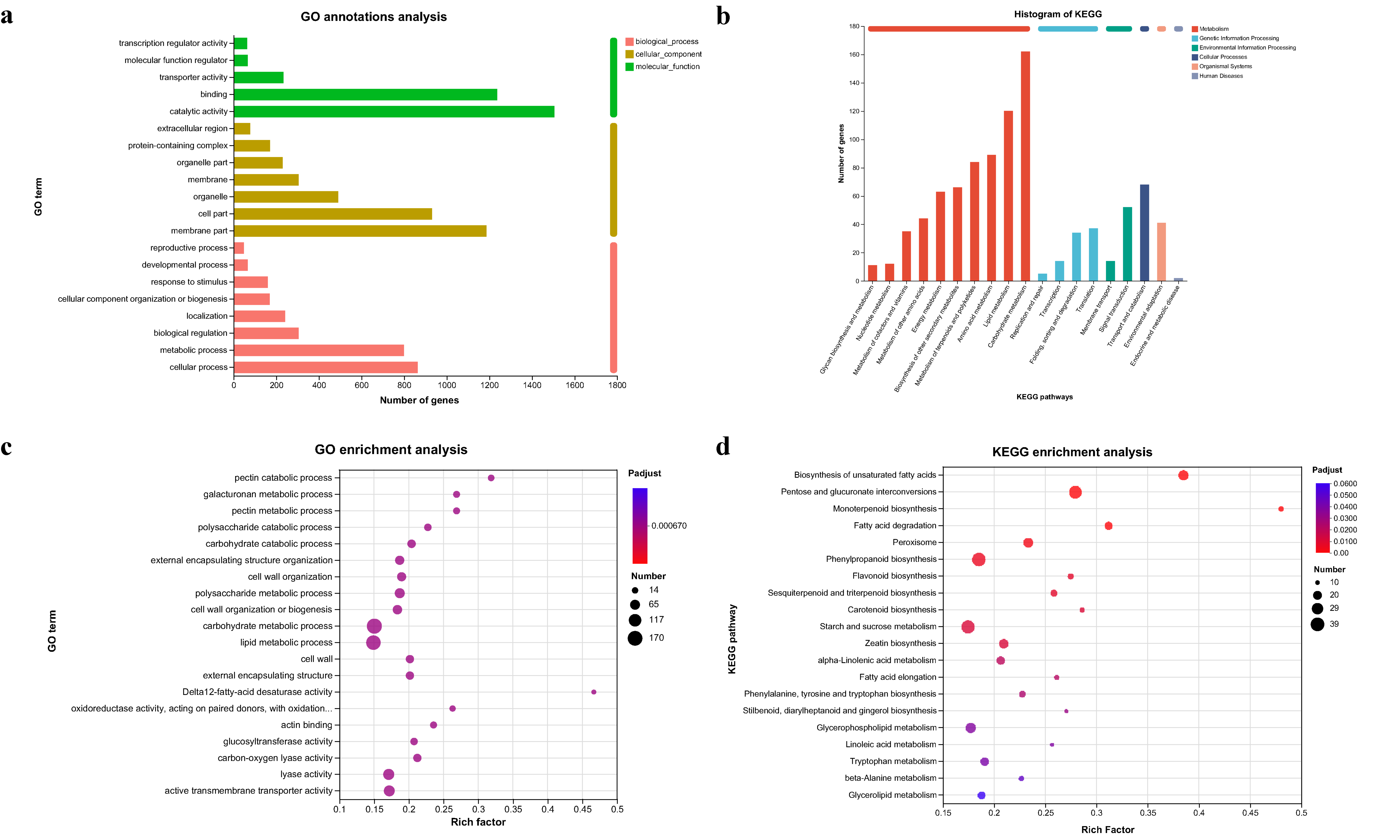

a
GO annotations analysis
transcription regulator activity
molecular function regulator
transporter activity
binding
catalytic activity
extracellular region
protein-containing complex
organelle part
membrane
organelle
cell part
membrane part
reproductive process
developmental process
response to stimulus
cellular component organization or biogenesis
localization
biological regulation
metabolic process
cellular process
biological_process
cellular_component
molecular_function
GO term
Number of genes
b
Histogram of KEGG
Metabolism
Genetic Information Processing
Environmental Information Processing
Cellular Processes
Organismal Systems
Human Diseases
Number of genes
Glycan biosynthesis and metabolism
Nucleotide metabolism
Metabolism of cofactors and vitamins
Metabolism of other amino acids
Energy metabolism
Biosynthesis of other secondary metabolites
Metabolism of terpenoids and polyketides
Amino acid metabolism
Lipid metabolism
Carbohydrate metabolism
Replication and repair
Transcription
Folding, sorting and degradation
Translation
Membrane transport
Signal transduction
Transport and catabolism
Environmental adaptation
Endocrine and metabolic disease
KEGG pathways
c
GO enrichment analysis
pectin catabolic process
galacturonan metabolic process
pectin metabolic process
polysaccharide catabolic process
carbohydrate catabolic process
external encapsulating structure organization
cell wall organization
polysaccharide metabolic process
cell wall organization or biogenesis
carbohydrate metabolic process
lipid metabolic process
cell wall
external encapsulating structure
Delta12-fatty-acid desaturase activity
oxidoreductase activity, acting on paired donors, with oxidation...
actin binding
glucosyltransferase activity
carbon-oxygen lyase activity
lyase activity
active transmembrane transporter activity
GO term
Rich factor
Padjust
0.000670
Number
14
65
117
170
d
KEGG enrichment analysis
Biosynthesis of unsaturated fatty acids
Pentose and glucuronate interconversions
Monoterpenoid biosynthesis
Fatty acid degradation
Peroxisome
Phenylpropanoid biosynthesis
Flavonoid biosynthesis
Sesquiterpenoid and triterpenoid biosynthesis
Carotenoid biosynthesis
Starch and sucrose metabolism
Zeatin biosynthesis
alpha-Linolenic acid metabolism
Fatty acid elongation
Phenylalanine, tyrosine and tryptophan biosynthesis
Stilbenoid, diarylheptanoid and gingerol biosynthesis
Glycerophospholipid metabolism
Linoleic acid metabolism
Tryptophan metabolism
beta-Alanine metabolism
Glycerolipid metabolism
KEGG pathway
Rich Factor
Padjust
0.0600
0.0500
0.0400
0.0300
0.0200
0.0100
0.00
Number
10
20
29
39
