# Supplementary material for: BSA-seq integrated with transcriptomics and metabolomics revealing the candidate genes associated with safflower colors and flavonoid glycosides biosynthesis
Source: Hortic Res. 2026 Mar 4;13(6):uhag068. doi: 10.1093/hr/uhag068 (PMC13253347; doi:10.1093/hr/uhag068)
Supplement: Web_Material_uhag068 [file web_material_uhag068.zip › Figure S10. SDS-PAGE of the His-tagged CtUGTs..pdf]

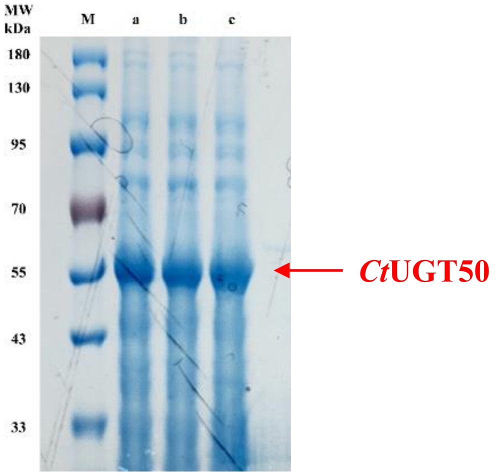

Figure S10. SDS-PAGE of the His-tagged *Ct*UGT50.

M: Marker; a-c: IPTG-induced protein expression bacterial supernatant

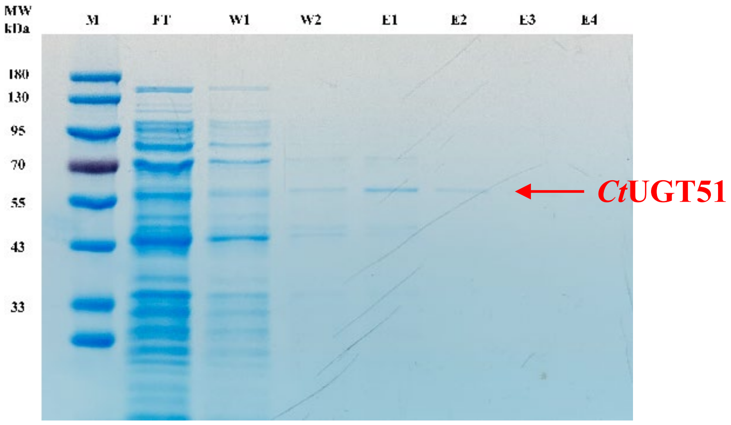

Figure S10. SDS-PAGE of the His-tagged *Ct*UGT51.

M: Marker; FT: Flow Through; W1-W2: Wash Solution 1-2; E1-E4: Elution Solution 1-4

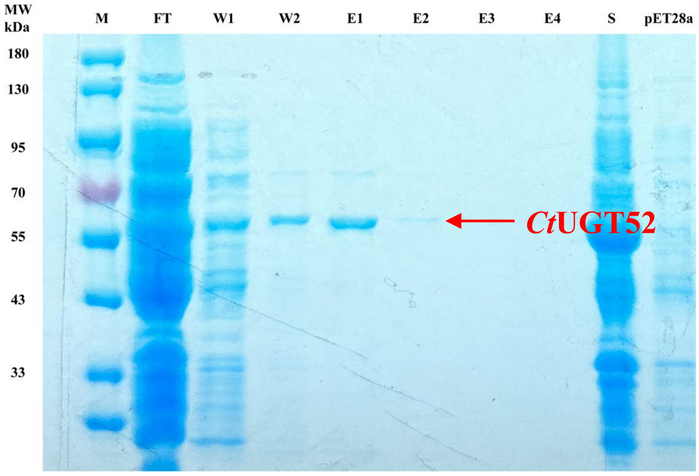

Figure S10. SDS-PAGE of the His-tagged *Ct*UGT52.

M: Marker; FT: Flow Through; W1-W2: Wash Solution 1-2; E1-E4: Elution Solution 1-4

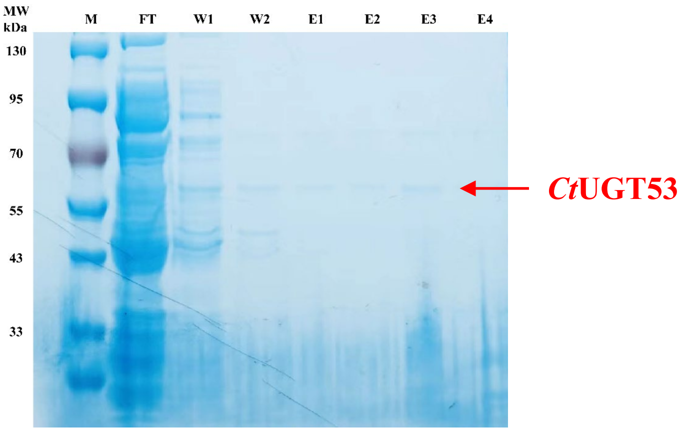

Figure S10. SDS-PAGE of the His-tagged *Ct*UGT53.

M: Marker; FT: Flow Through; W1-W2: Wash Solution 1-2; E1-E4: Elution Solution 1-4

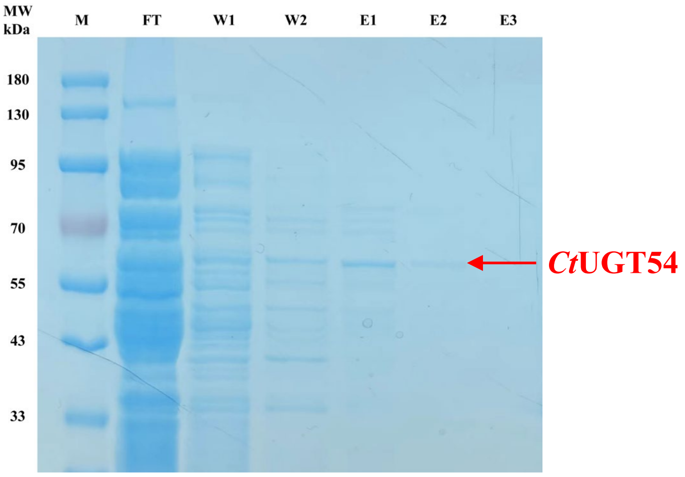

Figure S10. SDS-PAGE of the His-tagged *Ct*UGT54.

M: Marker; FT: Flow Through; W1-W2: Wash Solution 1-2; E1-E3: Elution Solution 1-3

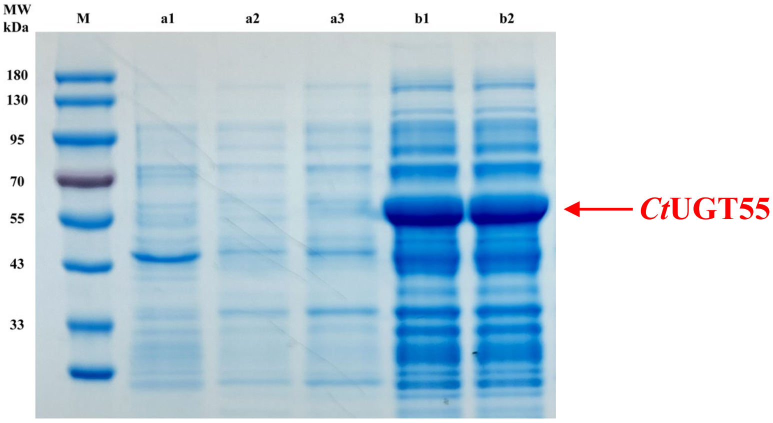

Figure S10. SDS-PAGE of the His-tagged *Ct*UGT55.

M: Marker; a1-a3: expression bacterial liquid of blank vector; b1-b2: expression bacterial liquid of *Ct*UGT55

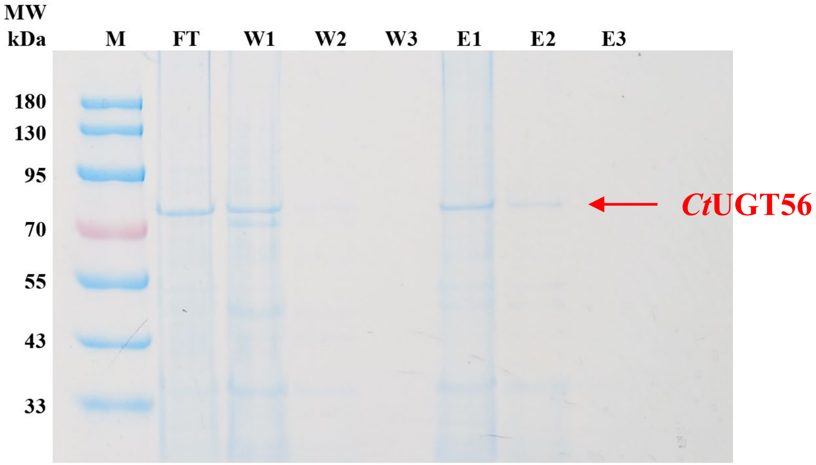

Figure S10. SDS-PAGE of the His-tagged *Ct*UGT56.

M: Marker; FT: Flow Through; W1-W3: Wash Solution 1-2; E1-E3: Elution Solution 1-3

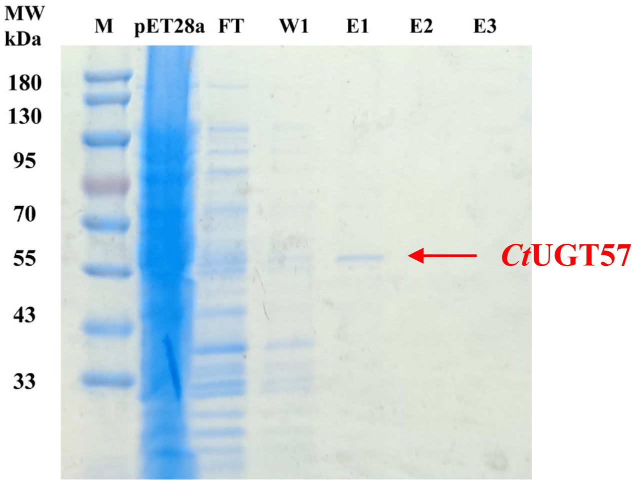

Figure S10. SDS-PAGE of the His-tagged *Ct*UGT57.

M: Marker; FT: Flow Through; W1-W2: Wash Solution 1-2; E1-E3: Elution Solution 1-3
