# Supplementary material for: BSA-seq integrated with transcriptomics and metabolomics revealing the candidate genes associated with safflower colors and flavonoid glycosides biosynthesis
Source: Hortic Res. 2026 Mar 4;13(6):uhag068. doi: 10.1093/hr/uhag068 (PMC13253347; doi:10.1093/hr/uhag068)
Supplement: Web_Material_uhag068 [file web_material_uhag068.zip › Figure S11. HPLC and LC-MSMS analysis of glycosylation of CtUGTs with different substrates..pdf]

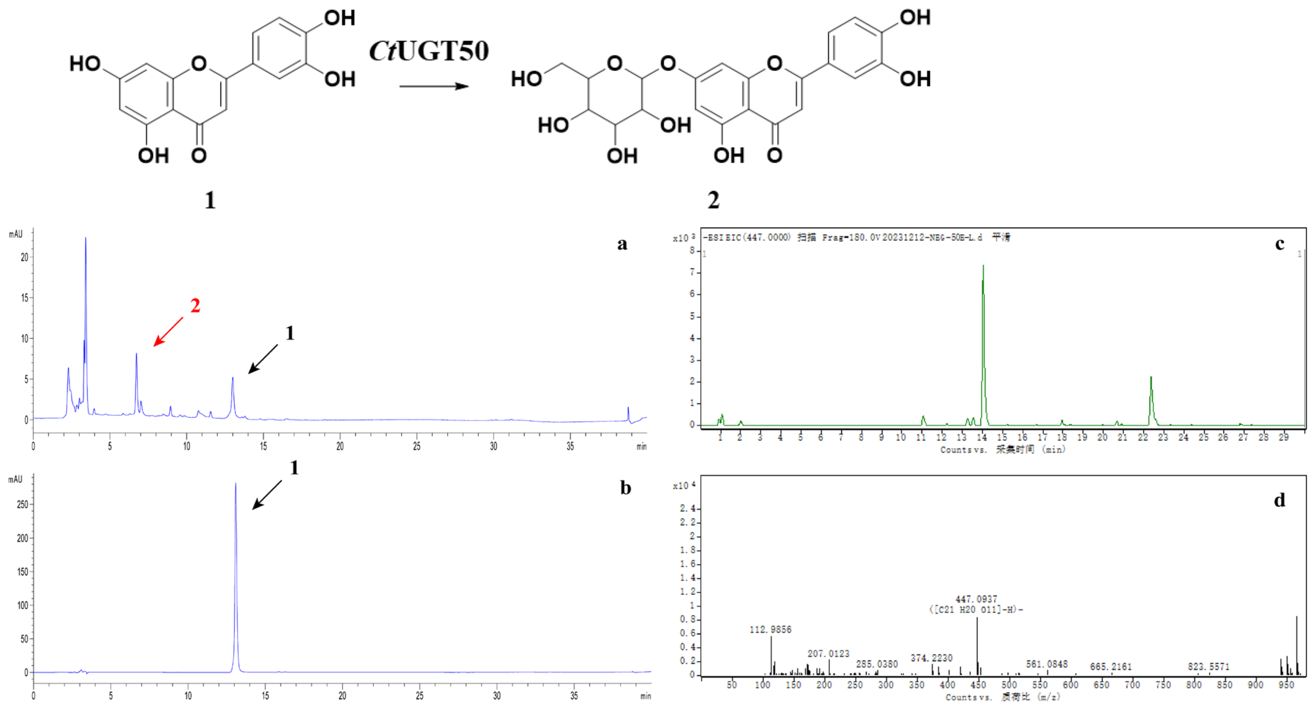

Figure S11. Results of Luteolin glycosylation reaction catalyzed by *Ct*UGT50.

a: HPLC result of *Ct*UGT50 (crude) after reaction with Luteolin (1) and UDP-glucose;

b: HPLC result of substrate and buffer with the absence of *Ct*UGT50;

c-d: LC-MS analysis of *Ct*UGT50 catalyzed reaction results

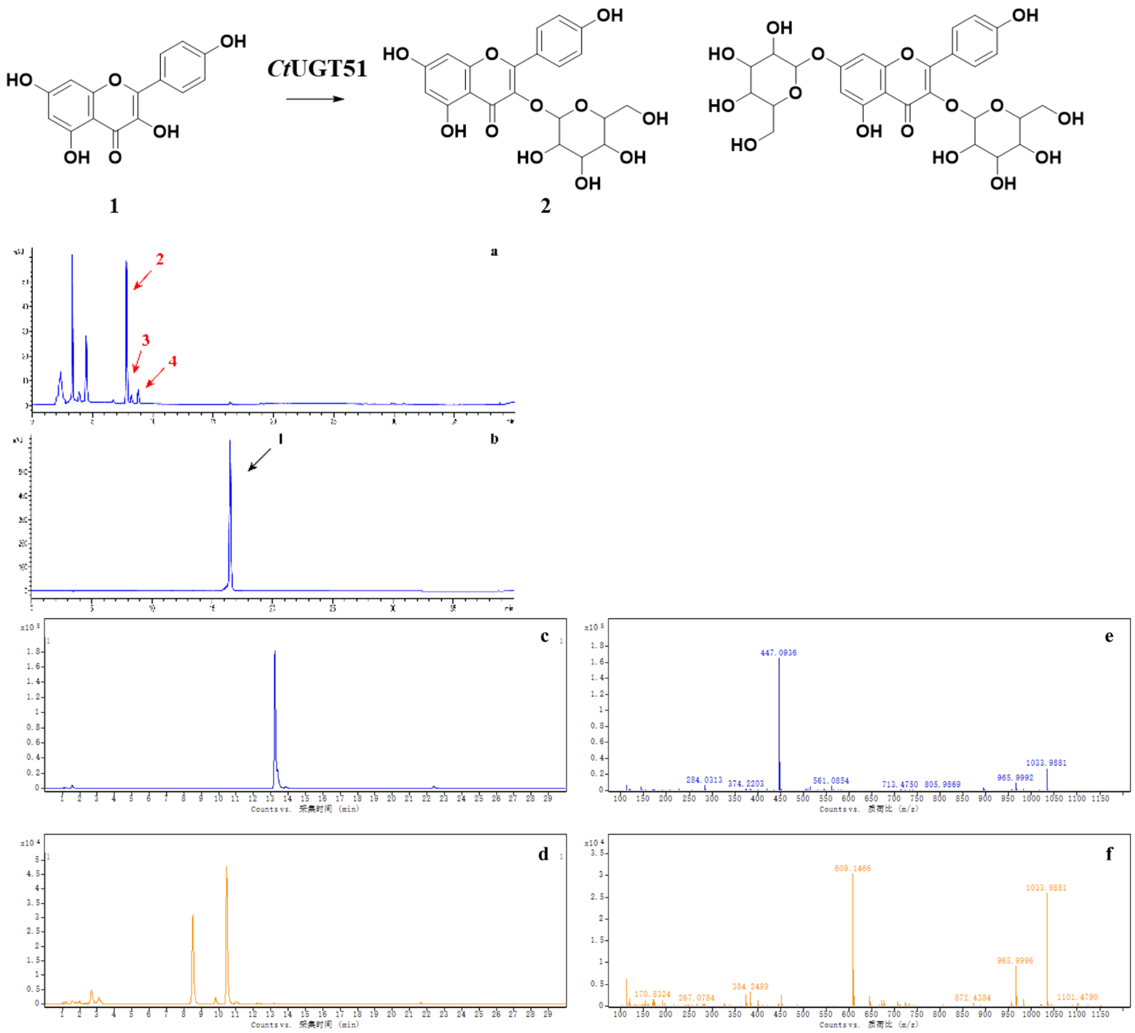

Figure S11. Results of Kaempferol glycosylation reaction catalyzed by *Ct*UGT51.

a: HPLC result of *Ct*UGT51 after reaction with Kaempferol (1) and UDP-glucose;

b: HPLC analysis of Kaempferol;

c-d: LC-MS analysis of *Ct*UGT51 catalyzed reaction results (Neg m/z 447);

e-f: LC-MS analysis of *Ct*UGT51 catalyzed reaction results (Neg m/z 609)

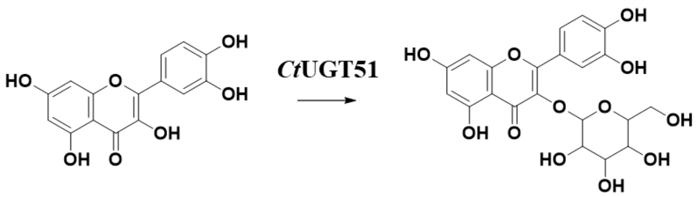

1 2

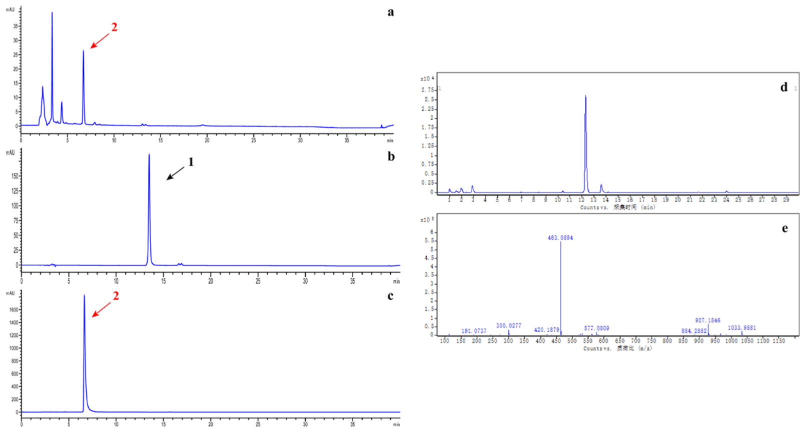

Figure S11. Results of Quercetin glycosylation reaction catalyzed by *Ct*UGT51.

a: HPLC result of *Ct*UGT51 after reaction with Quercetin (1) and UDP-glucose;

b-c: HPLC analysis of Quercetin (1) and Isoquercetin (2);

d-e: LC-MS analysis of *Ct*UGT51 catalyzed reaction results (Neg m/z 463)

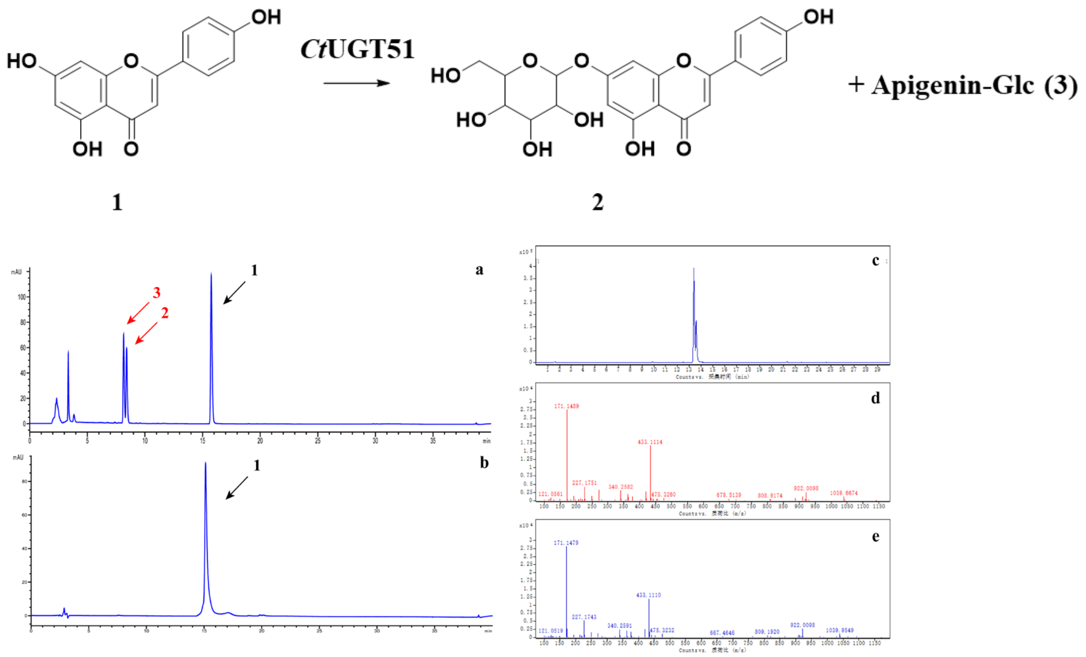

Figure S11. Results of Apigenin glycosylation reaction catalyzed by *Ct*UGT51.

a: HPLC result of *Ct*UGT51 after reaction with Apigenin (1) and UDP-glucose;

b: HPLC analysis of Apigenin;

c-e: LC-MS analysis of *Ct*UGT51 catalyzed reaction results (Pos m/z 433)

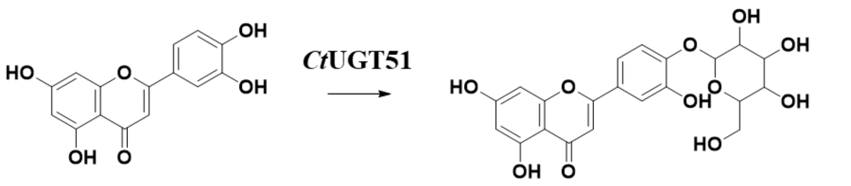

1 **2**

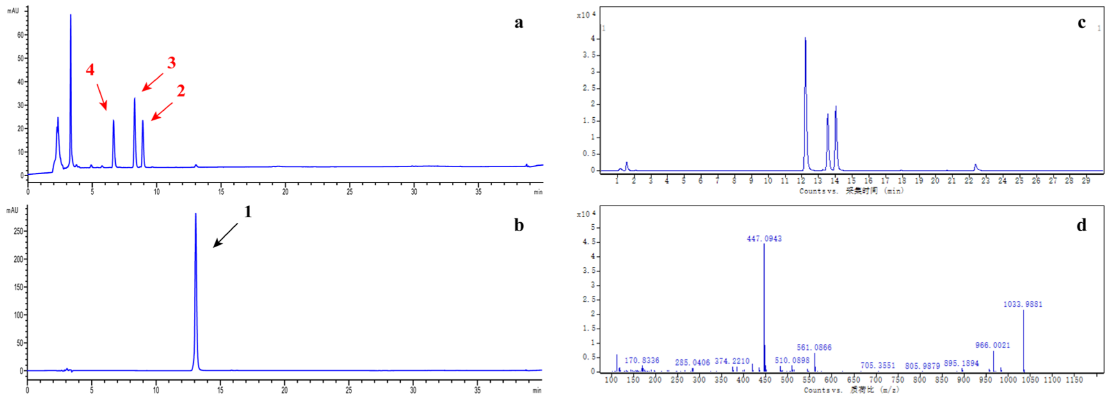

Figure S11. Results of Luteolin glycosylation reaction catalyzed by *Ct*UGT51.

a: HPLC result of *Ct*UGT51 after reaction with Luteolin (1) and UDP-glucose;

b: HPLC analysis of Luteolin;

c-d: LC-MS analysis of *Ct*UGT51 catalyzed reaction results (Neg m/z 447)

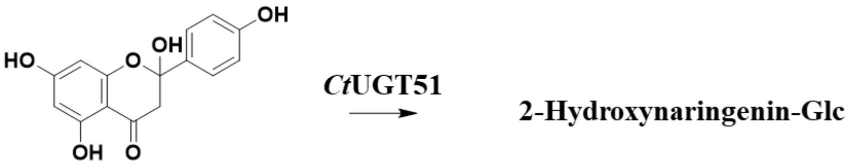

1 2

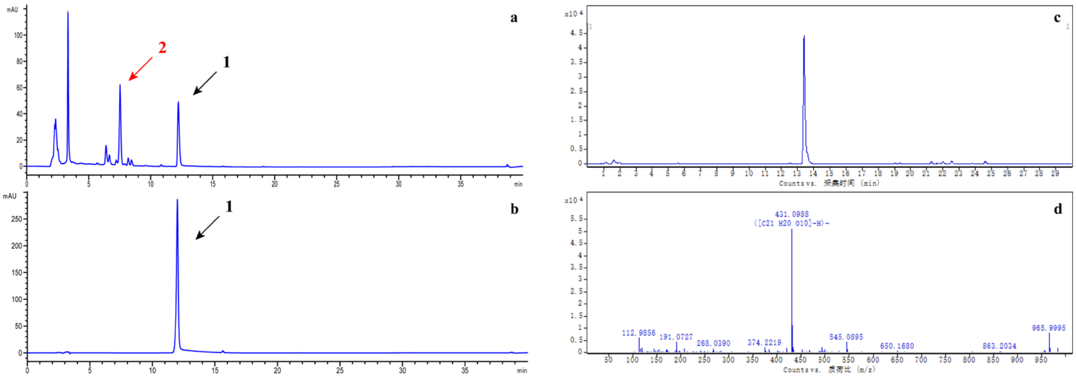

Figure S11. Results of 2-Hydroxynaringenin glycosylation reaction catalyzed by *Ct*UGT51.

a: HPLC result of *Ct*UGT51 reacted with 2-Hydroxynaringenin (1) and UDP-glucose;

b: HPLC analysis of 2-Hydroxynaringenin;

c-d: LC-MS analysis of *Ct*UGT51 catalyzed reaction results (Neg m/z 431)

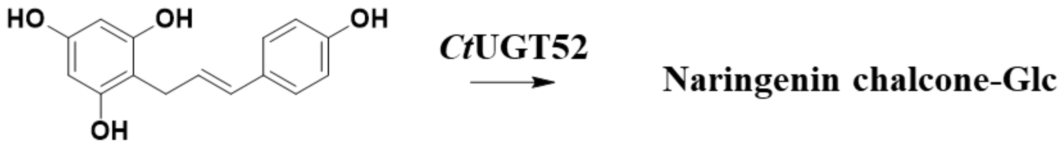

1 2

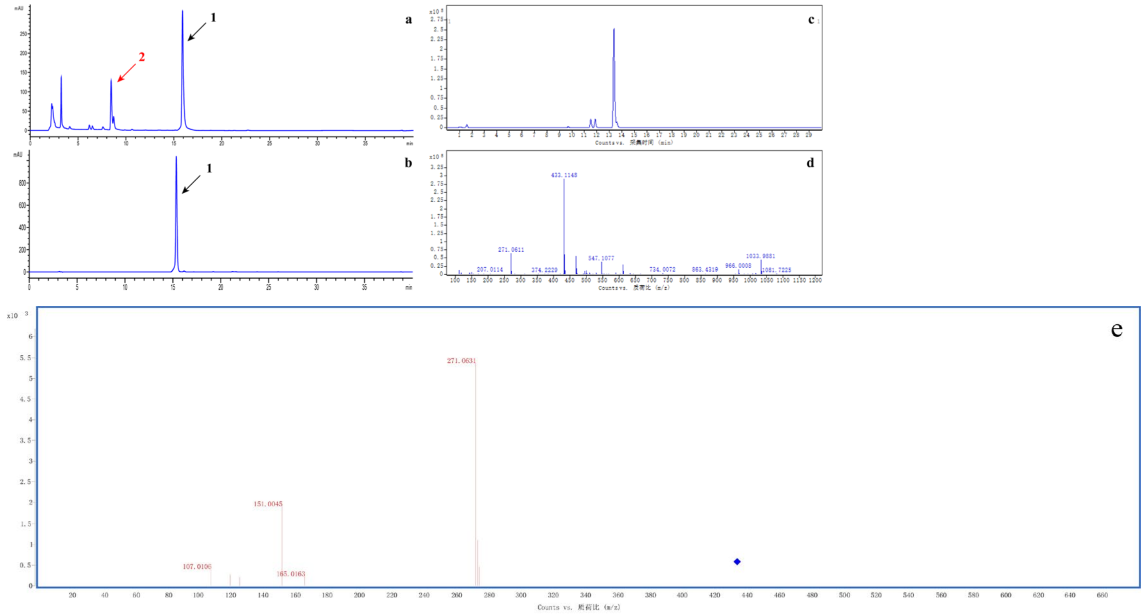

Figure S11. Results of Naringenin chalcone glycosylation reaction catalyzed by *Ct*UGT52.

a: HPLC result of *Ct*UGT52 after reaction with Naringenin chalcone (1) and UDP-glucose;

b: HPLC analysis of Naringenin chalcone;

c-d: LC-MS analysis of *Ct*UGT52 catalyzed reaction results (Neg m/z 433); e: LC-MS/MS analysis of compound 2

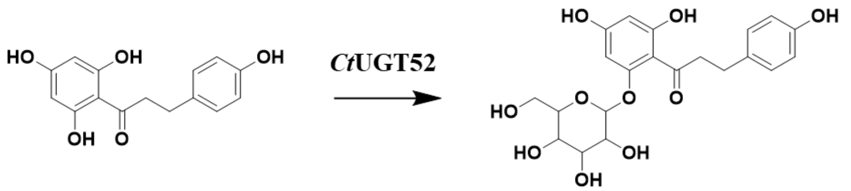

1   2

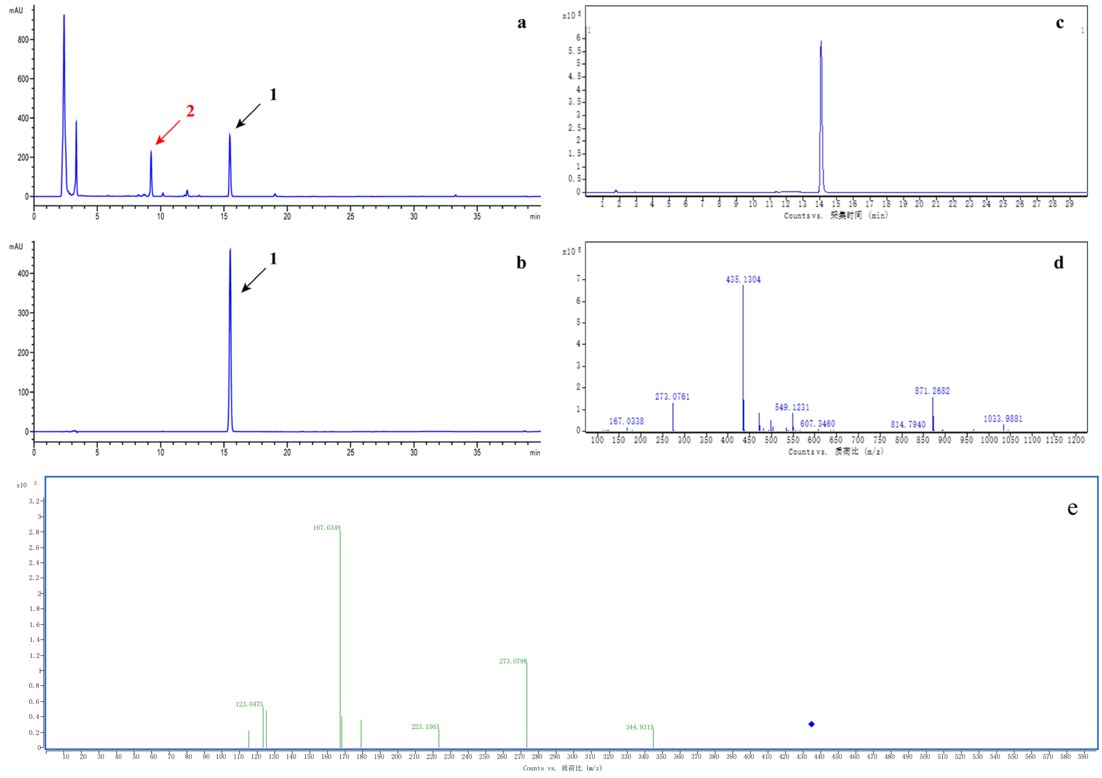

Figure S11. Results of Phloretin glycosylation reaction catalyzed by *Ct*UGT52.

a: HPLC result of *Ct*UGT52 after reaction with Phloretin (1) and UDP-glucose;

b: HPLC analysis of Phloretin;

c-d: LC-MS analysis of *Ct*UGT52 catalyzed reaction results (Neg m/z 435);

e: LC-MS/MS analysis of compound 2

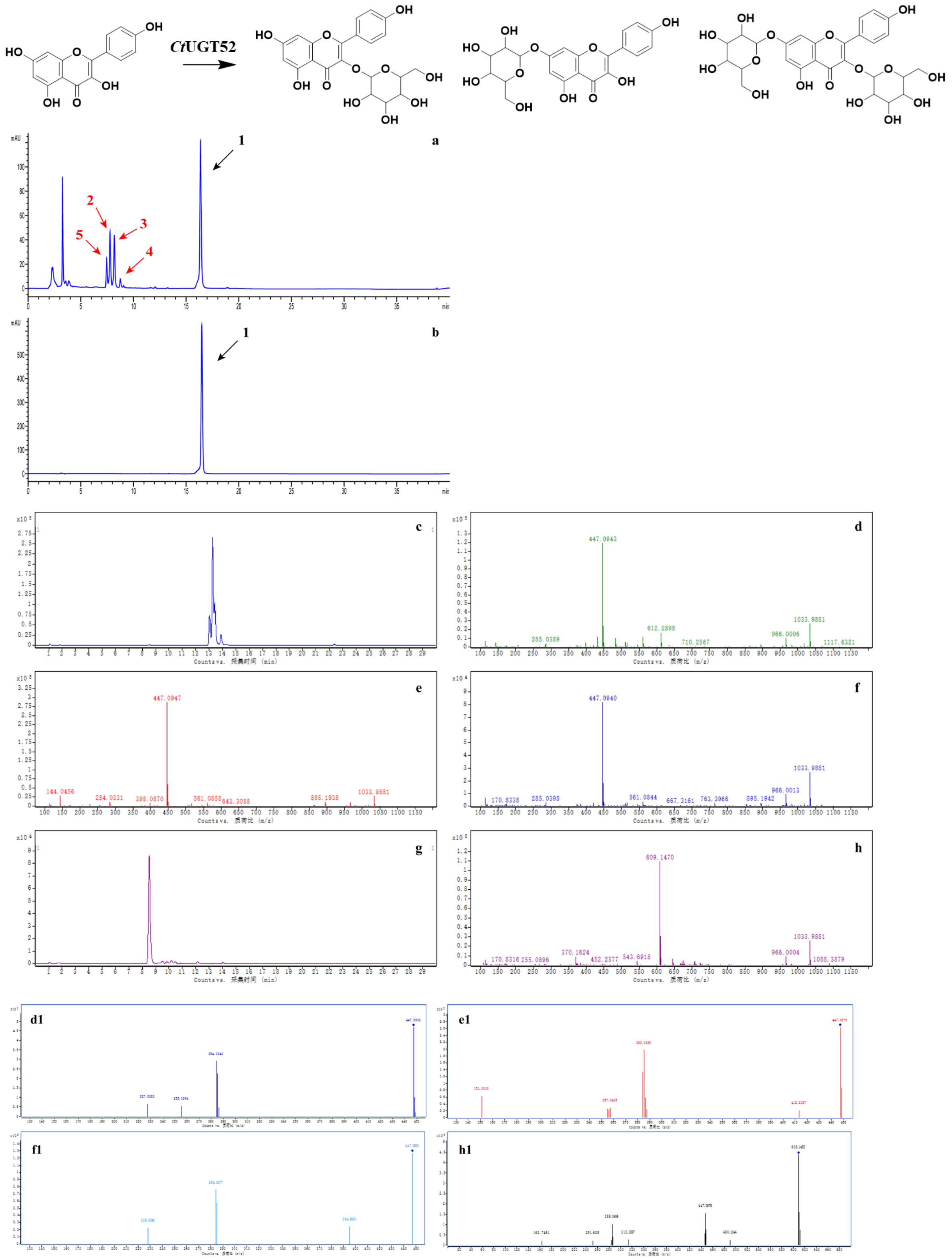

Figure S11. Results of Kaempferol glycosylation reaction catalyzed by *Ct*UGT52.

a: HPLC result of *Ct*UGT52 after reaction with Kaempferol (1) and UDP-glucose;

b: HPLC analysis of Kaempferol;

c-f: LC-MS analysis of *Ct*UGT52 catalyzed reaction results (Neg m/z 447);

g-h: LC-MS analysis of *Ct*UGT52 catalyzed reaction results (Neg m/z 609);

d1-h1: LC-MS/MS analysis of compound 2-5

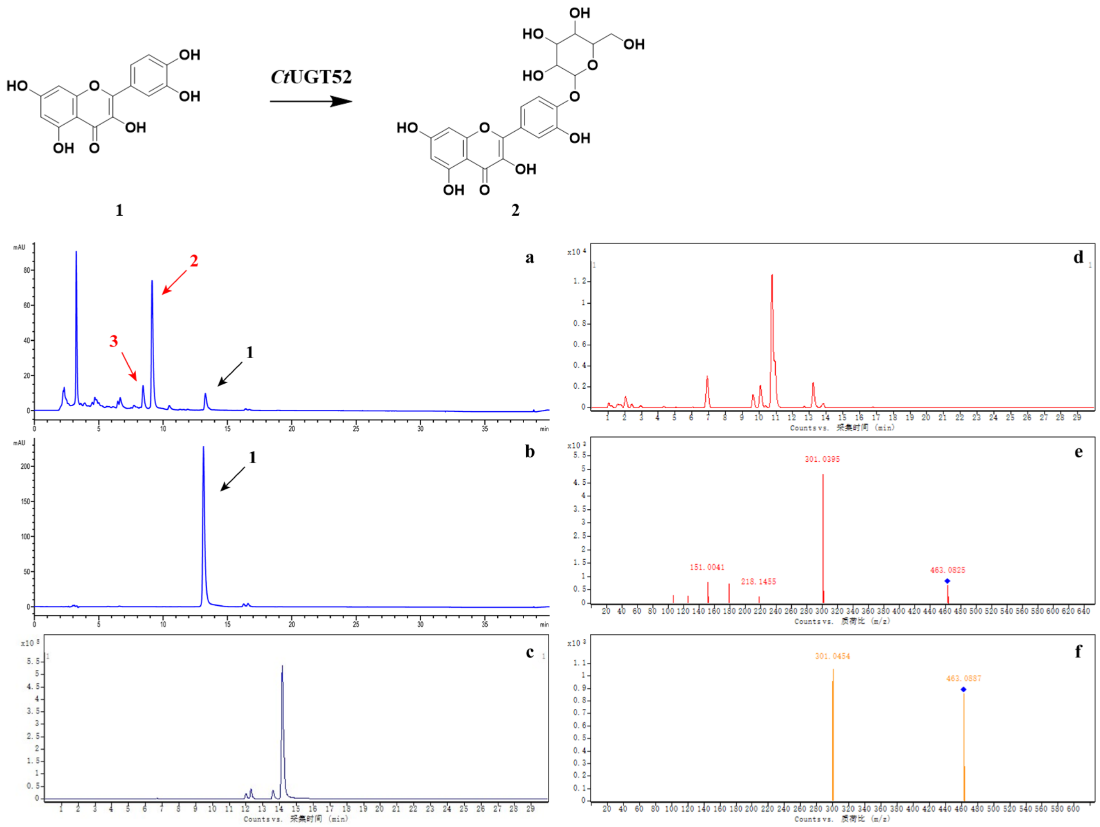

Figure S11. Results of Quercetin glycosylation reaction catalyzed by *Ct*UGT52.

a: HPLC result of *Ct*UGT52 after reaction with Quercetin (1) and UDP-glucose;

b: HPLC analysis of Quercetin;

c-d: LC-MS analysis of *Ct*UGT52 catalyzed reaction results (Neg m/z 463, 625);

e-f: LC-MS/MS analysis of *Ct*UGT52 catalyzed reaction results (Neg m/z 463)

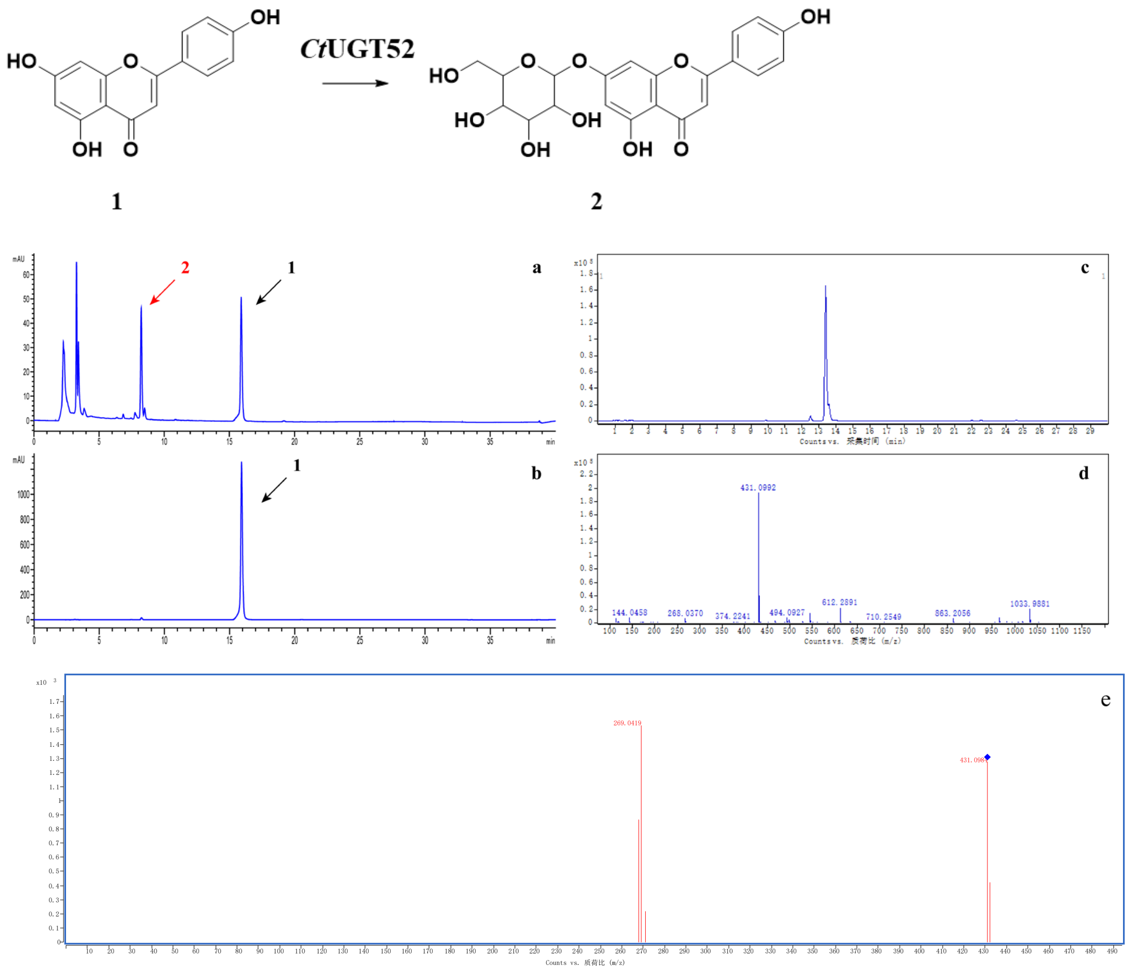

Figure S11. Results of Apigenin glycosylation reaction catalyzed by *Ct*UGT52.

a: HPLC result of *Ct*UGT52 after reaction with Apigenin (1) and UDP-glucose;

b: HPLC analysis of Apigenin;

c-d: LC-MS analysis of *Ct*UGT52 catalyzed reaction results (Neg m/z 431);

e: LC-MS/MS analysis of compound 2

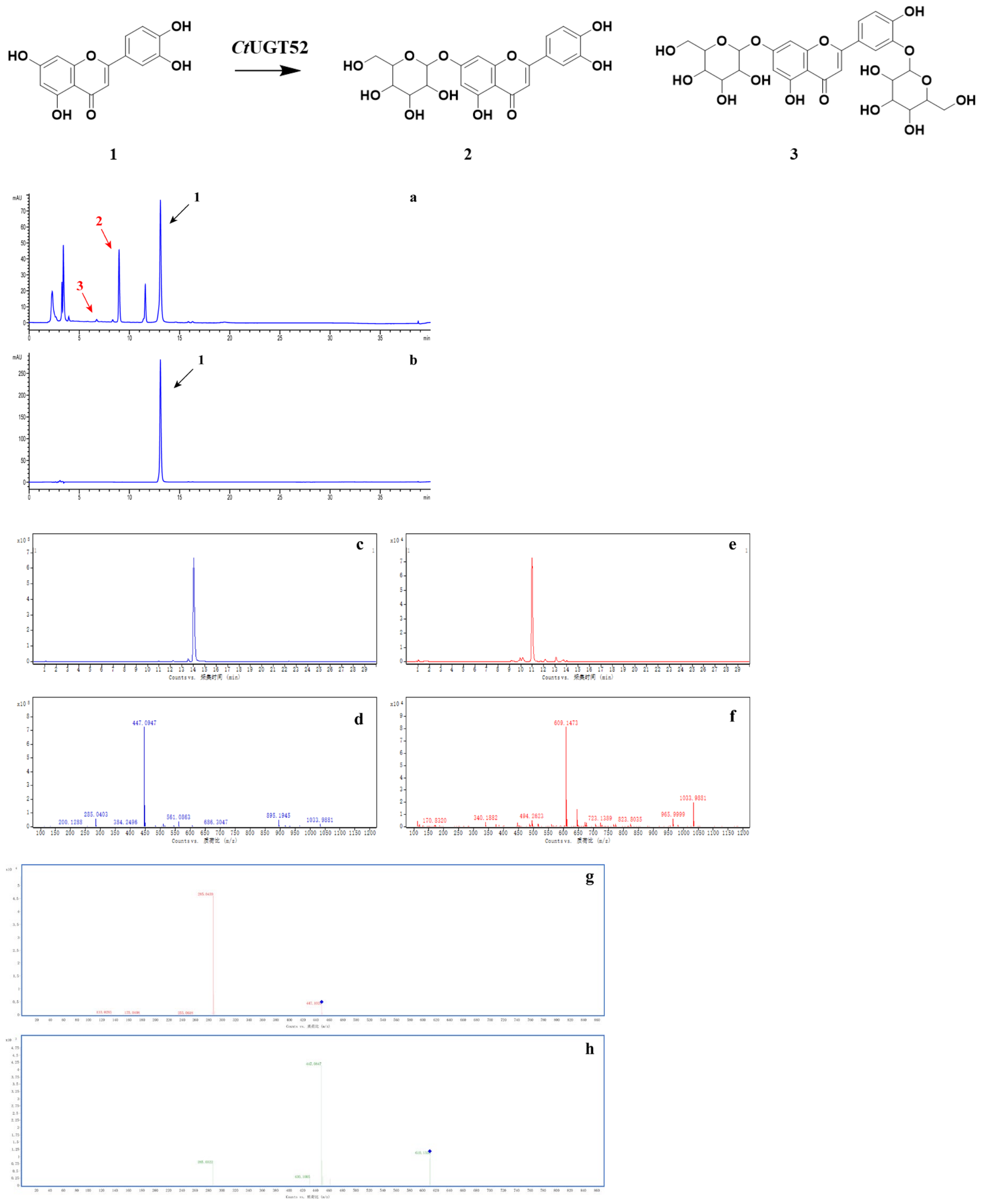

Figure S11. Results of Luteolin glycosylation reaction catalyzed by *Ct*UGT52.

a: HPLC result of *Ct*UGT52 after reaction with Luteolin (1) and UDP-glucose; b: HPLC analysis of Luteolin; c-d: LC-MS analysis of *Ct*UGT52 catalyzed reaction results (Neg m/z 447); e-f: LC-MS analysis of *Ct*UGT52 catalyzed reaction results (Neg m/z 609); g-h: LC-MS/MS analysis of compound 2 and 3

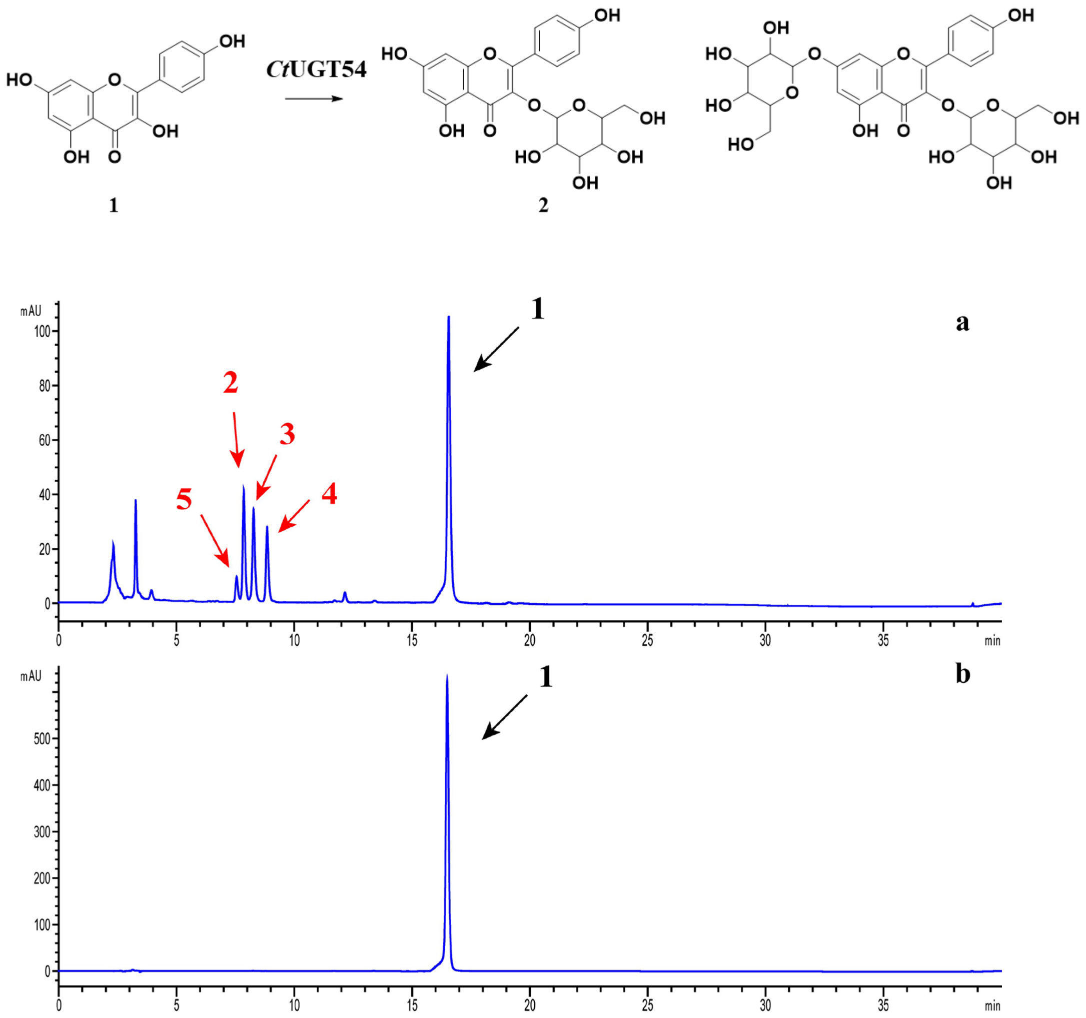

Figure S11. Results of Kaempferol glycosylation reaction catalyzed by *Ct*UGT54.

a: HPLC result of *Ct*UGT54 after reaction with Quercetin (1) and UDP-glucose;

b: HPLC analysis of Kaempferol;

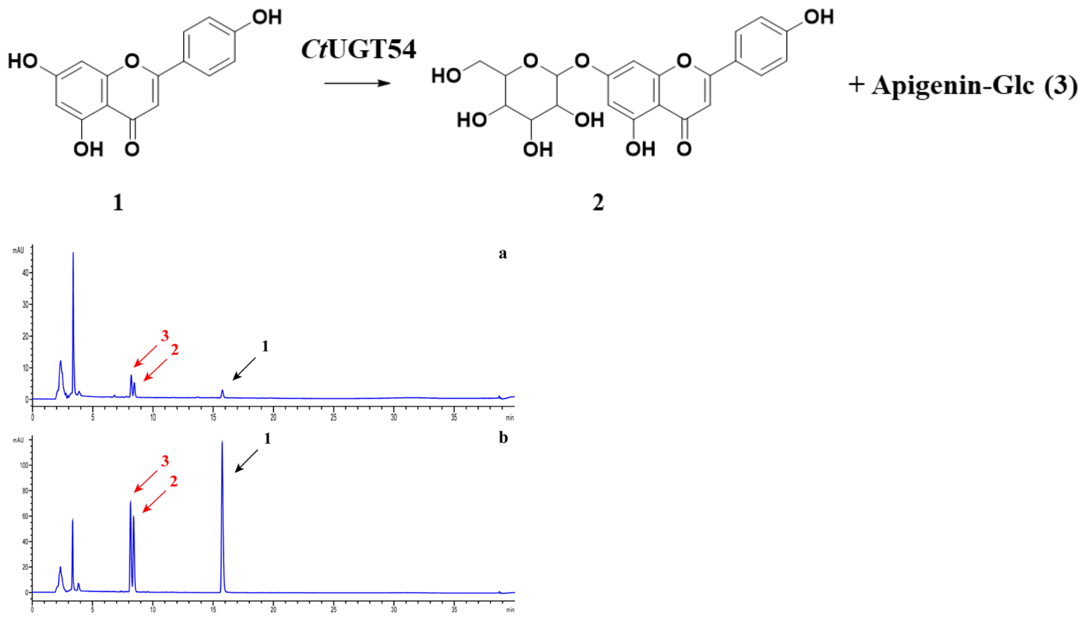

Figure S11. Apigenin glycosylation comparison between *Ct*UGT54 (a) and *Ct*UGT51 (b).

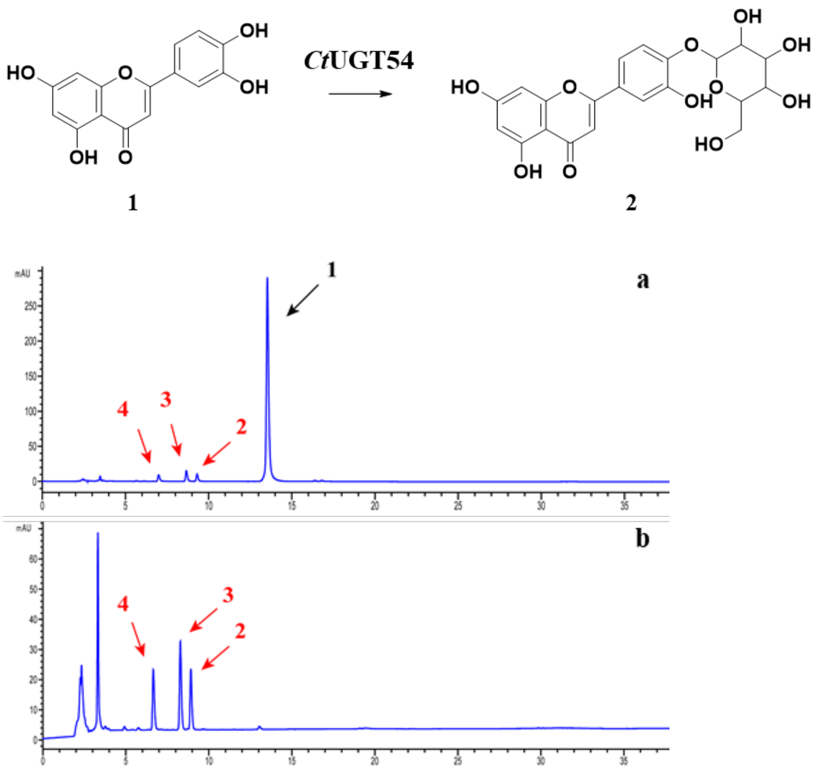

Figure S11. Luteolin glycosylation comparison between *Ct*UGT54 (a) and *Ct*UGT51 (b).

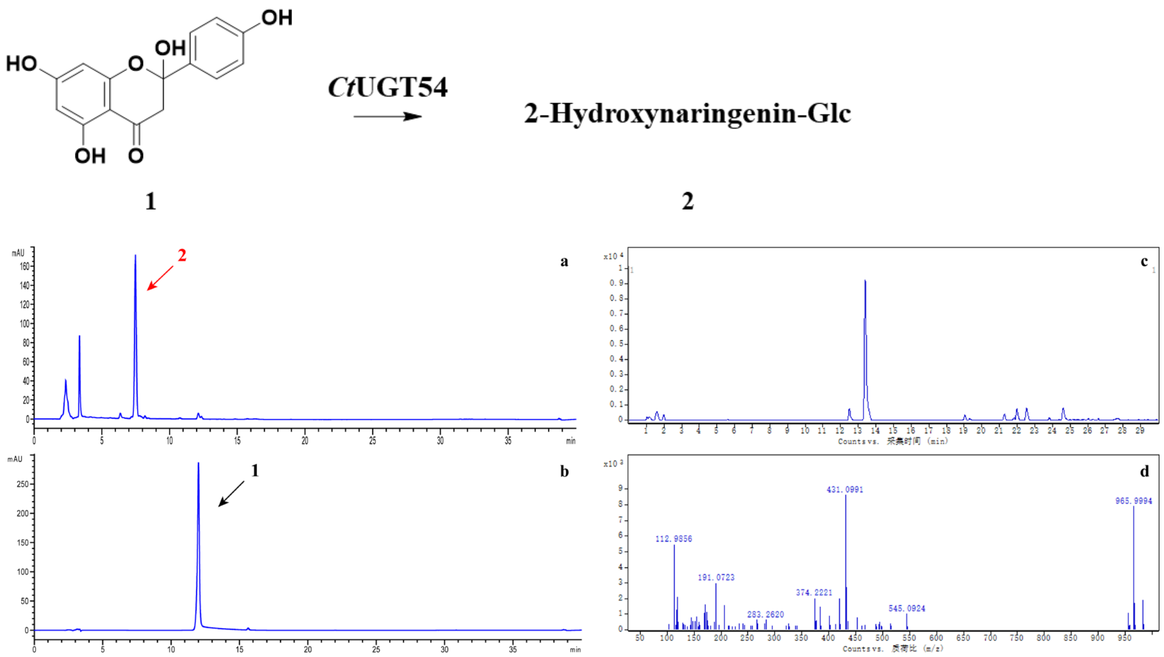

Figure S11. Results of the 2-Hydroxynaringenin glycosylation catalyzed by *Ct*UGT54.

a: Reaction result of *Ct*UGT54 catalyzed 2-Hydroxynaringenin (1) and UDP-Glc;

b: HPLC analysis of 2-Hydroxynaringenin;

c-d: LC-MS analysis of 2-Hydroxynaringenin glycosylation (Neg m/z 417)

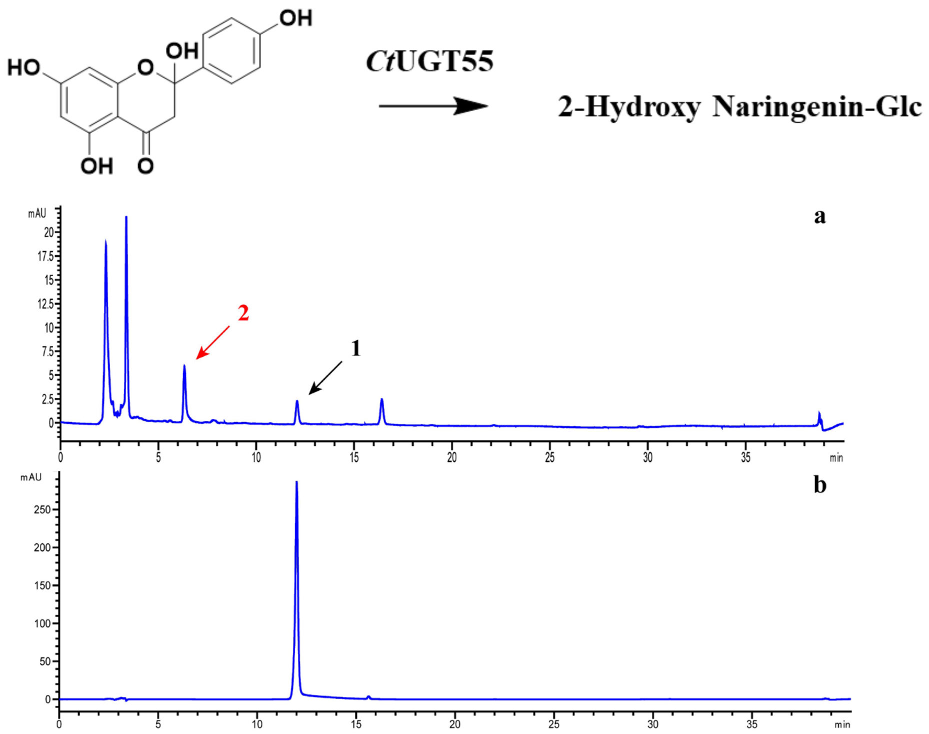

Figure S11. Results of 2-Hydroxynaringenin glycosylation reaction catalyzed by *Ct*UGT55.

a: HPLC result of *Ct*UGT55 reacted with 2-Hydroxynaringenin (1) and UDP-glucose;

b: HPLC analysis of 2-Hydroxynaringenin

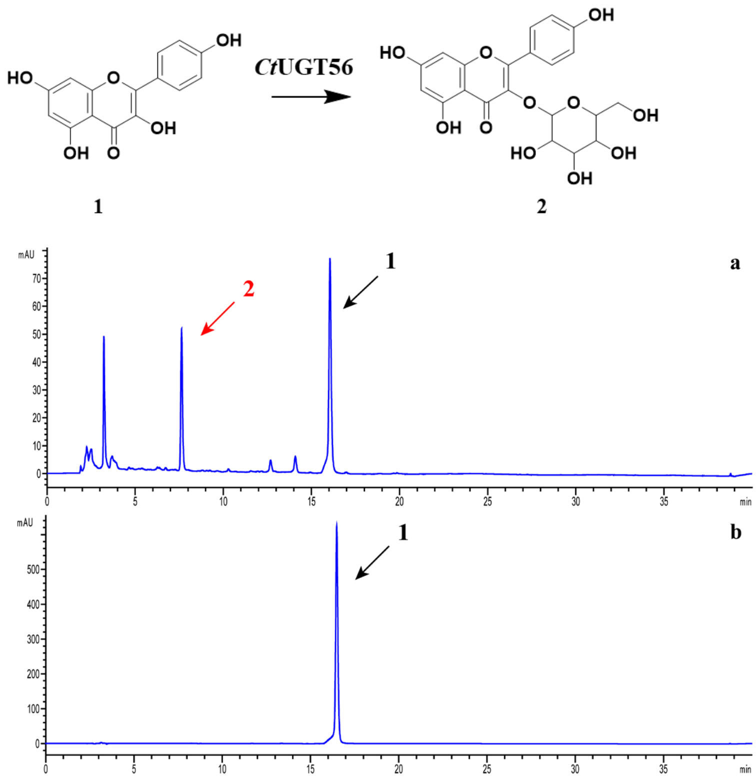

Figure S11. Results of Kaempferol glycosylation reaction catalyzed by *Ct*UGT56.

a: HPLC result of *Ct*UGT56 after reaction with Kaempferol (1) and UDP-glucose;

b: HPLC analysis of Kaempferol

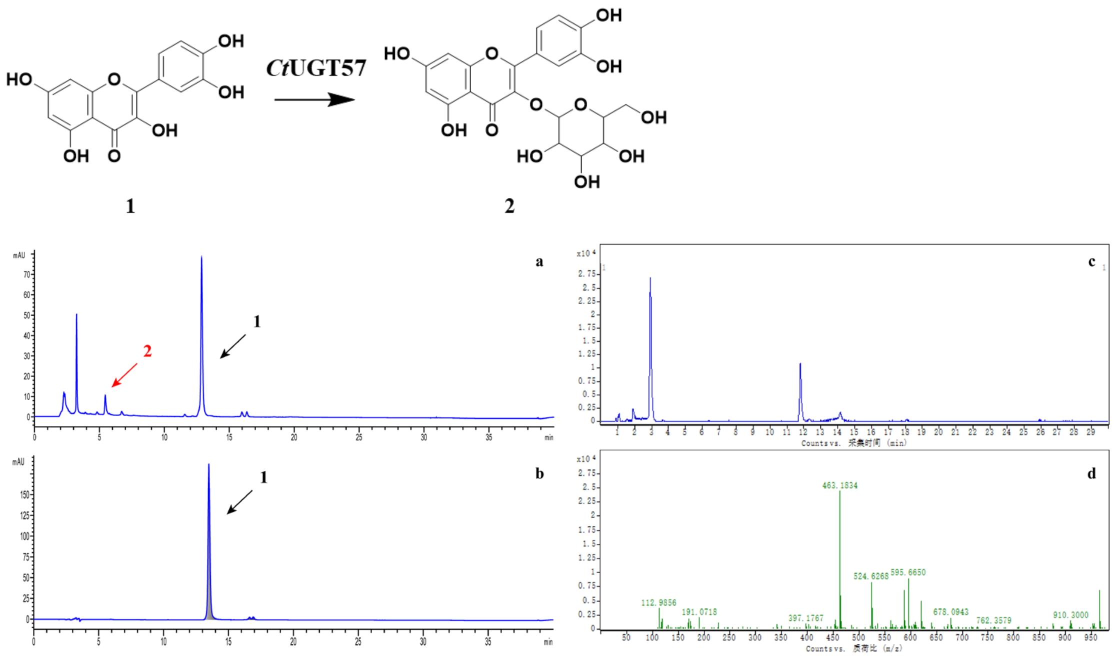

Figure S11. Results of the Quercetin glycosylation catalyzed by *Ct*UGT57.

a: Reaction result of *Ct*UGT57 catalyzed Quercetin (1);

b: HPLC analysis of Quercetin;

c-d: LC-MS analysis of Quercetin glycosylation (Neg m/z 463)
