# Supplementary material for: BSA-seq integrated with transcriptomics and metabolomics revealing the candidate genes associated with safflower colors and flavonoid glycosides biosynthesis
Source: Hortic Res. 2026 Mar 4;13(6):uhag068. doi: 10.1093/hr/uhag068 (PMC13253347; doi:10.1093/hr/uhag068)
Supplement: Web_Material_uhag068 [file web_material_uhag068.zip › Supplementary File.pdf]

Contents

Table S1. The sequencing depth of BSA analysis.
Figure S1. Morphological description of four different growing stages of yellow safflower.
Figure S2. Volcano plot of differential expressed genes among different accessions and growing stages.
Figure S3. Functional and Enrichment analysis of DEGs.
Figure S4. PCA and Venn diagram between different groups.
Figure S5. Volcano plot of differential expressed metabolites among different accessions and growing stages.
Figure S6. Analysis of targeted metabolites accumulation.
Table S2. Basic information of target *CtUGTs.*
Table S3. Sequence details of target *CtUGTs.*
Figure S7. Correlation analysis of differentially expressed *CtUGTs* with targeted metabolites.
Figure S8. The expression profiles of the 16 screened *CtUGTs.*
Figure S9. Phylogenetic analysis of target *CtUGTs.*
Figure S10. SDS-PAGE of the His-tagged *Ct*UGTs.
Figure S11. HPLC and LC-MS/MS analysis of glycosylation of *Ct*UGTs with different substrates.
Figure S12. Relative expression Levels of *CtUGT52* during different growing stages.
Figure S13. Comparison of wild-type (left) and *CtUGT52* overexpressed safflower (right).
Figure S14. Analysis of the key binding sites of *Ct*UGT52 with Phloretin (a), Kaempferol (b), Luteolin (c) and UDP-Glc.
Figure S15. Identified *Ct*UGTs of the different flavonoid glycosides biosynthesis in yellow safflower.
Table S4. Amplification primer sequences of *CtUGTs* recombinant plasmid.
Table S5. Primer sequences for site-directed mutagenesis and recombinant plasmid amplification.

Table S1. The sequencing depth of BSA analysis.

Chromosome	Mapped_Reads	Unmapped_Reads	Mean Depth	Coverage_Rate(1x)	Coverage_Rate(5x)	Coverage_Rate(10x)
*Ct*AH01	28948550	126240	43.82350436	0.993411693	0.989375291	0.98305224
*Ct*AH02	37248822	156989	50.94440091	0.99791496	0.996371872	0.99325474
*Ct*AH03	35533302	150209	44.0878565	0.964848942	0.94860019	0.937483661
*Ct*AH04	29734192	126390	43.75995377	0.974338499	0.963317227	0.955214219
*Ct*AH05	31569938	152656	43.2410419	0.989669719	0.984514549	0.977560011
*Ct*AH06	31130943	144127	44.62487386	0.986705871	0.980800525	0.975282466
*Ct*AH07	26377179	114545	48.84638	0.99362107	0.989906338	0.985823956
*Ct*AH08	30994100	161549	46.06381545	0.977694318	0.969725984	0.960722522
*Ct*AH09	27926170	119584	43.30891965	0.981427176	0.972279326	0.964879397
*Ct*AH10	34047853	141723	52.5526991	0.992409212	0.98781207	0.977805817
*Ct*AH11	24927606	108938	44.78087573	0.979755805	0.970034434	0.962434065
*Ct*AH12	38658572	226805	58.73450645	0.972856791	0.959594085	0.949910155

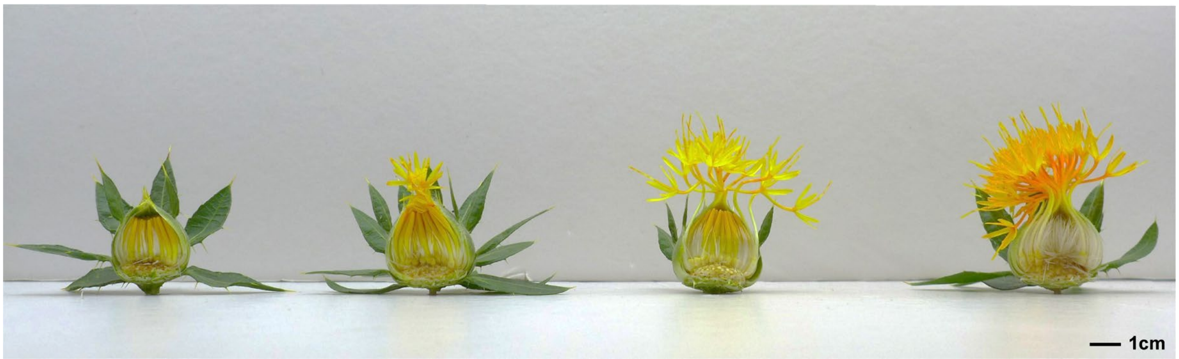

Figure S1. Morphological description of four different growing stages of yellow safflower (from left to right: Y_I - Y_IV).

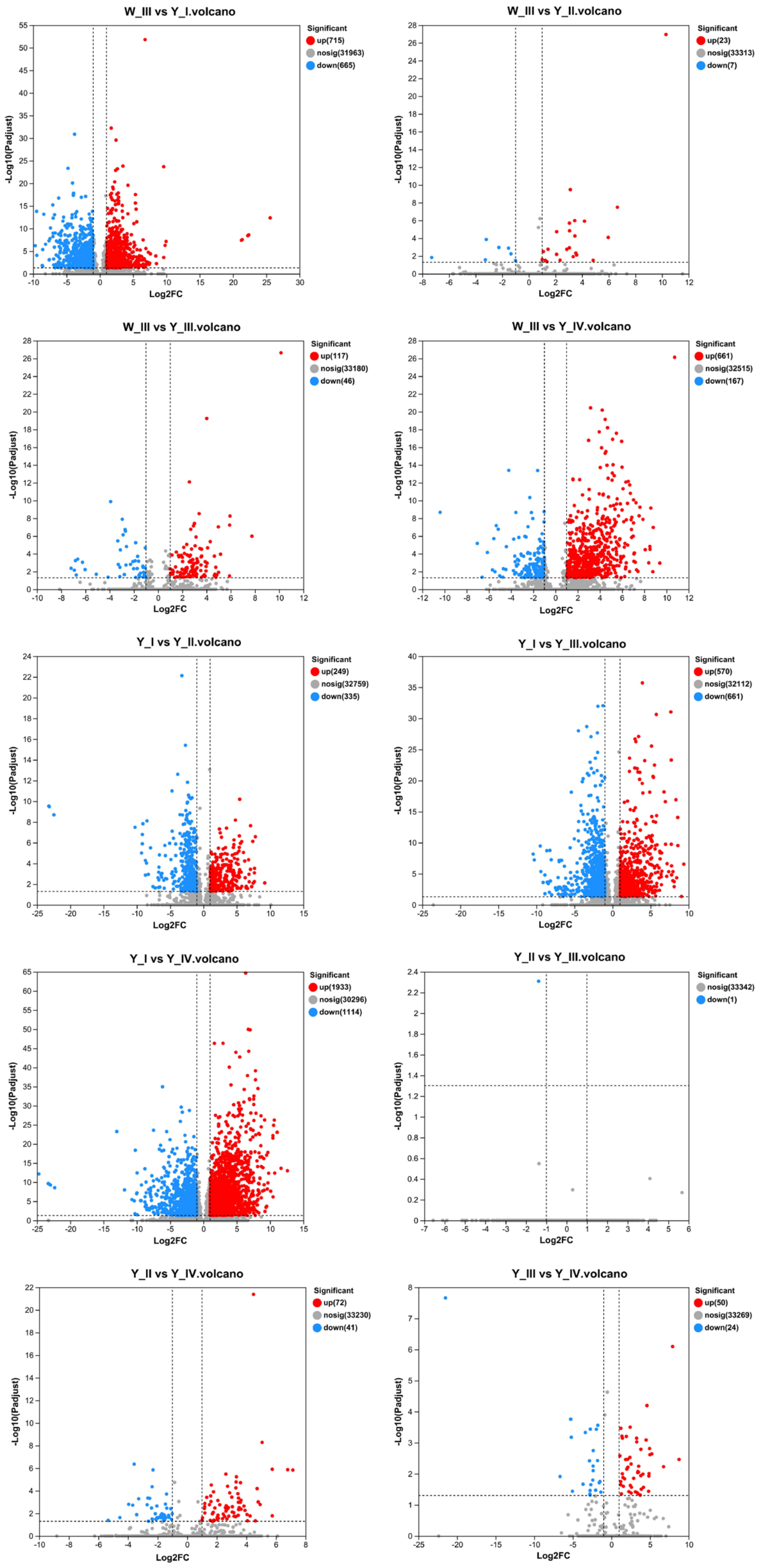

Figure S2. Volcano plot of differential expressed genes among different accessions and growing stages.

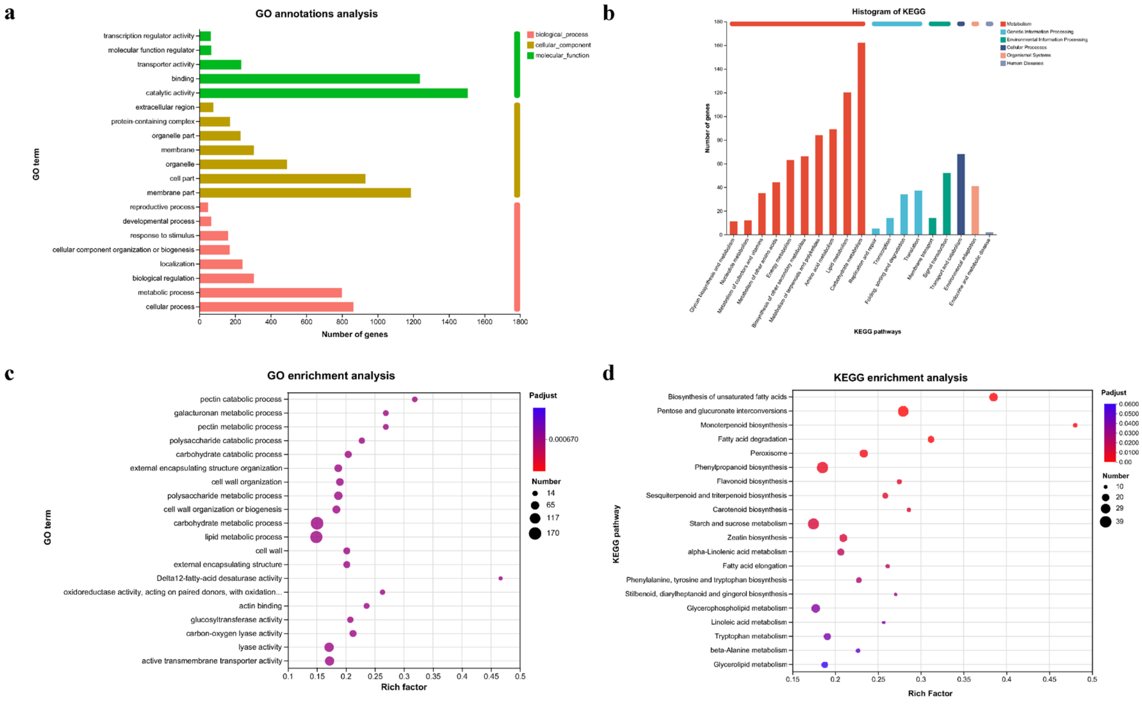

Figure S3. Functional and Enrichment analysis of DEGs.

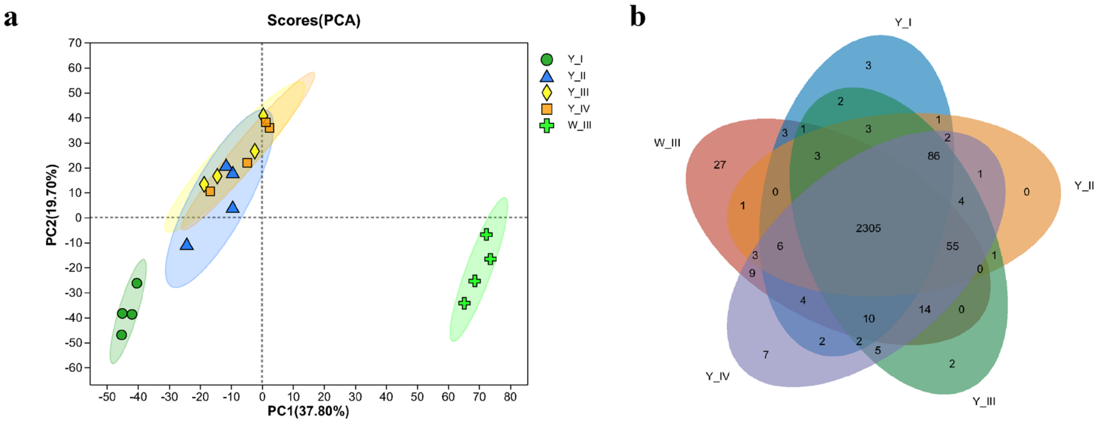

Figure S4. PCA and Venn diagram between different groups.

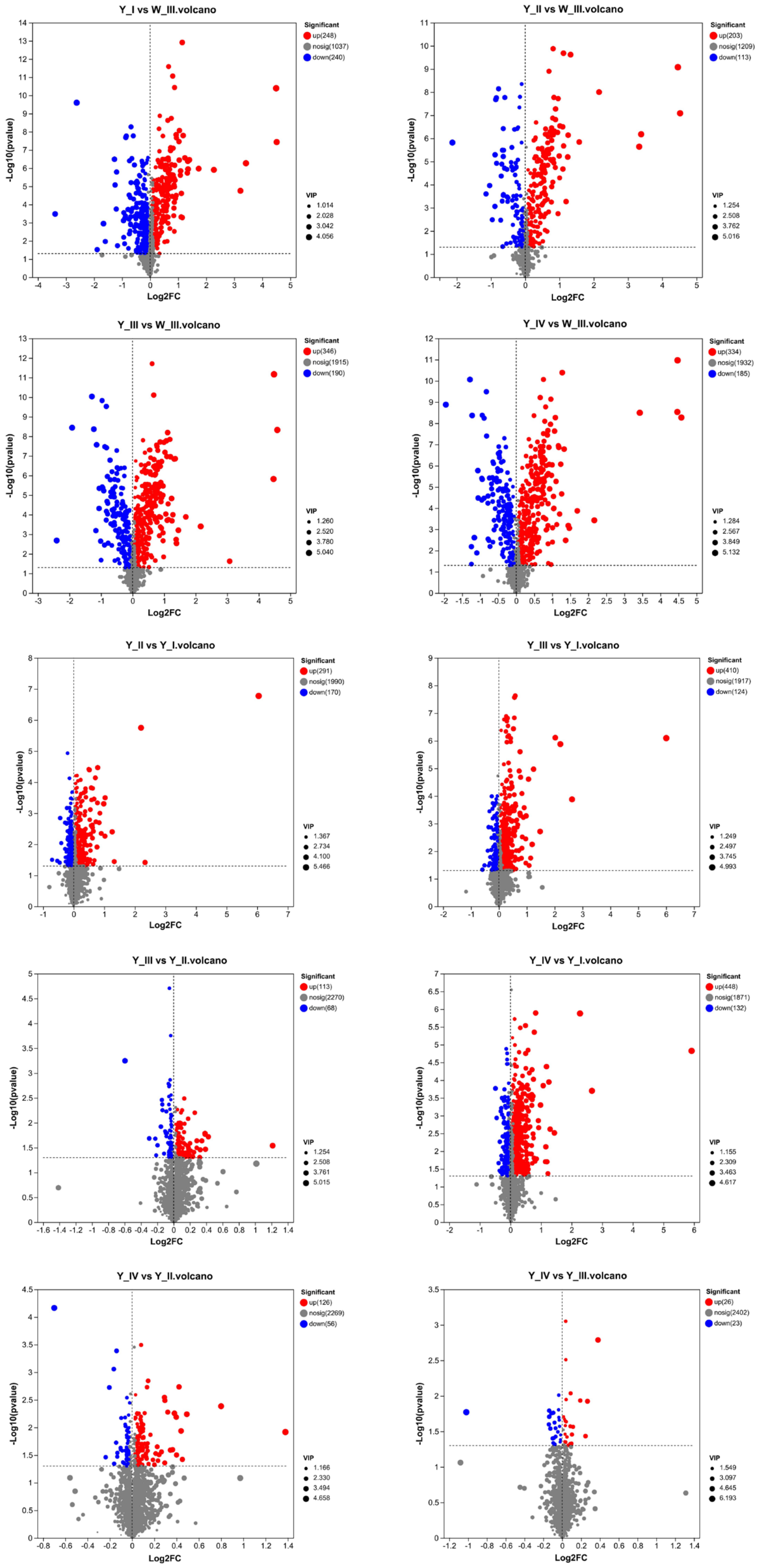

Figure S5. Volcano plot of differential expressed metabolites among different accessions and growing stages.

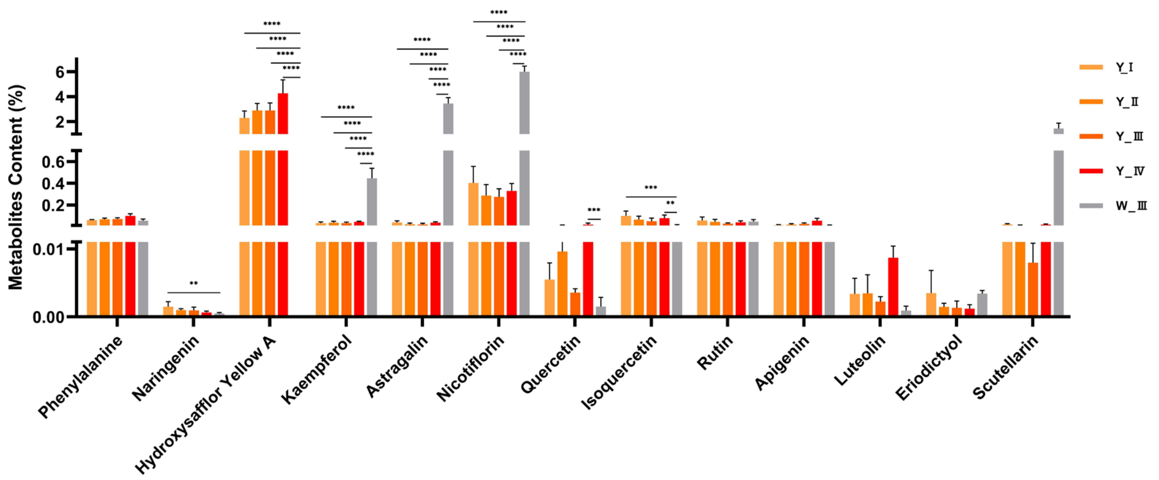

Figure S6. Analysis of targeted metabolites accumulation. (Mean ± SEM (n=3), $^{*}P < 0.05$, $^{**}P < 0.01$, $^{***}P < 0.001$, $^{****}P < 0.0001$)

Table S2. Basic information of target *CtUGTs*.

Name	
CtUGT50（*Ct*AH10G0075800）	Chrom: *Ct*AH10（Location: 12692989-12695463）<br>Molecular Weight: 50891.24 Daltons; 454 Amino Acids
CtUGT51（*Ct*AH03G0193500）	Chrom: *Ct*AH03（Location: 78922619-78925082）<br>Molecular Weight: 53484.66 Daltons; 486 Amino Acids
CtUGT52（*Ct*AH02G0117700）	Chrom: *Ct*AH02（Location: 20128343-20131008）<br>Molecular Weight: 51602.31 Daltons; 463 Amino Acids
CtUGT53（*Ct*AH06G0198500）	Chrom: *Ct*AH06（Location: 61643406-61649347）<br>Molecular Weight: 54038.73 Daltons; 471 Amino Acids
CtUGT54（*Ct*AH08G0149200）	Chrom: *Ct*AH08（Location: 18915565-18916974）<br>Molecular Weight: 52939.94 Daltons; 469 Amino Acids
CtUGT55（*Ct*AH01G0016500）	Chrom: *Ct*AH01（Location: 2035727-2053783）<br>Molecular Weight: 53229.51 Daltons; 491 Amino Acids
CtUGT56（*Ct*AH10G0065700）	Chrom: *Ct*AH10（Location: 10537371-10539956）<br>Molecular Weight: 50911.42 Daltons; 454 Amino Acids
CtUGT57（*Ct*AH09G0287800）	Chrom: *Ct*AH09（Location: 84723105-84728932）<br>Molecular Weight: 48188.96 Daltons; 429 Amino Acids

Table S3. Sequence details of target *CtUGTs*.

	Nucleic acid sequence (CDS)
CtUGT50	ATGAATCAAGTCGTAATGATACCGTTTCCAGGCAGAGGTCACATAAACCCTATGTTAAACCTCTGCAAATCACT<br>CTCCTCTCGAGTAAATCAATCCAATCGCACCACCGTCTTCACCGTCGTCGTCACCGAGGAGTGGCTCGGAATA<br>CTTAATCCGGATCTGAATCAACCTACCGTTCGTTTCGCTACCATTCCGAACGTCCTTCCATCGGAGCTCCATCGT<br>GGATCTGACATGATCGCCTTCCTCACCGCCATCTGTACCAAGATGAAGCGTCCTTTCGAGGAGGTTCTAGATCG<br>GATGGAGACGGAGATGGAGGTTCCGGTGGAGCTCATCATCGCCGATGCCGATATGCTTTGGCCGTTTGAGGTC<br>GCTAACGAGAGGAAGATTCCGGTAGCGGCGTACTGGCCGTTGCCTGCCTCGGCGTTTTCCATGATGCACCATG<br>CTGATCTACTTCGGTCGCACGGTCACATCGGCATTGATGTATCAGCAAGAGGAAAGGAGTGCATAGACTACATT<br>CCTGGATTATCCCCATTCACAGTAGCAGAAATTCCAATGATACTTCATGGTGGCATCTTCAAACACTTAACAACT<br>GATCTTTTCACCATAGCCCAAAAAGCAAATTGTCTTCTAATATCCACCCTTTACAACTTGGAATCCAAACCCATT<br>GATGCCCTCAAATCCAGACTAAAAATACCCATCTTCACATCAGGCCTAAACATACCACCATCCCCCCAAATCAA<br>CAACCCCATTAACAACAACCCTCCTCAACCCATTTACATAAACTGGTTAAACTCCAAACCCCCCAAAACAGTC<br>TTGTATATCTCTTTCGGCAGCTTTTTACCGATCCCCGATCACGAATTCGAAGAGATTGCCGCCGGTTTACTATCG<br>AGCGGAGTTAGCTTCTTATGGGTCGGCCGAGGGAAAACCGAGAATTTGAAGGAAACTTTATGTCGTGGTGGA<br>GATGGGAAAGCGATGGTGGTGGAGTGGTGTGATCAGCTGGCGGTTTTGTCGAACCCGGCGGTGGGTGGGTTT<br>TGGACGCATTGCGGGTGGAATTCCGTTAAGGAAGGATTGTTTTCGGGTGTGCCGATGCTGACTTTTCCGTTGTT<br>TCTCGATCAGCCGCTTAATGAGAAAATGATCGTTCGAGATTGGGGGGTTGGGAGGAAGGTGAGGGCGAACAG<br>GGCGGAGGGGTTTACGAGGGGTGAGATTGTGGAGGTTGTTAGAGGGTTTATGGATACGGAGAGTGTTGAGAG<br>GGTCGGGATGGTGGAGAGGGCGGAGAGGGTTCGGGAAATTTGTCGGGATGGTTTAACGGTGGACAGAGATCT<br>TGAGGTCTTCGTTAGGGAGTTTGTAACGGACCGAAAGAAA
CtUGT51	ATGCCGAATACAGTCGCAGAGCTGGTGTTCATCCCTGCACCCGCGGTCGGTCATATCACGTCGACGGTCGAGA<br>TCGCAAAACTACTCGTGAACCGAGATCAACGCCTCTCGATAACCATCCTTGTCATCAAGCTGCCTCTGAGATTG<br>GCTTCCGGCTCGGCTATCAAAGCCTACATCGATTCGTTGGCTAACAGGGCCTTGGACCGCGTATCCTTCGTCGA<br>ACTCCCGCAAGATGAAACCCCGCCATCTGGCGACCCAAAAGCTCCCATGACTTCTTTCGATGAATTCATCAAC<br>CGCCACTGCAAATACGTCAGAAACGTAGTGGCCGACATGATGAGTCAACCGGGTTCCGGCCGGGTGGTCGCC<br>GGGTTCGTCATCGATATGTTTTGCACCGGCATGATCGATGTGGCGAACGAGTTTAACATTCCAACTTACGTGTT<br>CTTTACTTCCAACGCCGCTTTTCTCGGATTTCAAATGCATATGTTGTCGCTCCGTGATGATCGGGACCGAGATCT<br>CGCCGAATCGACCGACTCGGATGCTACGATACCCGTTCCGAGTTTCGTCAATCCGGTGCCAACGAAAGTATTTC<br>CGTCAATGGTGAAGTCAGAAGGGCTGGATTTTGTTACGAGGATAGTCGGGAAAATGAGAGAGGCGAAGGCGA<br>TCGTGGTTAATACGTTCCTGGAATTGGAAACGCACGCGATCGAGTCGTTGTCTTGTGATAGCAGCGGACCGTTT<br>GTGTATCCGGTGGGGCCGCTACTCAATCTTGAAGATGGTGCCGGAAAACAGTTGGACGATGAAGTGAAGAGG<br>TGGTTGGACGGTCAACCGCCGTCCTCGGTGGTGTTCTTGTGTTTTGGGAGTATGGGAAGTTTCGAGGAGGTCC<br>AAGTGAAGGAGATAGCGTATGCTCTCGAGCGGAGCGGCCACCGTTTCTTGTGGTCCCTACGTCGACCTCCGTT<br>GGCAGAAAAAGGATTTGGGAATCCAAGTGATTACGAGGATCCACGAGTGGTGTTGCCGGAAGGATTCATGGA<br>CCGCACTGTCGGAATCGGAAAAGTGATCGGGTGGGCTCCACAGGTGGCGTTGCTGGCTCACCGTGCTATCGG<br>GGGGTTTGTGTCCCACTGTGGATGGAACTCCGTGTTGGAGAGTTTATGGTTCGGTGTACCGATTGCGGCATGGC<br>CAATGTACGCCGAGCAACAATTGAATGCATTTGCAATGGTGGTGGAGCTGGGATTGGCGGTGGAGATTAAGAT<br>GGATTACAAGAAAGATATGTTTAATCCTAAGGCCGAGATTGAGTTAGTGACGGCGGATGAGATCGAGGGCGGC<br>ATAAGACGGCTGATGGCGGATGAGACCATCAGAACACGAGCAAAAGAGATGAGCGAAAAGAGCAGAGCAGC<br>GGTGGCGGAAGGCGGTTCTTCGTATGCTTCTGTTGGATCTCTTATCGAGGATTTTATGAGAAACATCTCATGA

CtUGT52	ATGGAGAAAAGCTACAAAGGTCATGTTTTGGTGTTACCTTATCCAAGCCAAGGCCACATCAACCCCCTCCTCC<br>AATTCGCCAAAAGGCTCGCATCCAAAGGCCTCAAAGCCACCATCGCCACCACCCACTACACCCTCTCCTCCAT<br>CTCCGCCCCGTCCGTCGCGGTCGAACCCATCTCCGACGGCTTCGACCACGGCGGCTACGCCCAGGCCCAATCC<br>GAAAAGCTCTTCCTCGACTCCTTCAAATCCAACGGCTCGCGTACGCTCGACCAGCTCATCAAGAACCACCGGA<br>CCACCGACCACCCGATCACTTGCATCGTCTACGATTCCTTTCTGCCTTGGGCCCTTGACGTGGCCAAGGAGAAT<br>GGGATCCTCGGGGGCCCGTTTTTCACGAACTCGGCCGCGGTGTCGGCCATTTTCAGCCGGATATACGCAGGGA<br>CGTTGAGGTTGCCGGTGAGAATGGAGGATTGTCCGGTGGTGCTGCCCGGGATTCCGCCGTTGGATTTGGAAGA<br>CTTGCCAAGCTTTTTGAATGCGCCGGAAAGTTATCCGGCGTACTTGGAGATGAAGCTGAATCAGTTTTCGAATC<br>TGGAGAAGGCTGATTGGGTTTTCAGCAATACTTTCCAAGCATTGGAAGATGAGGTGGTGCAAGGATTGGAGGA<br>GCAATGGCCAGCAAAACTAATAGGTCCAATGGTGCCATCAGCCTACTTAGATGAAAGAATTGAAGGTGACAAA<br>GGGTATGGTGCAAGTCTATGGAAACCACTTGGCCATGAGTGCACCAAATGGCTTGAATCCAAGCCCCAAAACT<br>CAGTTATCTACATTTCCTTTGGGAGCATGGTGTCCTTAAGCCAGCAAGAAATGGAAGAAATAGCATGGGGTTTA<br>CAAAAAAGCGGGTTTGACTTTCTTTGGGTAGTCAAAGATACAGAGAGACACAAGTTGCCAAAAGGGTTTCTT<br>GATTTCATCACACAAAATCAAGAAAAGGGTATGATAGTAAATTGGTGCAACCAATTAGAGATACTAGCTCGTAA<br>ATCAGTGGGTTGTTTCGTGACACACTGTGGGTGGAACTCGACACTGGAAGGGTTGAGTTTGGGTGTGCCGATG<br>GTCGGGATCCCAAAGTGGGCTGACCAATTGACTGATGCCAAGTTTATAAAAGATGTGTGGTGTGTGGGTAGTA<br>GGGTCAAGGTGGATAGTGAAGTAGGGATTGTGAAAAGGGAAAATGTTATTATGTGTTTGAATGAAGTGATGAA<br>AGAAGGAAAGAGGGGTTTAGAGATCAAGAAAAATGTGGGGAAATGGAGGGAGATGGCTAAAGAGGCAATTA<br>GTGAAGGTGGGAGCTCAGATAAAGCAATTGATGAGTTTGTAGTGGCATTGAAGACATTTGCAAGAAAAAATAA<br>C
CtUGT53	ATGGTATATGAAGTCAATGAATACATGGATAAGAAAGAACATATTGCGATTTTTACCACAGCTAGCCTTC<br>CATGGATGACTGGAACCTCTGTTAATCCTCTATTCCGTGCAGCGTATCTTGCCAAAGATGGACATAGAAA<br>GGTTACTTTGGTCATTCCTTGGCTATCGAAGAGCGATCAAGAATATCTATATCCCAACAAAATAACATTT<br>AATTCGCCCAAGGAACAAGAGAAATATGTCCATGAGTGGATTGAGCAAAGGACTGAGTTTTTGCCTAG<br>TTTCAATATACGTTTTTATCCAGGGAAGTTTTCTAGAAGTAAAAGAAGCATTCTTGCTCTTGGGGATATA<br>ACGGAAAGCATTCCGGATGAAGAATCTGATATTGCCGTCCTTGAGGAACCCGAACATCTAACGTGGTAC<br>CACCATGGTAAAAGATGGAAGATCAAATTCCGCCTTGTTATAGGAATTGTTCACACGAACTATTTGGAG<br>TACGTCAAAAGAGAGAAAAATGGACGTGCCTATGCCTTTCTTCTCAAGTACATGAATAATTGGGTGGTC<br>GATATATACTGTCATAAGGTAATACGATTATCTGGAGCAACACAAGAACTCCCAAGATCCGTTATTTGCA<br>ATGTTCATGGCGTTAATCCTAAGTTTCTGGAAATCGGGATGAAAAAGAGGGAAGAACAGAAGCTCGGA<br>AAGCAGGCGTTTACGAAAGGTGCATACTTTATTGGGAAAATGGTGTGGAGCAAAGGCTACACGGAGCT<br>GCTTAAACTTCTTCGTGATCACCAAAAGGAACTTGAAGGACTTGAGGTTGATTTATTTGGTACCGGTGA<br>GGATTCTGCTGAAGTACAAGAAGCTGCAGAAAAGTTGAATTTAACCATCAGGGTTAATCCAGGACGCG<br>ATCACGCAGACCCTTTATTTCACGATTACAAAGTGTTCCTGAATCCAAGCACCACAGATGTGCTCTGCA<br>CAACCACCGCCGAAGCTCTTGCAATGGGCAAAATCGTCCTTTGCGCAGATCACGTCTCTAACGAGTTCT<br>TCAAACAGTTTGCCAATTGCCGAACCTTCAAGGACGGAGAGAGTTTCGTTAATGTCACCCGCCAATCG<br>TTGACCGAACAGCCGGCCCCACTGACCAATTCGGCAATGCACGAGCTTTCATGGGATGCTGCAACCTC<br>GAGGTTTCTAAAAGCGGCTGAACTCAATAAGACTCCCGAGAAGAAACTAACGAAATCACATTCCAAGA<br>GCTTTTTGTCATCGTCATTGAGTTTTCAACGGAATCTCGAGGACGCGTCTGCGTTTATGCATTTCGTTGG<br>AACCGGTTTCGTGAGTCCAGTGCCGAACGAGCAACAATGTGAAGAGCTCGGGCTGAACGTCCCAAGT<br>AAAAGATTCGGATATCCACGTTCAAAACACGCATCT

CtUGT54	ATGGAGGAGAAAGTAGAGGTGTTTTTCATCCCATCGCCACTTATGGGACATGTTGGCCAGATGGTTCAG<br>CTGGCCAACCTCATGGTCACCCGGTTTCACCATCTCACCATCACCATACTCGTCATGCACCTCCCCACC<br>GACCCCATCGGCACCGATTACACCAACTCCCTCGACGACCACCACGATCGAATCAAATTCATCCAATTC<br>CCTCCGATGGATCCCGACTCTTTTCCGGATTGCCCGACTGTCGGTTTCATGGCCGATGCCATCATTGAAC<br>GCCATAAGCCCATCGTCGGAGAACTCGTGGCTGCTCGCTTCAACGGGTCCAATCGCACCCCTCGACTC<br>GGCGCCTTGGTCGTTGACATGTTTTGCACGCCGATGATCGATGTCGGCAAGGAATTCGGTGTCCCCACC<br>TACGTGTTCTTCACCTCAAATGCGGCTTTTCTCGGGATTATGTTATATTTCCAGACCCTTGAAGACGAAC<br>ACGGCCAGGAGACACCCGAATTGGCGAATCCGGGTCCTCCGTTGATCATCCCGAGCTATGCTGAACCG<br>GTTCCACCAAGTGTCTTGCCCTATGTGCTTTCGGACCAGGACACTTGGTACAAAAGGTTTATTCGTTATA<br>CCCGAAAATACAGAGAAGCCAAGGGTATAATCGTAAACACGTTTCGAGAGTTAGAGCCTCATGCGCTC<br>CTTTCCTACGACGATAAAACACCACCTGTTTACACGGTGGGTCCCATGCTAAAACCCGAAAAGCCTACA<br>CCAAACAACGAGTTGCTTCAGTGGTTGGATGGTCAACCGAAGTCATCGGTCTTGCTCCTATGCTTCGGA<br>TCCCGGGGGTGGTTCGAGGTGGACCAAGTGAAACAAATAGCGATTGCTATAGAAAGGAGTGGATACAG<br>GTTCGTATGGTCCCTACGCCAACCTCCAACCGAGAACCAAAAAGGGTTCCCAAGGGAGTACACAGACT<br>ACAACGAAGTCCTGCCAGATGGGTTTCTTGACCGTACAGCTGGAAAGGGCAAAGTGGTTGGGTGGGT<br>CCCGCAAACGGCATTATTGGCTCATGTGGCAGTTGGTGGGTTCGTATCCCACTGTGGGTGGAACTCCAT<br>ACTGGAGAGCCTCTGGTACGGAGTTCCAATTGCCACATGGCCAATATACGCGGAGCAACAGTTAGATGC<br>GCATCAATTGGTTAAAGAACTGGGTTTGGCAGTCGAAATCTCGTTGGATTATAACCAATTAAACAAGAA<br>TCAAAGGTTGGTGTTGGCTGAAGAGATTGAAAAGGGAATACGGCAGGTGATGGATAGCAATAGTGAGG<br>TTCGAGCAAAGGTGGCACAAATGAAAGCAAAGAGCCGAATGGCACTCGAAGAAGGTGGTTCATCGAT<br>TAACAGTTTGAAAGACCTTGTAGACGATTTCATG
CtUGT55	ATGCCGACCGCCACCGTCCGCCACCACCAGCCACCGCATATCGCCCTCTTCTCGAGCGCCGGAATGGG<br>CCATTTGACCCCACTCCTCCGCGTTGCTTCCATGCTCGCCTCCCGCAGCTGCCACGTAACTCTCGTCAC<br>CGCCGAACCCGCCGTCTCCGCCGCGGAAACCGCCCACATCACCGCCTTCCTGGCGGCGTATCCCGCCG<br>TCAACCGCCTGCCCTTCCGAACCCTTCCGTTCACACCGCCAGCCACCGCTGACCCTTTCTTCGTCCAAT<br>TCGAAGCCATCAACCGCTCCGTCCACCTCCTCGCCCCGACCTTGTCCTCCGCATCGCCGCCGGTCTCCG<br>CCGTCTTCTCCGACATGGCGTCGGCTGCCGGGGTCCGCCGGGTGGCCGACGAGCTTCGGGTCCCGATC<br>TACATCGTCTCGACCACCTCCGCCAGGTTCACGGCCCTGGTGGCGAGCATCCCGGCCCTGATCGGGGC<br>CGGAAGCTCCATAACAGCGACGGCGGAGGGAGCTTCCTCCGCCGTCTTTGGGATCCCTGGTCTGGATC<br>CGTTTGAGATCTCGGCGCTTCCGCCGGCGCTGTTCGTACCGGATAACCTTTTCACGAAGACTTTGGCTG<br>CGAACGCGCTTGCGATGAGAAAGGCGAAAGGCATTTTGACGAACACGTTCACTACATTCGAACCGGA<br>AACGATTGCAGCGGTTAACGGCGGCAAATCTTTACCGGATTTCCCGCCCCGGACCGCCCTGTCGCAAC<br>CCCAAATCGTCGAGCTCCGAAACGGGCTGGAGGAGAGCGGGCGGAGCTTCTTGTGGGTCTTCAAGTC<br>CACGGTCGTCGATAGAGACGACACCGGATCCGACCTCGGGGAATTGCTCGGCGGGGATCCGACGAAA<br>CCGTCGAACGGGATGGTGGTGAAAGGGTGGGTGAACCAAGAGGCGATCCTGTCGCACCCCGCGATCG<br>GGTGCTTCGTGAGCCACTGCGGGTGGAACTCGGCAGTGGAGGCCGCGGCGGCCGGGGTGCCGGTGGT<br>GGCGTGGCCGCTGGCGGGGGACCAGAAGGTGAACGCGGAGGTCGTGGCGGGGGCGGGGTTGGCGCG<br>GTGGGAGAAGGGGTGGGGGTGGATGGGGGAGAGATTGGTGAAG

CtUGT56	ATGGCGGACTCCATCACCTCCTCCCAGTCCCATGTGGTGGCGATACCATACCCCGGCAGAGGCCACATC<br>AACCCAATGCTCAACCTCTGCAACCTCATGTCCCTCCGCCGCCCTTCCGACCTCCTCATCACCGTCGTC<br>GTCACCGAAGAATGGCTCGGATTCATCGGATCCGACCCGAAACCGACAAACGTCCGCTTCGCCACCAT<br>CCCTAACGTCATCCCGTCGGAGCTCGACCGCGCCTCCGACTTCGCCGGCTTCATCAAATCCATTCACAC<br>AAAACTAGTAGACCCGGTCGAGAGATTACTCCGCCGGATGGAAATTCCGGCGACCGTAATCATATACGA<br>TACCTACCTCATGTGGATCATAGATCTCGGAAAACGGTTGAACATTCCGGTGGCTTCCTTCTTCACGATG<br>TCGGCCACGGTGTTCTCCATGTGTTATCATCACGATCTCCTCCTCCAAAACGGCCATGTCGGAGATGATT<br>ATTTCTCAGAAAAAGGTGAGGAAGTGATCGATTACATACCTGGAGTGCCTCCCATGCGCGTGGCTGATC<br>TCGTGACAGGCTTCAATGGCAAAGGAAAAGAGGTTTTTCCGTTAGCTCTGCAAGCTATTTTAATGGCGG<br>ACAAAGCTCGGTTTCTGCTTTTCGTGTCGGTTTACGAGTTGGAAGATAAAGTGATCGATGCCTTAAAAT<br>CGGAGCTTTCGGTGCCCGTTTACGCTATTGGGCCGTCCATACCCTACTACTTCAATGTCCAAAATGACCA<br>AAATACCCCTGACTATCTGGAATGGTTAGACCGTCAGCCGGAGGCCTCGGTGTTGTACATCTCGCAAGG<br>GAGTTTTCTCTCGGTCTCGAATGCGCAGTTGGAGGAGATCGTCGCGGGCGTGCATGAGAGCGGTGTAC<br>GGTACGTGTGGGTTGCACGTGGCGAGACGTCTCGGTTTGGACGCGAAAATGACGAAAGTGGGCTTGT<br>CATACCTTGGTGTGACCAATTACGGGTGCTGTGTCATGGTTCGGTAGGGGGATTTTGGTCACACTGCGG<br>GTGGAATTCGACGAAAGAAGGTGCGTATGCGGGGGTGCCGATGCTCACGTTTCCCATATCTATCGATCA<br>AGTTCCGAACAGTAAGATGATCGTTGAAGATTGGAAAACGGGAAGGAGGGTGAGCGTTGACGAGGGT<br>GTTTTGGTCACTCGAGACGAAATCGCAAAACTCATAAAGGGTTTCATGGACGAGGAGAGCGAAGAAG<br>GAAAAGAGATGCGCAAAAGGGCAAGAGAAATCAAGAAAATCTGTCGACACGCGACCGATGAAGGAG<br>GGTCCGCTCAGAAAGATATCGATTTATTCATCGATGACATTTTGATCAGTCGAAACAAT
CtUGT57	ATGGAAGAAGGTGGAAGACGACTGGTGGTCCTAACTTCATCCCCATTTCATGGCCACATGACTCCAACT<br>CTCCAGCTAGCCACCGCCCTTCATGCCAAAGGCTTCTCCATAGCCATAGCTCACTCCACCTTAAACCCT<br>CCTCACCCATCCAATCACTTCACCTTCCTCCCGCTTTCCGACAACTTATCCGCCATCGATGCCTCCTCAA<br>GCTTCACCGGTTTCGTCCAAACCCTCAACGCCAACTGCCGACCATCATTCCGTCAACACTTGGTTCGGT<br>TGATCGCCCAAGGAGATTATGAATCGATCGTTGTCATCTATGATTTCCTTATGTTTTTTGCAGGAGGGGTT<br>GCCGTCGATCTGAACCTTGGTTCGATCATCTTTCGTAGTAACAGTGCTACGTACTTTACGGCTTTTCTTG<br>CCCGTCGGCAGCTGATTCAAAAAGGCCGGTACAAAGATCTGCCATTTTCGAAATCGCCTATCGAAGATT<br>GGCACCAACTGCTTGCTATCTTCAGCCAACAAAGCAACCCCTCCGCAGTTATCTGGAACACTCTCGAAT<br>TTCTCGAACACGAAGCCTTATCCCAAATCCACCAGCACTACAAGGCTCCGGTCTTCGCAGTCGGACCTC<br>TTCACAAGATAACGCCAAGTCCACCTACTAGTTTTCTCGAAGAGGACACCCGCTGCATAGCGTGGCTAG<br>ATAAACAAGCCCCCAAATCCGTGGTTTACGTAAGCTTTGGAAGTCTAGTTAGCGTAGATGCAAAAGTGT<br>TGGTCGAGATGGCATGGGGTCTGGCCAAAAGTAAGCAGCCGTTCCTATGGGCGGTTAGGCCTGGTTCG<br>GTTAGGGACTCCGAATGGAGCGAGTTCTTGCCGGATGGTTTTTGGGAAGAAACAAGCGGACGAGGTCT<br>AGTTGTGAAATGGGCGCCCCAAAAGGAAGTTTTGGCGCATTCCGCGGTCGGTGGGTTTTGGAGTCATT<br>GTGGTTGGAATTCGACGTTGGAGGGTATTTCGGAAGGGGTTCCGTTGATATGCCAACCGATTAACGTAG<br>ACCAAGGGGTGAACGCGCGATACGCGAGTTACGTGTGGAAGATAGGGGTGGAGTTGGAGGTTTTGGA<br>GAGAGGGGAGATGGAAAGCATGATCAAAAGAGTTATGGTGGATGAAGAAGGAAGGAGATGAGATTG<br>AAAGTAGCTAAAATGAAGGAAATGGTTAAAGATGCAGTGAAAAATGGAGGTTCTTCCCATGATTCATTG<br>GAGAGTTTAGTG

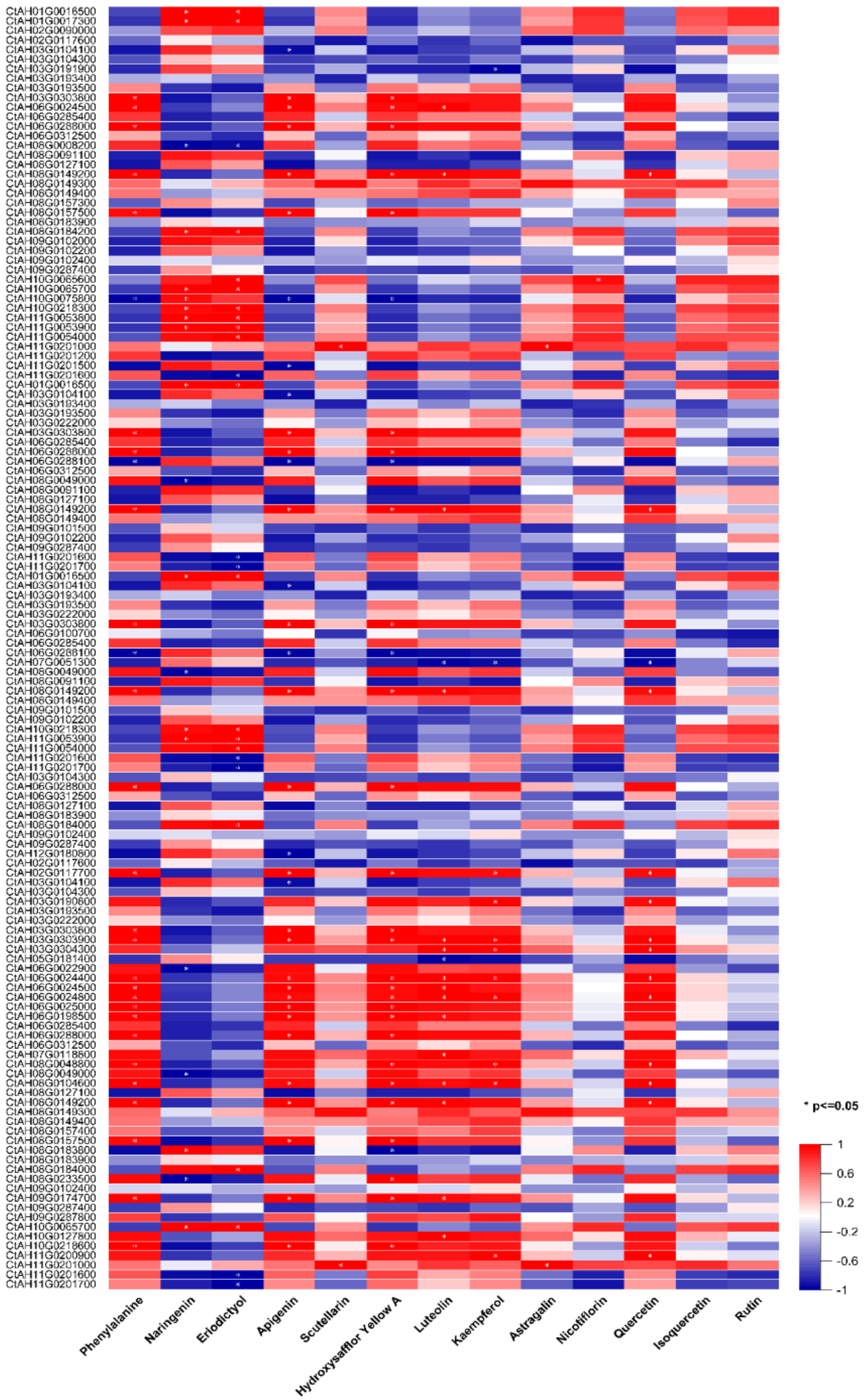

Figure S7. Correlation analysis of differentially expressed *CtUGTs* with targeted metabolites. (* $P < 0.05$)

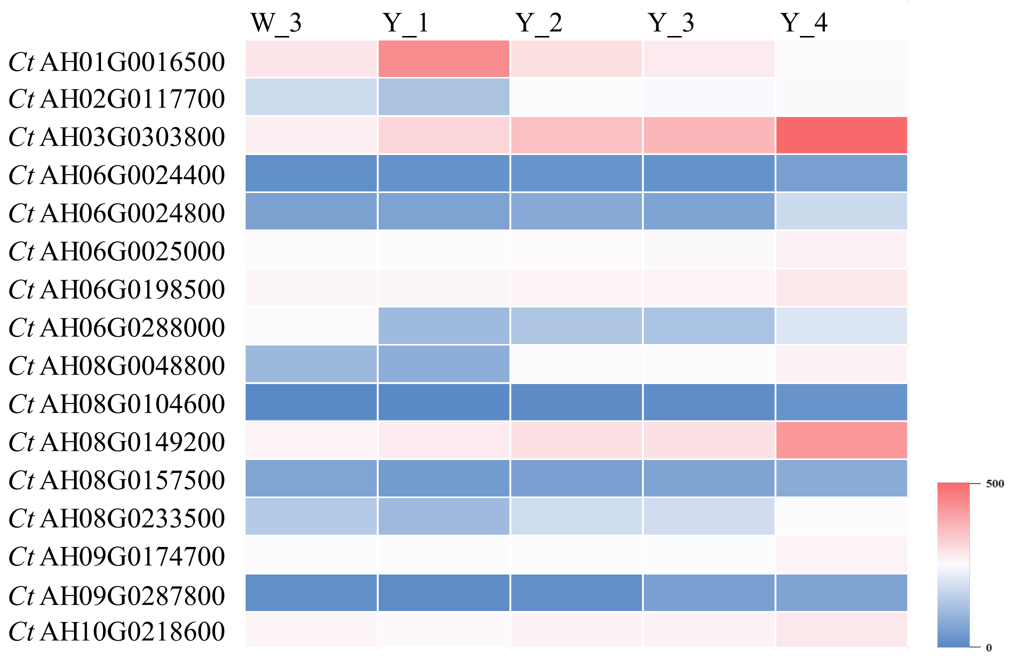

Figure S8. The expression profiles of the 16 screened *CtUGTs*.

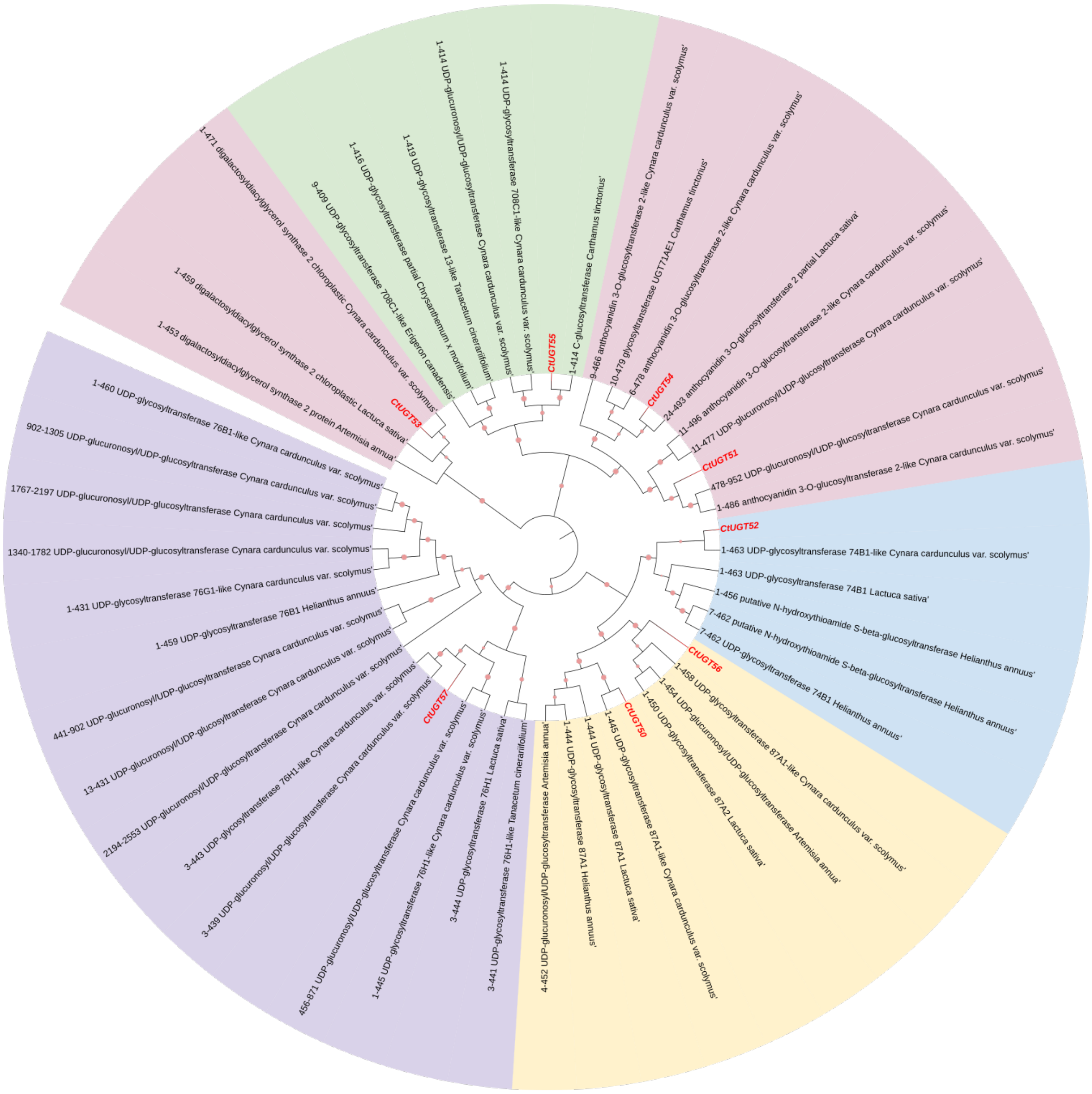

Figure S9. Phylogenetic analysis of target *CtUGTs*.

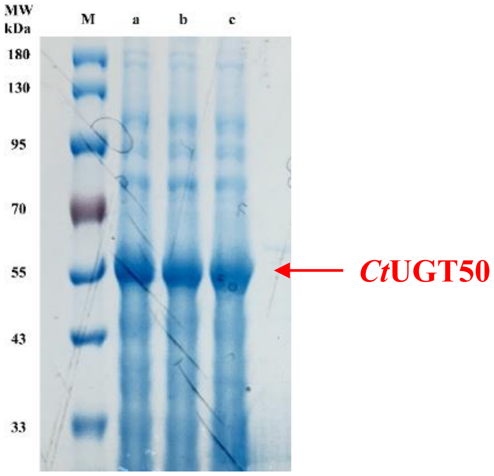

Figure S10. SDS-PAGE of the His-tagged *Ct*UGT50.

M: Marker; a-c: IPTG-induced protein expression bacterial supernatant

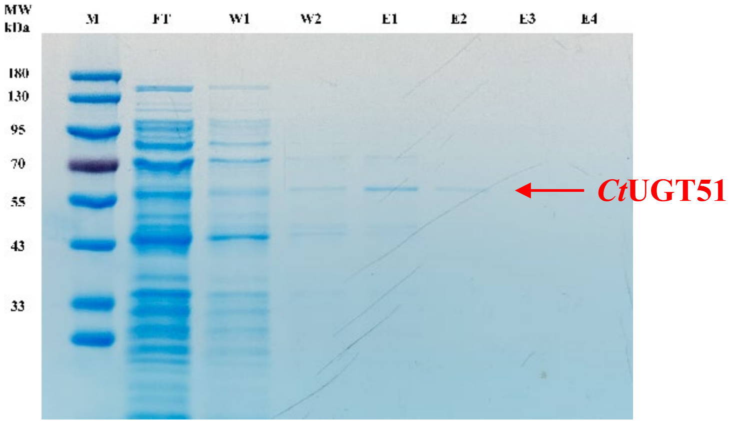

Figure S10. SDS-PAGE of the His-tagged *Ct*UGT51.

M: Marker; FT: Flow Through; W1-W2: Wash Solution 1-2; E1-E4: Elution Solution 1-4

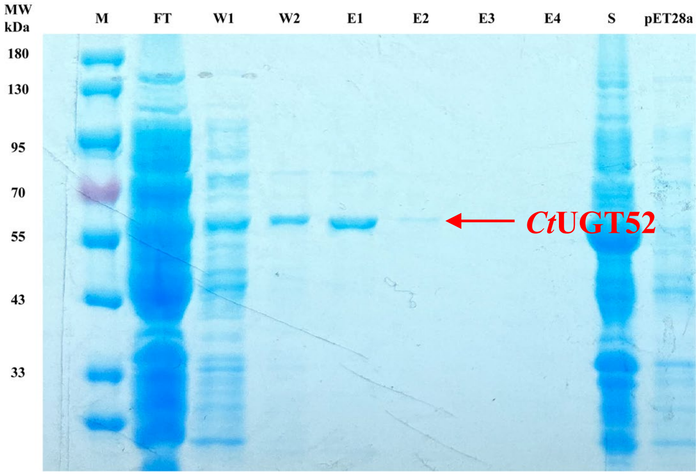

Figure S10. SDS-PAGE of the His-tagged *Ct*UGT52.

M: Marker; FT: Flow Through; W1-W2: Wash Solution 1-2; E1-E4: Elution Solution 1-4

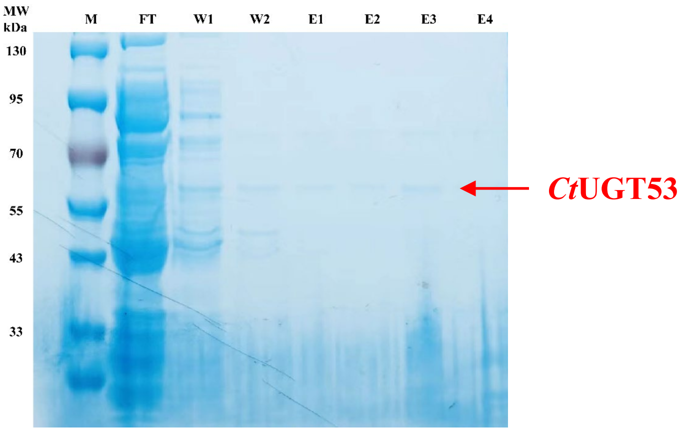

Figure S10. SDS-PAGE of the His-tagged *Ct*UGT53.

M: Marker; FT: Flow Through; W1-W2: Wash Solution 1-2; E1-E4: Elution Solution 1-4

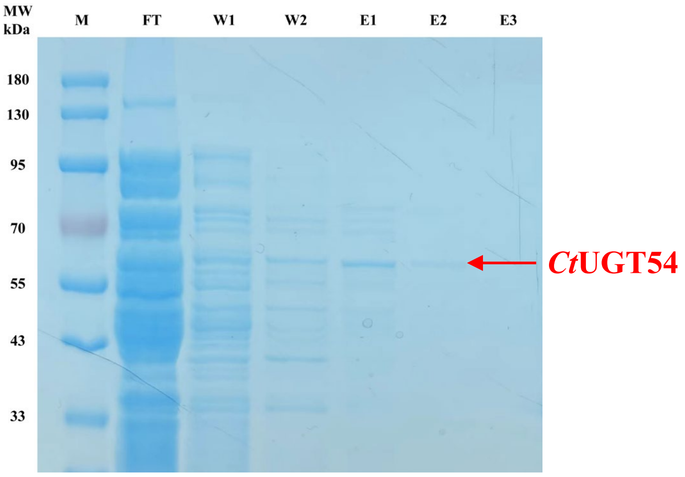

Figure S10. SDS-PAGE of the His-tagged *Ct*UGT54.

M: Marker; FT: Flow Through; W1-W2: Wash Solution 1-2; E1-E3: Elution Solution 1-3

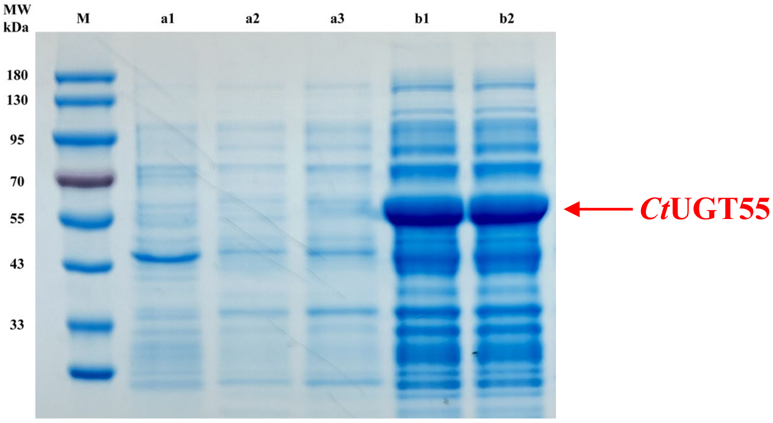

Figure S10. SDS-PAGE of the His-tagged *Ct*UGT55.

M: Marker; a1-a3: expression bacterial liquid of blank vector; b1-b2: expression bacterial liquid of *Ct*UGT55

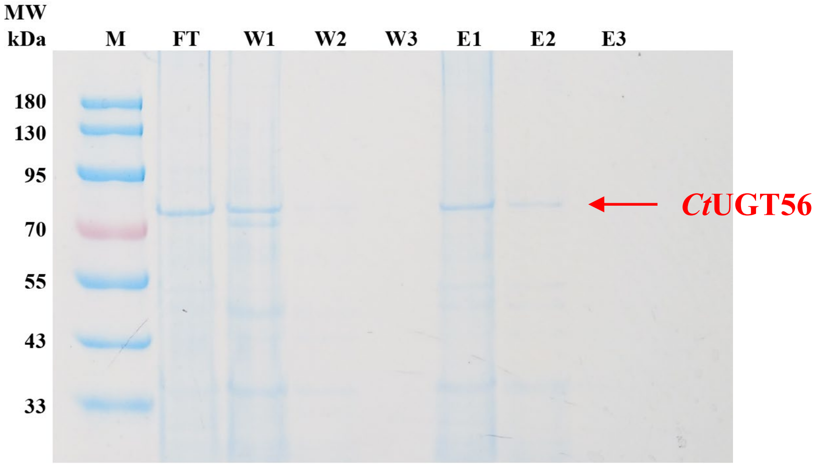

Figure S10. SDS-PAGE of the His-tagged *Ct*UGT56.

M: Marker; FT: Flow Through; W1-W3: Wash Solution 1-2; E1-E3: Elution Solution 1-3

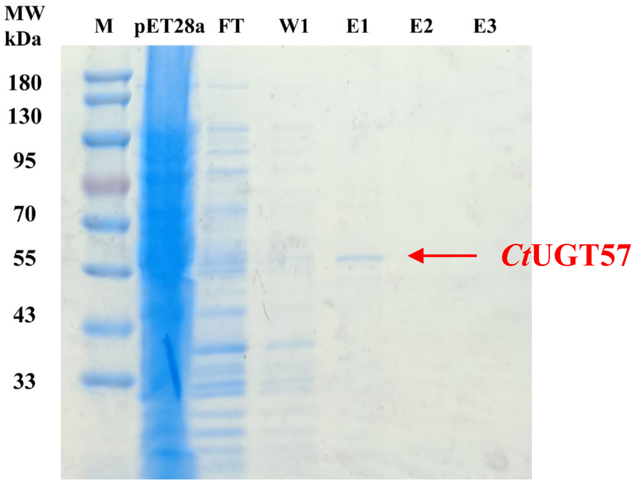

Figure S10. SDS-PAGE of the His-tagged *Ct*UGT57.

M: Marker; FT: Flow Through; W1-W2: Wash Solution 1-2; E1-E3: Elution Solution 1-3

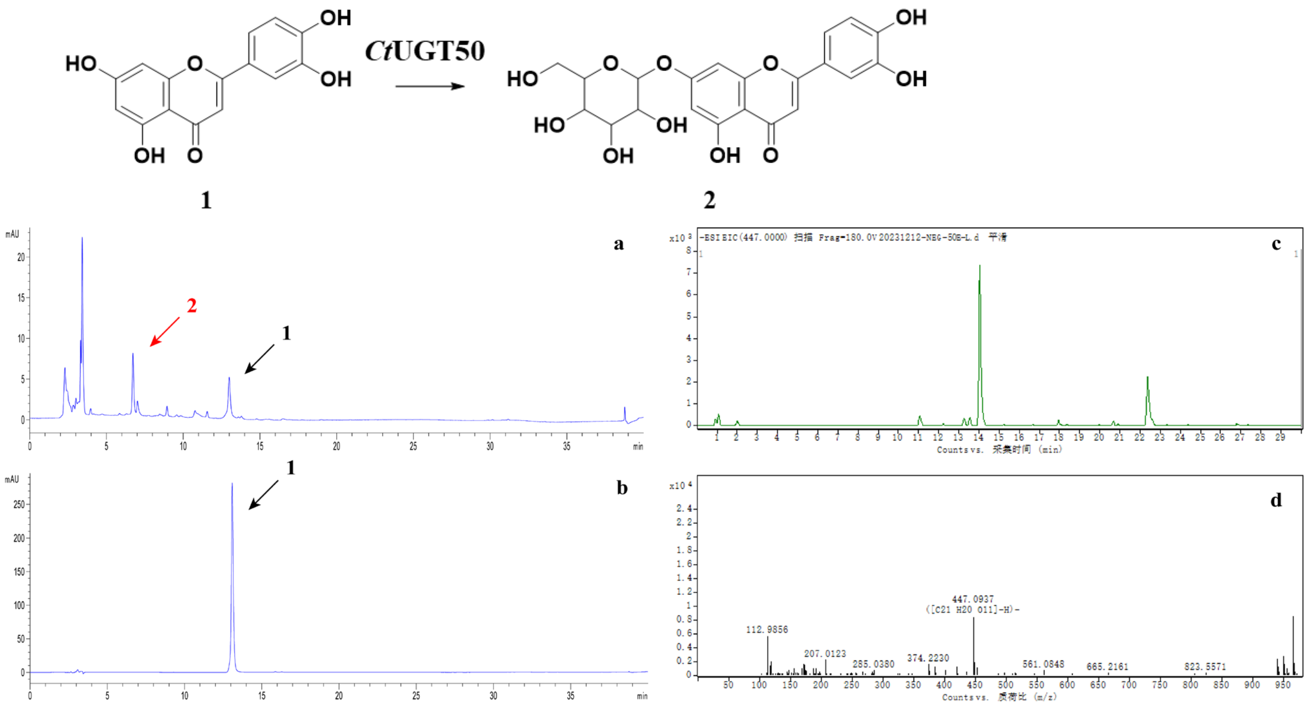

Figure S11. Results of Luteolin glycosylation reaction catalyzed by *Ct*UGT50.

a: HPLC result of *Ct*UGT50 (crude) after reaction with Luteolin (1) and UDP-glucose;

b: HPLC result of substrate and buffer with the absence of *Ct*UGT50;

c-d: LC-MS analysis of *Ct*UGT50 catalyzed reaction results

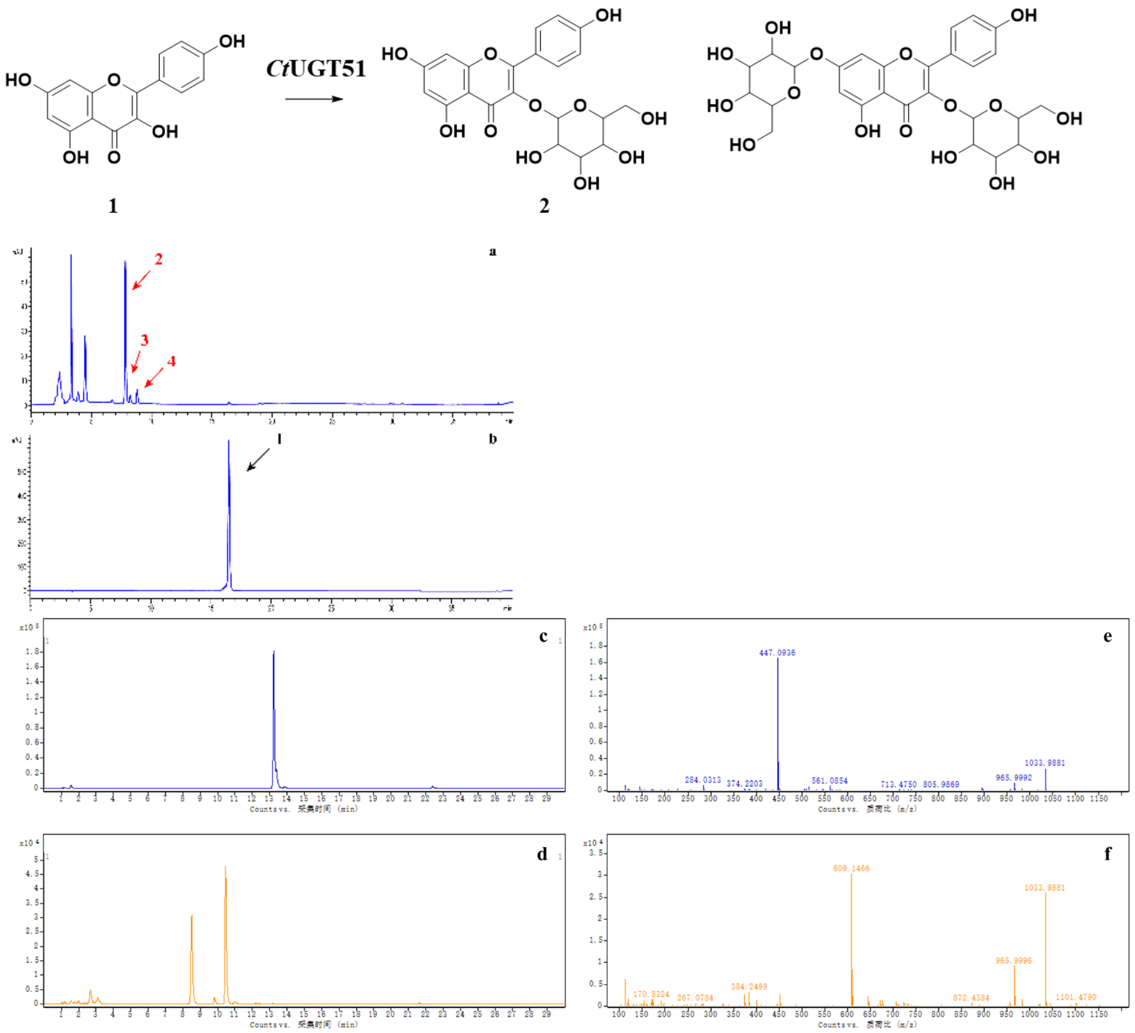

Figure S11. Results of Kaempferol glycosylation reaction catalyzed by *Ct*UGT51.

a: HPLC result of *Ct*UGT51 after reaction with Kaempferol (1) and UDP-glucose;

b: HPLC analysis of Kaempferol;

c-d: LC-MS analysis of *Ct*UGT51 catalyzed reaction results (Neg m/z 447);

e-f: LC-MS analysis of *Ct*UGT51 catalyzed reaction results (Neg m/z 609)

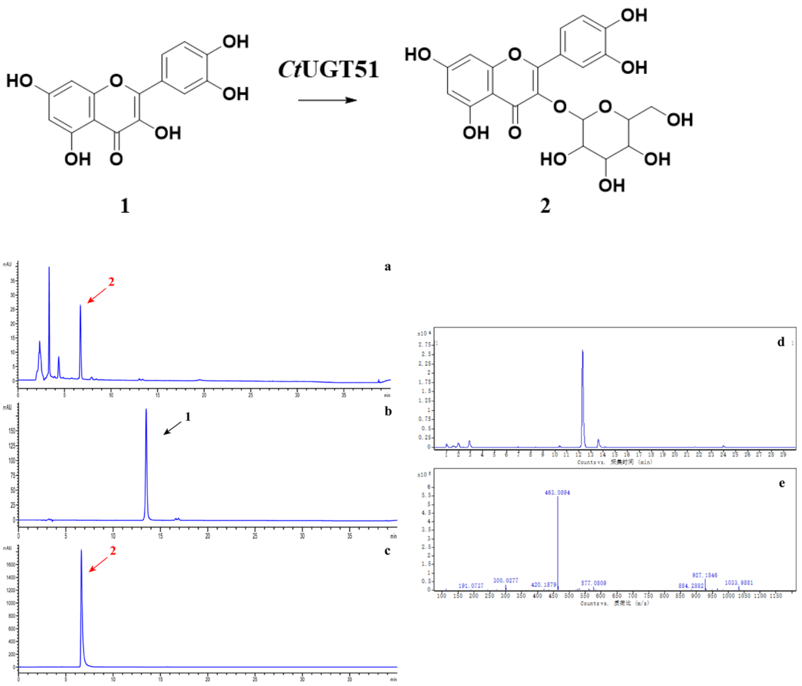

Figure S11. Results of Quercetin glycosylation reaction catalyzed by *Ct*UGT51.

a: HPLC result of *Ct*UGT51 after reaction with Quercetin (1) and UDP-glucose;

b-c: HPLC analysis of Quercetin (1) and Isoquercetin (2);

d-e: LC-MS analysis of *Ct*UGT51 catalyzed reaction results (Neg m/z 463)

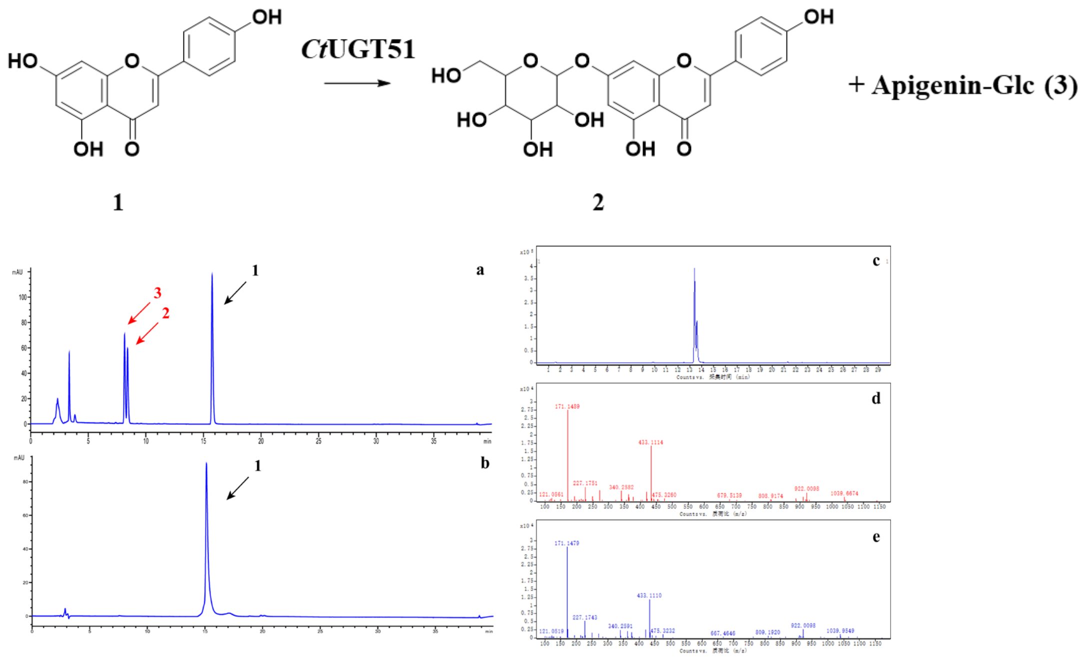

Figure S11. Results of Apigenin glycosylation reaction catalyzed by *Ct*UGT51.

a: HPLC result of *Ct*UGT51 after reaction with Apigenin (1) and UDP-glucose;

b: HPLC analysis of Apigenin;

c-e: LC-MS analysis of *Ct*UGT51 catalyzed reaction results (Pos m/z 433)

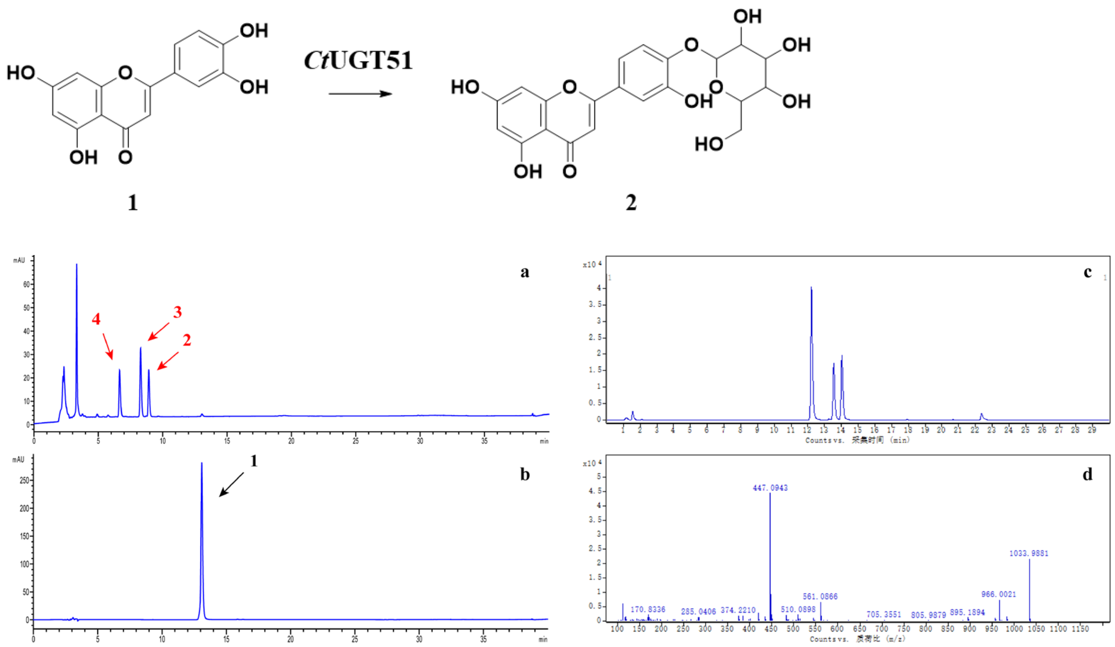

Figure S11. Results of Luteolin glycosylation reaction catalyzed by *Ct*UGT51.

a: HPLC result of *Ct*UGT51 after reaction with Luteolin (1) and UDP-glucose;

b: HPLC analysis of Luteolin;

c-d: LC-MS analysis of *Ct*UGT51 catalyzed reaction results (Neg m/z 447)

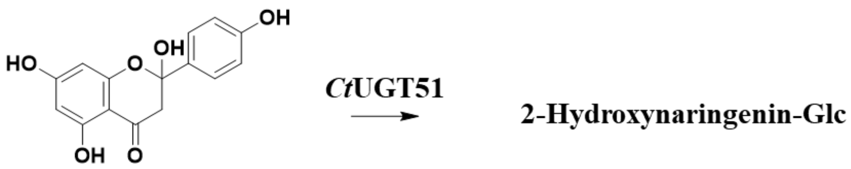

1 2

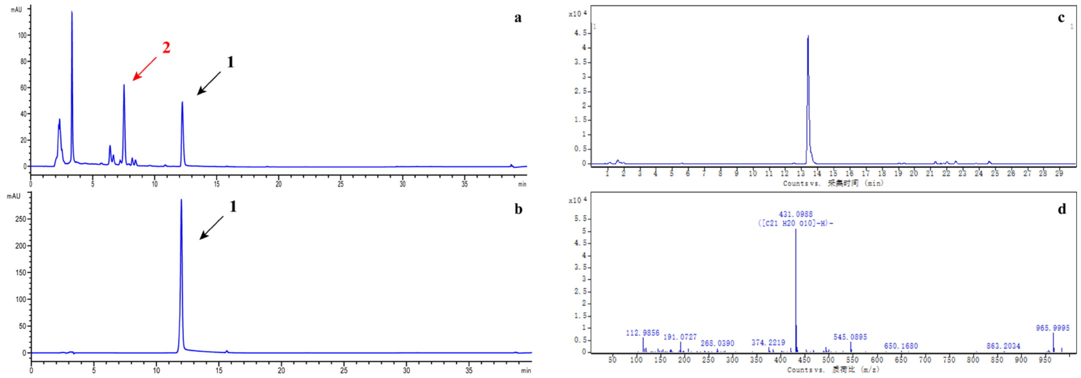

Figure S11. Results of 2-Hydroxynaringenin glycosylation reaction catalyzed by *Ct*UGT51.

a: HPLC result of *Ct*UGT51 reacted with 2-Hydroxynaringenin (1) and UDP-glucose;

b: HPLC analysis of 2-Hydroxynaringenin;

c-d: LC-MS analysis of *Ct*UGT51 catalyzed reaction results (Neg m/z 431)

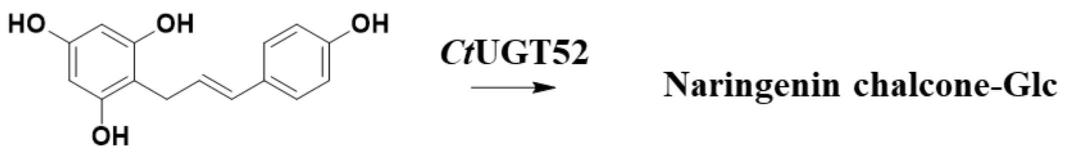

1 **2**

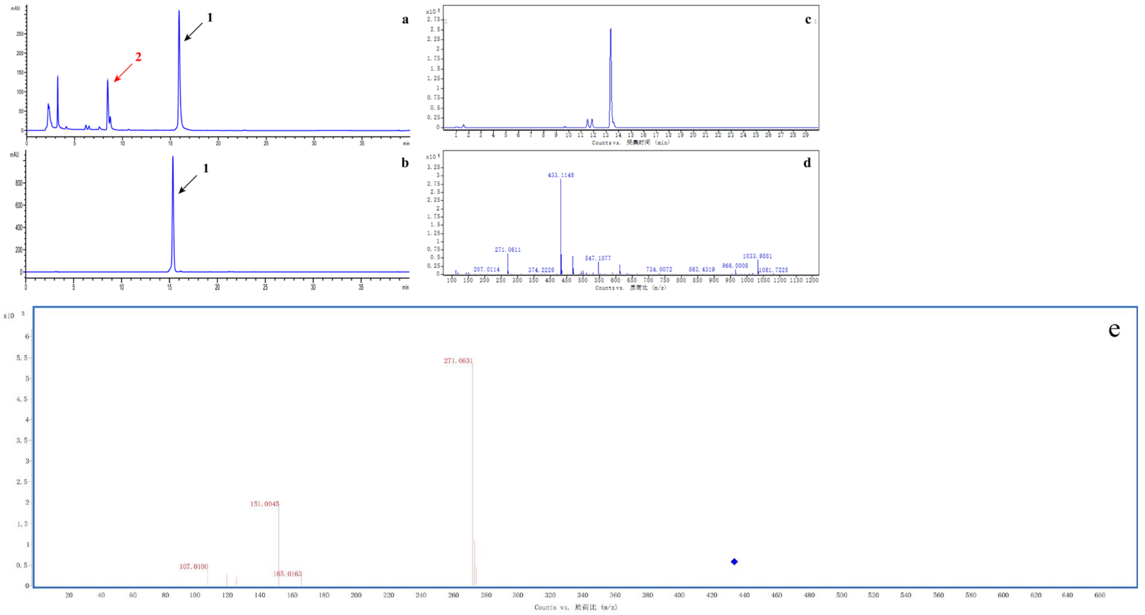

Figure S11. Results of Naringenin chalcone glycosylation reaction catalyzed by *Ct*UGT52.

a: HPLC result of *Ct*UGT52 after reaction with Naringenin chalcone (1) and UDP-glucose;

b: HPLC analysis of Naringenin chalcone;

c-d: LC-MS analysis of *Ct*UGT52 catalyzed reaction results (Neg m/z 433); e: LC-MS/MS analysis of compound 2

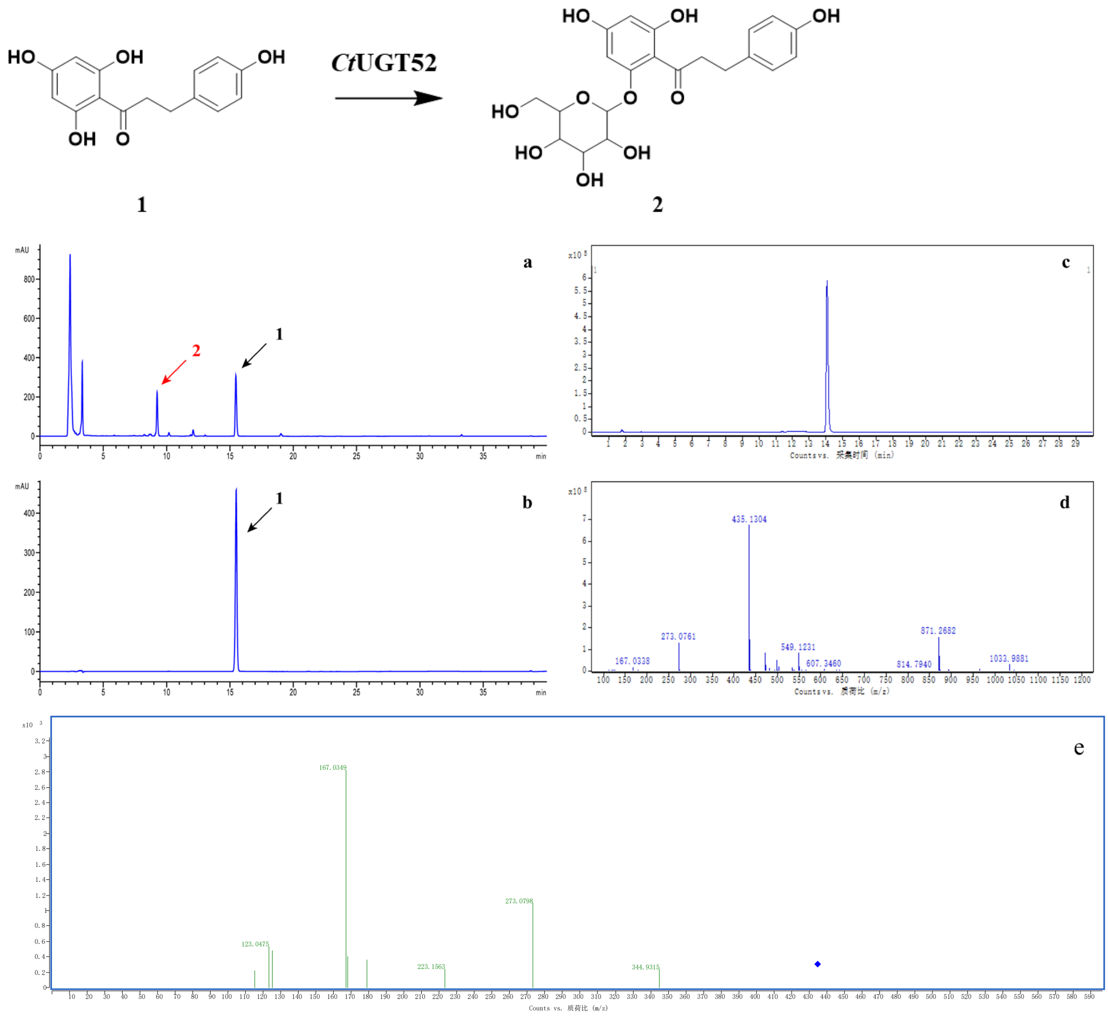

Figure S11. Results of Phloretin glycosylation reaction catalyzed by *Ct*UGT52.

a: HPLC result of *Ct*UGT52 after reaction with Phloretin (1) and UDP-glucose;

b: HPLC analysis of Phloretin;

c-d: LC-MS analysis of *Ct*UGT52 catalyzed reaction results (Neg m/z 435);

e: LC-MS/MS analysis of compound 2

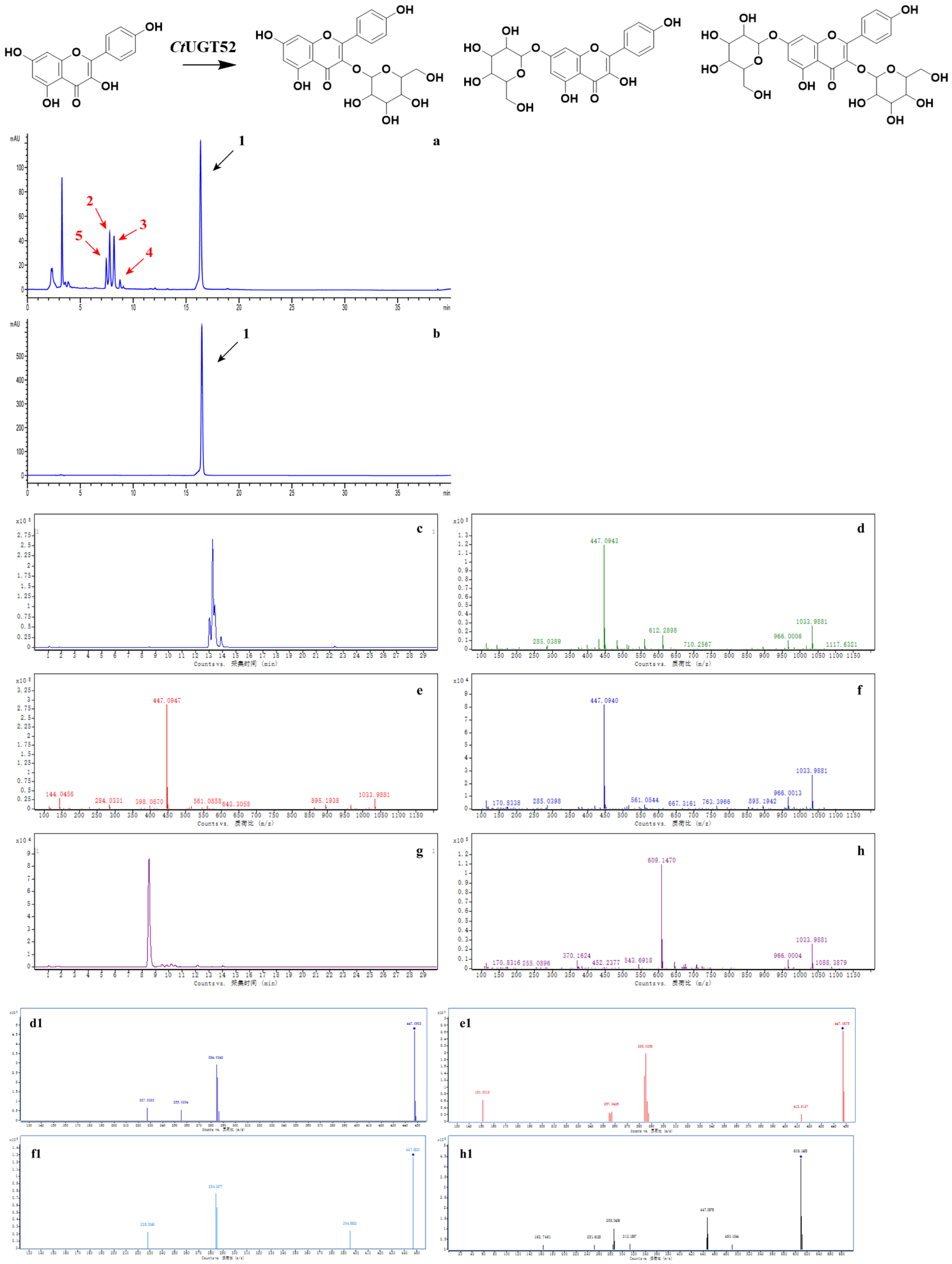

Figure S11. Results of Kaempferol glycosylation reaction catalyzed by *Ct*UGT52.

a: HPLC result of *Ct*UGT52 after reaction with Kaempferol (1) and UDP-glucose;

b: HPLC analysis of Kaempferol;

c-f: LC-MS analysis of *Ct*UGT52 catalyzed reaction results (Neg m/z 447);

g-h: LC-MS analysis of *Ct*UGT52 catalyzed reaction results (Neg m/z 609);

d1-h1: LC-MS/MS analysis of compound 2-5

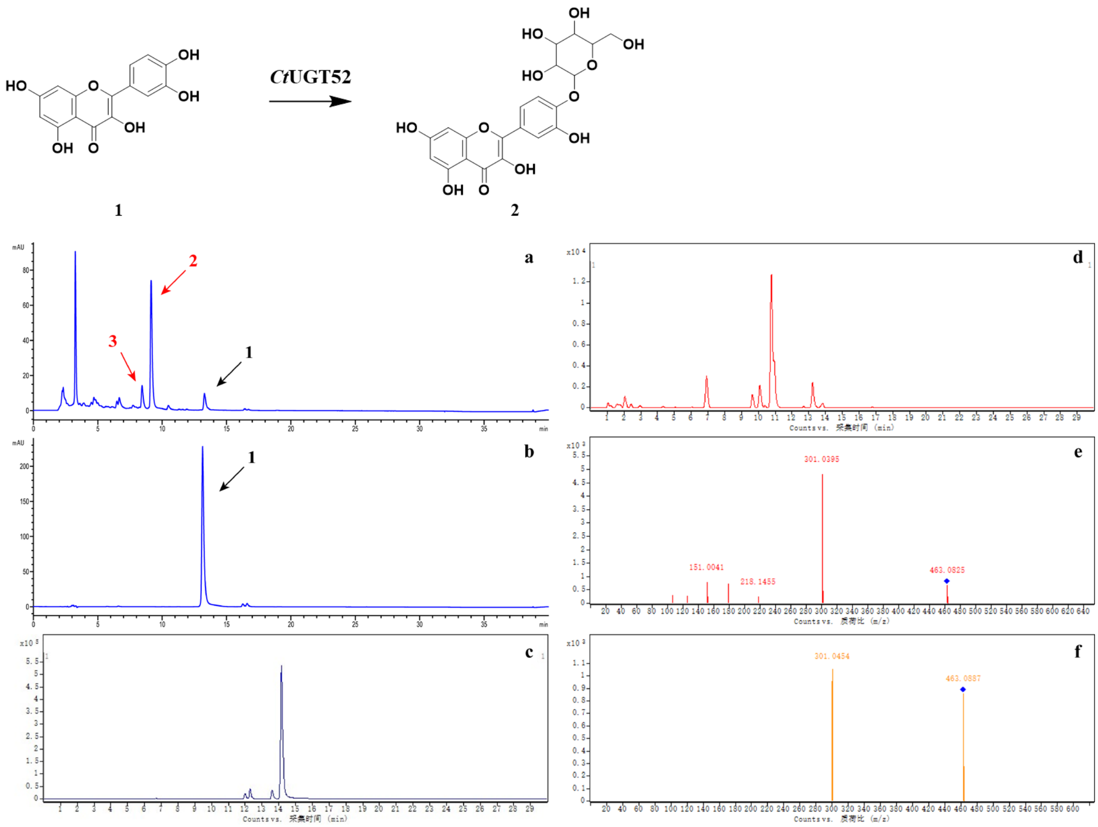

Figure S11. Results of Quercetin glycosylation reaction catalyzed by *Ct*UGT52.

a: HPLC result of *Ct*UGT52 after reaction with Quercetin (1) and UDP-glucose;

b: HPLC analysis of Quercetin;

c-d: LC-MS analysis of *Ct*UGT52 catalyzed reaction results (Neg m/z 463, 625);

e-f: LC-MS/MS analysis of *Ct*UGT52 catalyzed reaction results (Neg m/z 463)

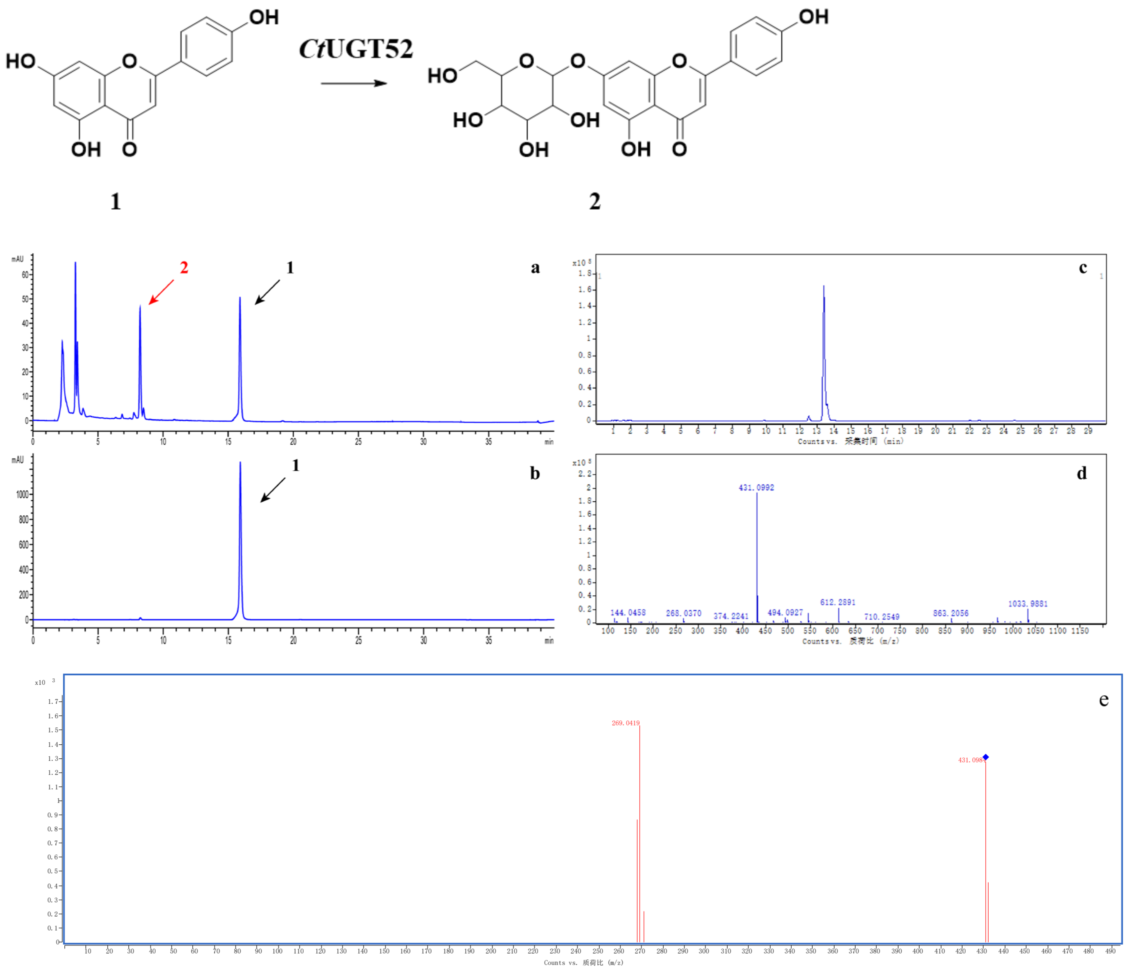

Figure S11. Results of Apigenin glycosylation reaction catalyzed by *Ct*UGT52.

a: HPLC result of *Ct*UGT52 after reaction with Apigenin (1) and UDP-glucose;

b: HPLC analysis of Apigenin;

c-d: LC-MS analysis of *Ct*UGT52 catalyzed reaction results (Neg m/z 431);

e: LC-MS/MS analysis of compound 2

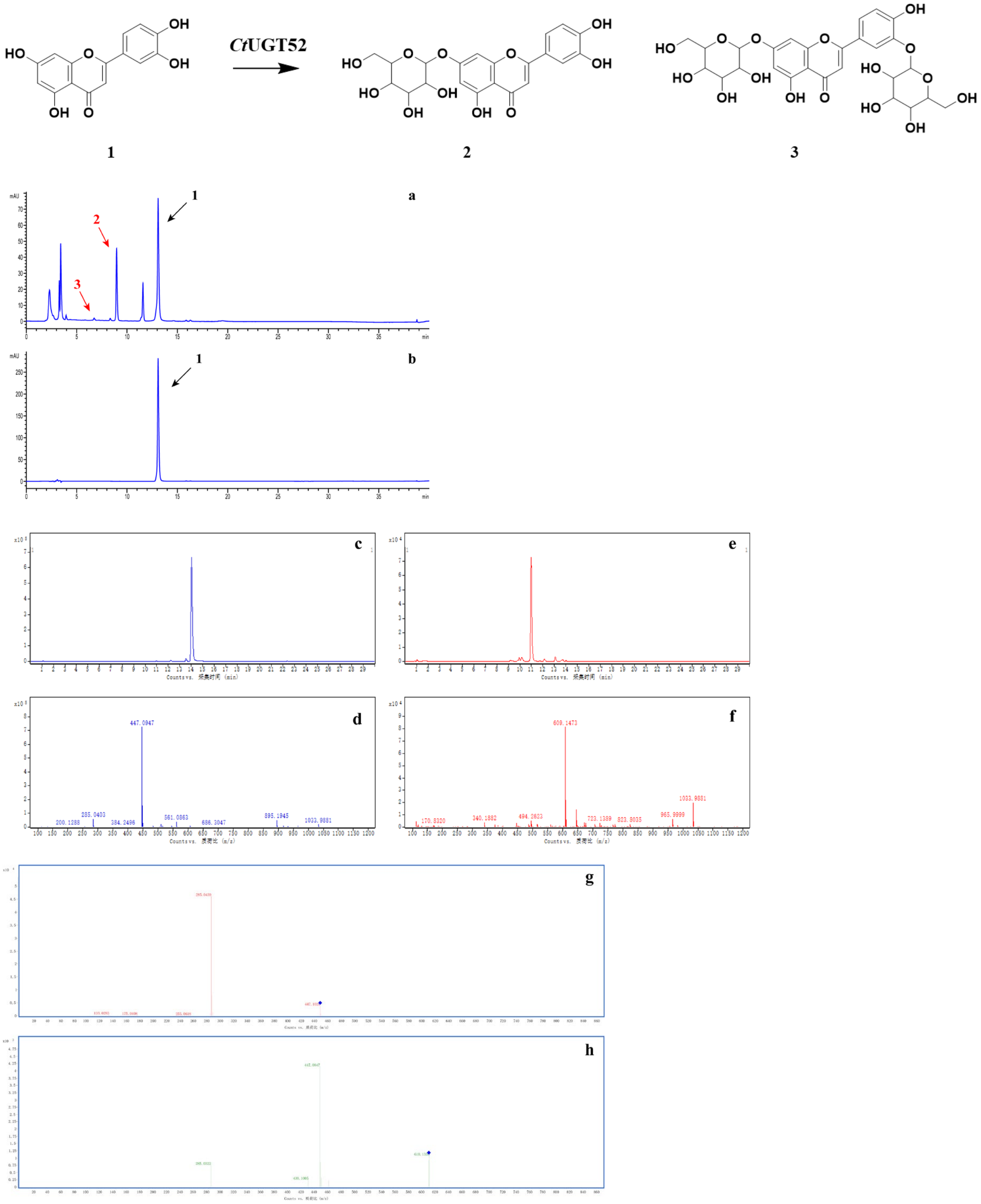

Figure S11. Results of Luteolin glycosylation reaction catalyzed by *Ct*UGT52.

a: HPLC result of *Ct*UGT52 after reaction with Luteolin (1) and UDP-glucose; b: HPLC analysis of Luteolin; c-d: LC-MS analysis of *Ct*UGT52 catalyzed reaction results (Neg m/z 447); e-f: LC-MS analysis of *Ct*UGT52 catalyzed reaction results (Neg m/z 609); g-h: LC-MS/MS analysis of compound 2 and 3

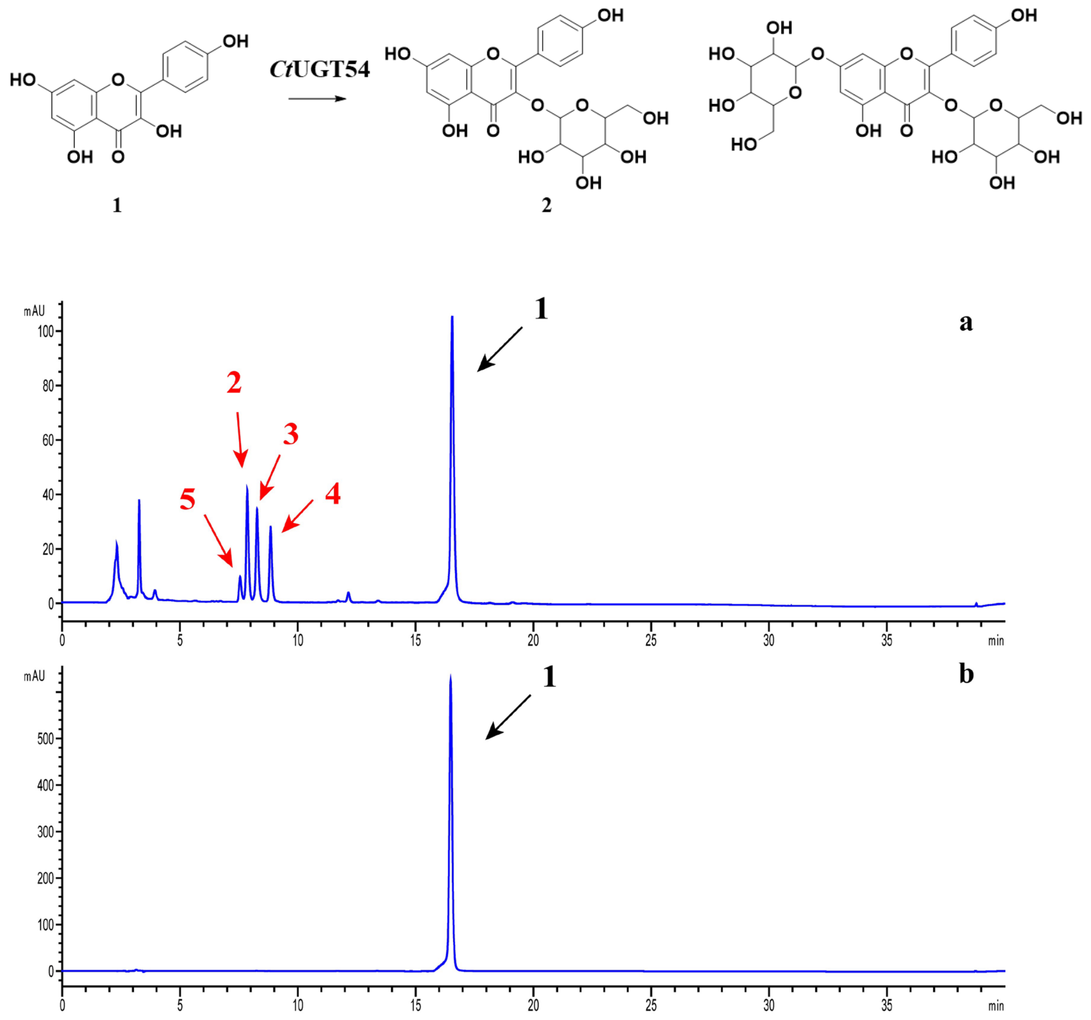

Figure S11. Results of Kaempferol glycosylation reaction catalyzed by *Ct*UGT54.

a: HPLC result of *Ct*UGT54 after reaction with Quercetin (1) and UDP-glucose;

b: HPLC analysis of Kaempferol;

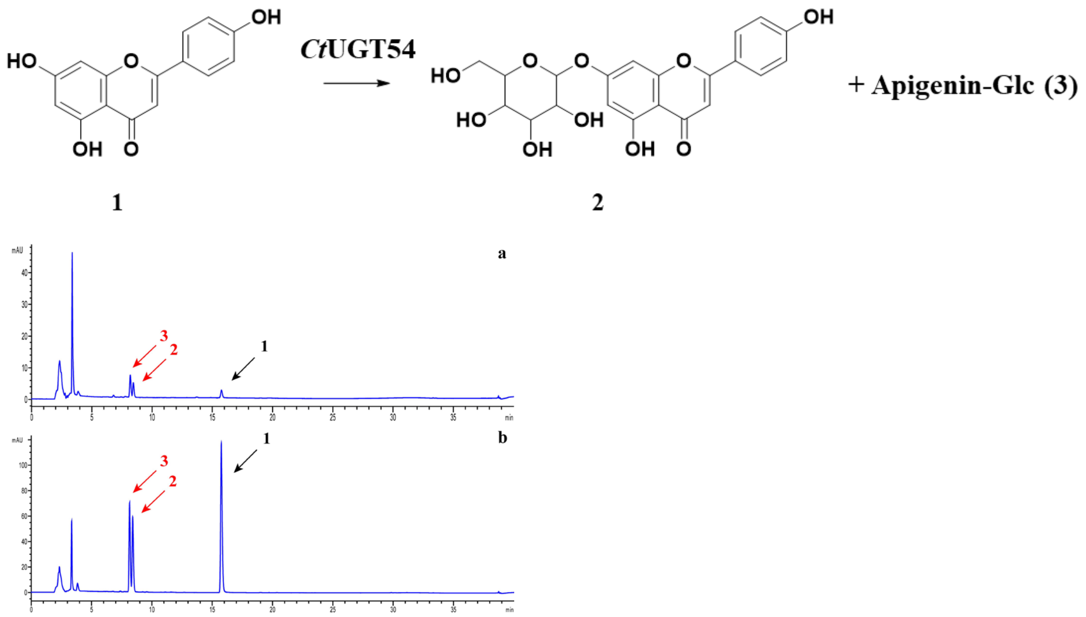

Figure S11. Apigenin glycosylation comparison between *Ct*UGT54 (a) and *Ct*UGT51 (b).

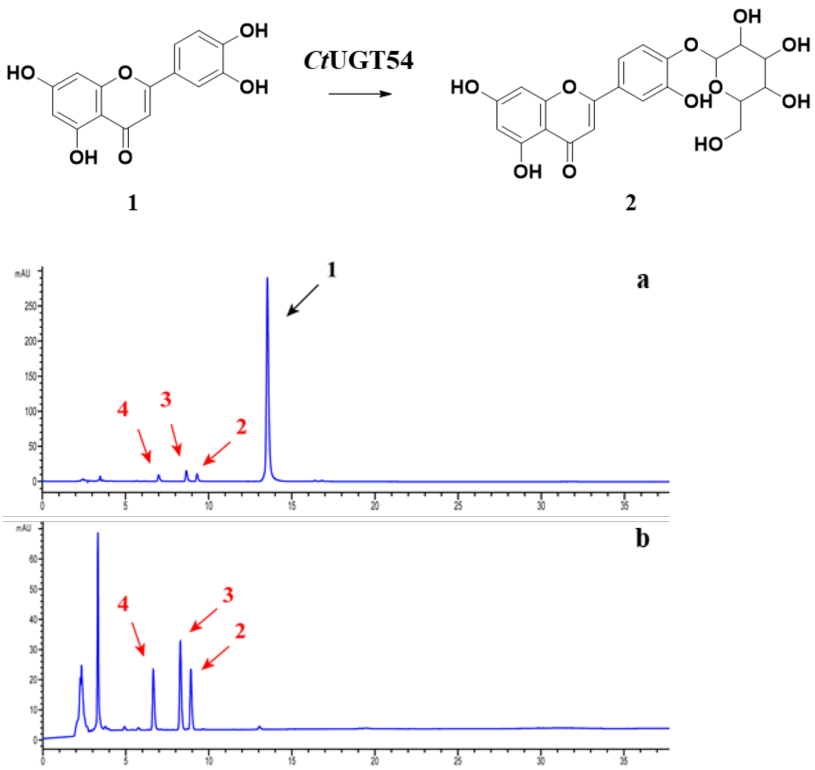

Figure S11. Luteolin glycosylation comparison between *Ct*UGT54 (a) and *Ct*UGT51 (b).

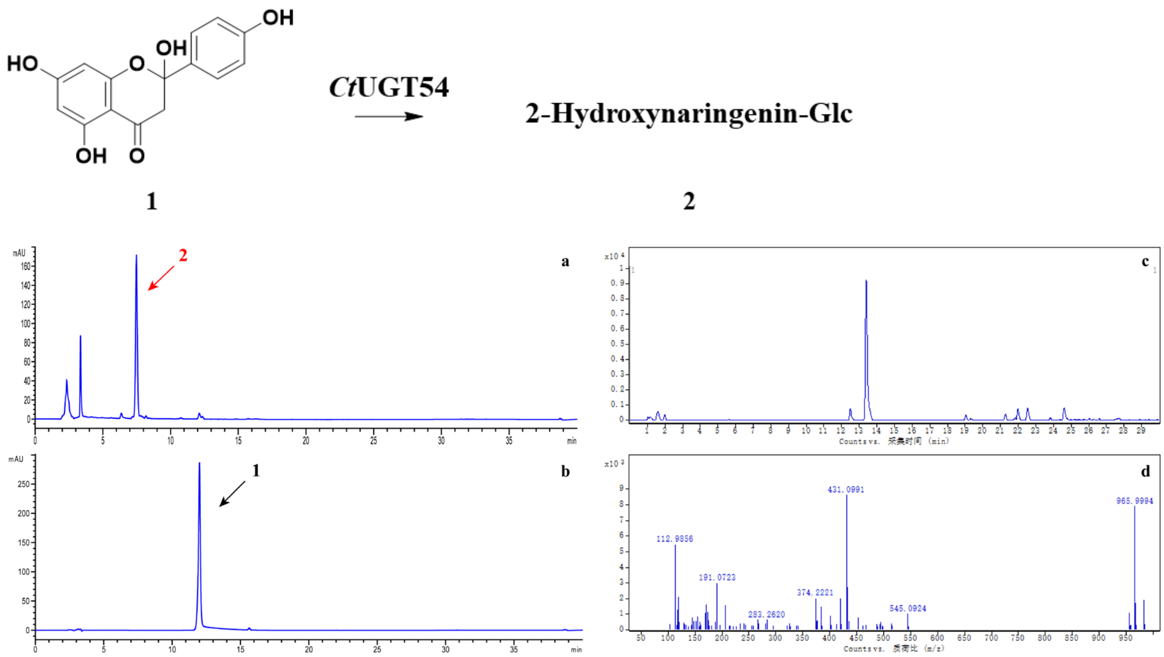

Figure S11. Results of the 2-Hydroxynaringenin glycosylation catalyzed by *Ct*UGT54.

a: Reaction result of *Ct*UGT54 catalyzed 2-Hydroxynaringenin (1) and UDP-Glc;

b: HPLC analysis of 2-Hydroxynaringenin;

c-d: LC-MS analysis of 2-Hydroxynaringenin glycosylation (Neg m/z 417)

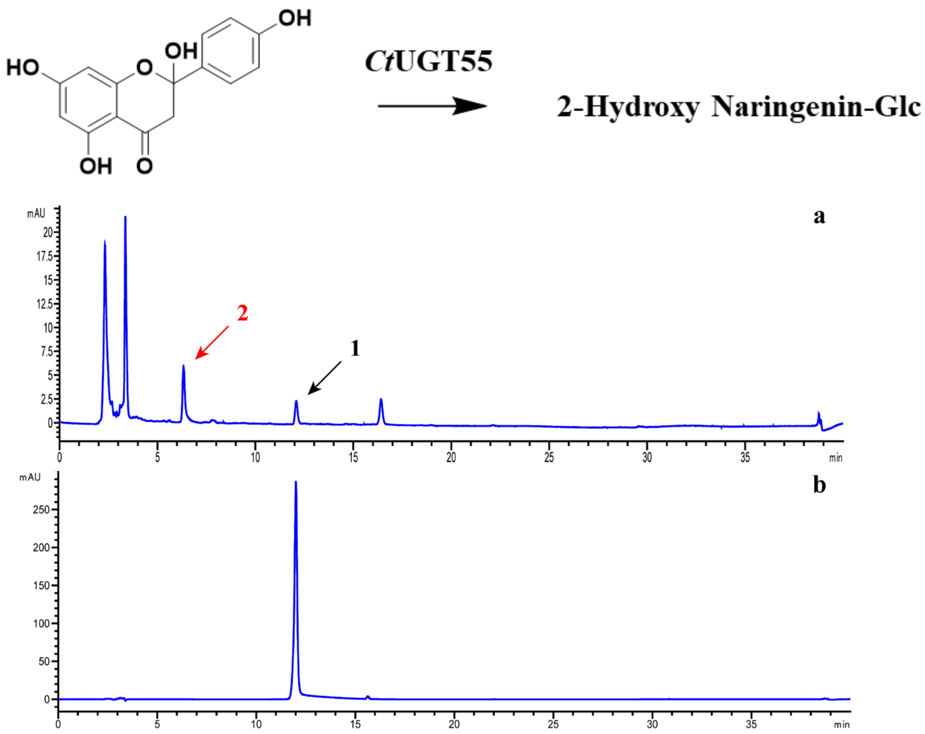

Figure S11. Results of 2-Hydroxynaringenin glycosylation reaction catalyzed by *Ct*UGT55.

a: HPLC result of *Ct*UGT55 reacted with 2-Hydroxynaringenin (1) and UDP-glucose;

b: HPLC analysis of 2-Hydroxynaringenin

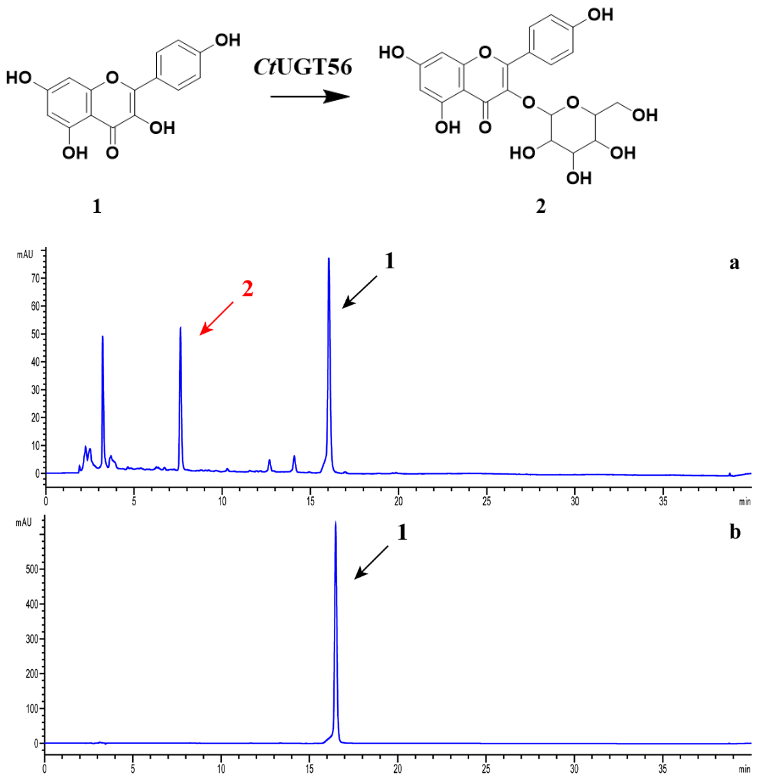

Figure S11. Results of Kaempferol glycosylation reaction catalyzed by *Ct*UGT56.

a: HPLC result of *Ct*UGT56 after reaction with Kaempferol (1) and UDP-glucose;

b: HPLC analysis of Kaempferol

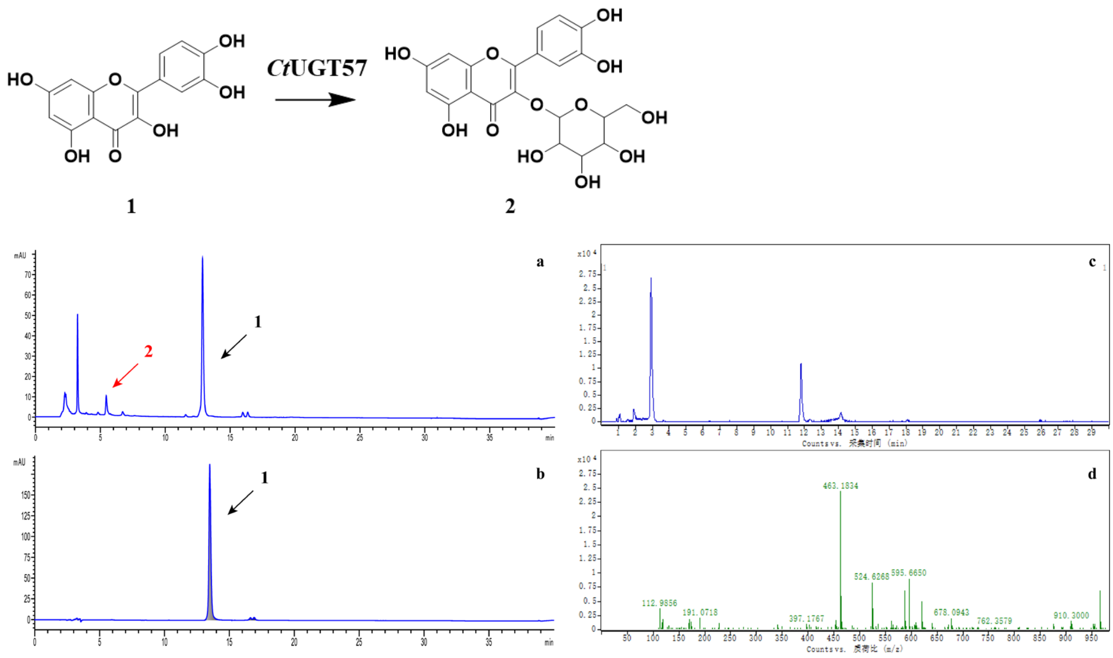

Figure S11. Results of the Quercetin glycosylation catalyzed by *Ct*UGT57.

a: Reaction result of *Ct*UGT57 catalyzed Quercetin (1);

b: HPLC analysis of Quercetin;

c-d: LC-MS analysis of Quercetin glycosylation (Neg m/z 463)

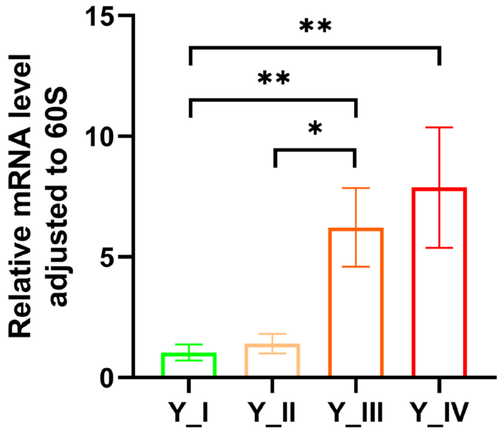

Figure S12. Relative expression Levels of *CtUGT52* during different growing stages. (Mean ± SEM (n=3), * $P < 0.05$, ** $P < 0.01$, *** $P < 0.001$)

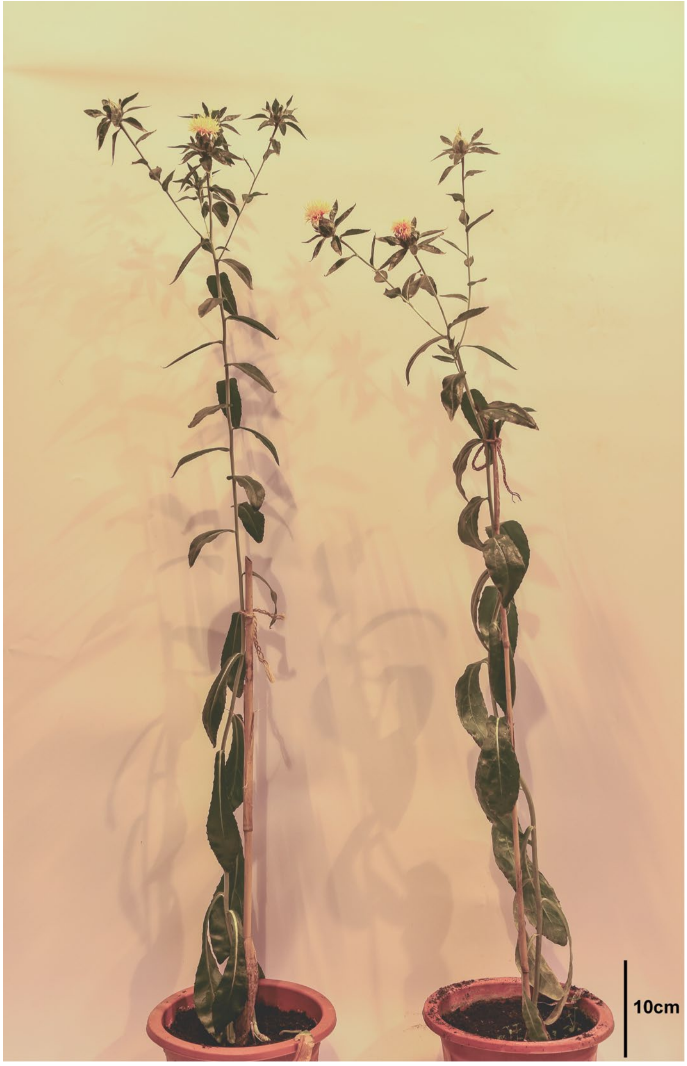

Figure S13. Comparison of wild-type (left) and *CtUGT52* overexpressed safflower (right).

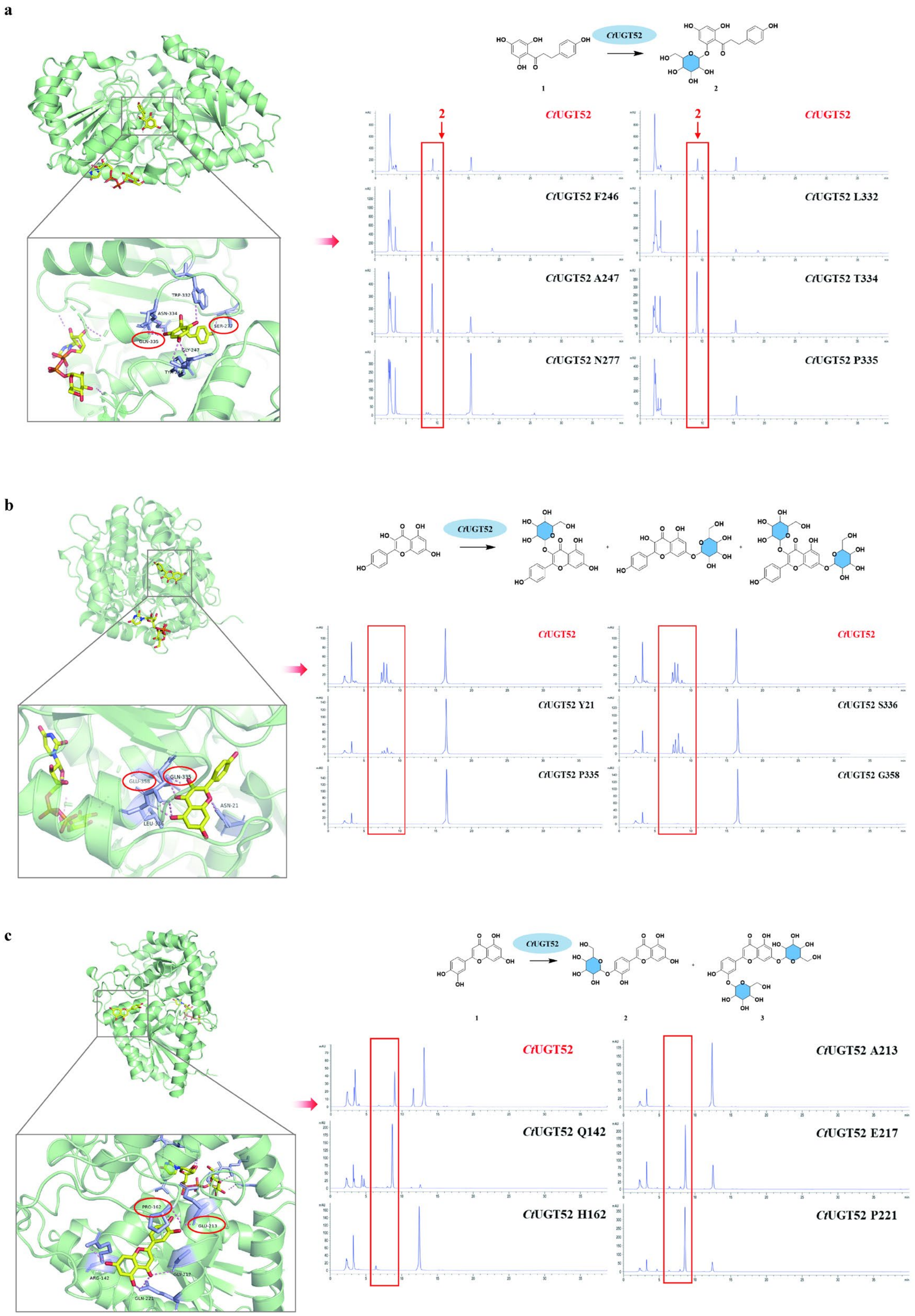

Figure S14. Analysis of the key binding sites of *Ct*UGT52 with Phloretin (a), Kaempferol (b), Luteolin (c) and UDP-Glc.

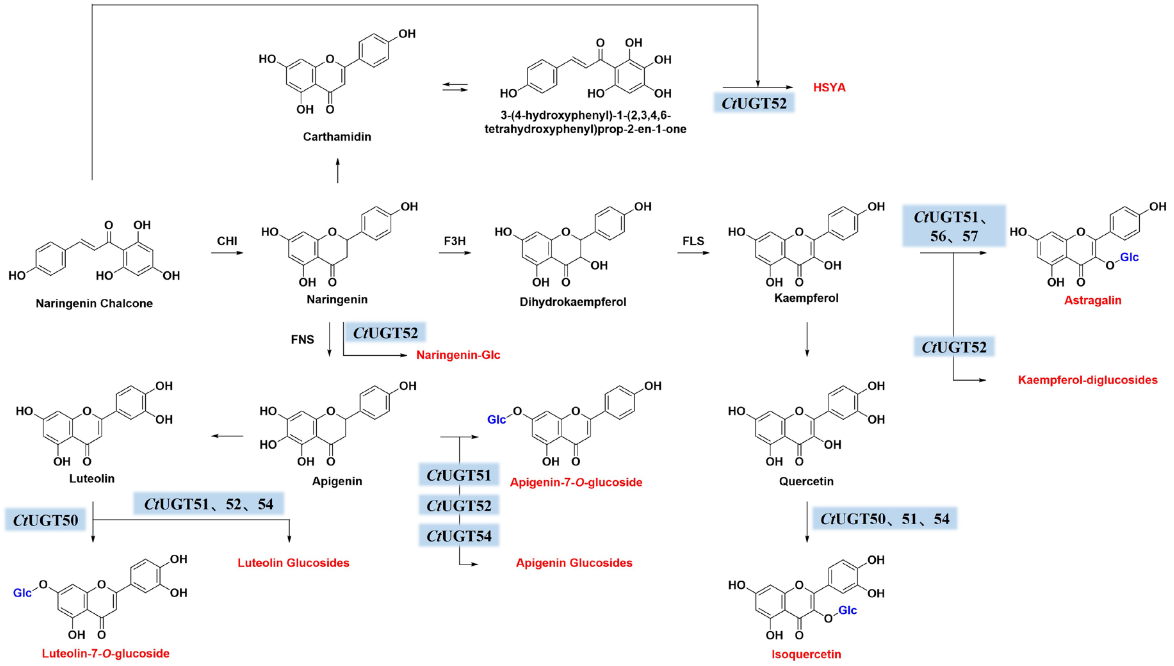

Figure S15. Identified *Ct*UGTs of the different flavonoid glycosides biosynthesis in yellow safflower.

Table S4. Amplification primer sequences of *CtUGTs* recombinant plasmid.

Primer	Sequence(5'-3')
pET28a-*CtUGT50* F	ATGGGTCGCGGATCCGAATTCATGAATCAAGTCGTAATGATACCGTT
pET28a-*CtUGT50* R	GCAAGCTTGTCGACGGAGCTCTTTCTTTCGGTCCGTTACAAACT
pET28a-*CtUGT51* F	ATGGGTCGCGGATCCGAATTCATGCCGAATACAGTCGCAGAG
pET28a-*CtUGT51* R	GCAAGCTTGTCGACGGAGCTCTGAGATGTTTCTCATAAAATCCTCGA
pET28a-*CtUGT52* F	ATGGGTCGCGGATCCGAATTCATGGAGAAAAGCTACAAAGGTCATG
pET28a-*CtUGT52* R	CAAGCTTGTCGACGGAGCTCGTTATTTTTTCTTGCAAATGTCTTCAA
pET28a-*CtUGT53* F	ATGGGTCGCGGATCCGAATTCATGGTATATGAAGTCAATGAATACATGG
pET28a-*CtUGT53* R	GCAAGCTTGTCGACGGAGCTCAGATGCGTGTTTTGAACGTGG
pET28a-*CtUGT54* F	ATGGGTCGCGGATCCGAATTCATGGAGGAGAAAGTAGAGGTGTTTTT
pET28a-*CtUGT54* R	GCAAGCTTGTCGACGGAGCTCCATGAAATCGTCTACAAGGTCTTTCA
pET28a-*CtUGT55* F	ATGGGTCGCGGATCCGAATTCATGCCGACCGCCACCGTC
pET28a-*CtUGT55* R	GCAAGCTTGTCGACGGAGCTCCACCTTATGAGAGCTCCCTCCC
pET28a-*CtUGT56* F	ATGGGTCGCGGATCCGAATTCATGGCGGACTCCATCACCTC
pET28a-*CtUGT56* R	GCAAGCTTGTCGACGGAGCTCATTGTTTCGACTGATCAAAATGTCA
pET28a-*CtUGT57* F	ATGGGTCGCGGATCCGAATTCATGGAAGAAGGTGGAAGACGACT
pET28a-*CtUGT57* R	GCAAGCTTGTCGACGGAGCTCCACTAAACTCTCCAATGAATCATGG
pMT39-*CtUGT52* F	CCACCGCGGCCGCCACCATGGATGGAGAAAAGCTACAAAGGTCATG
pMT39-*CtUGT52* R	CCTCGCCCTTGCTCACCATGGGTTATTTTTTCTTGCAAATGTCTTCAA
CtUGT52 qF	GTGAGAATGGAGGATTGT
CtUGT52 qR	CTTGGAAAGTATTGCTGAAA
CtUGT52-ovx F	CGACAGTGGTCCCAAAGAT
CtUGT52-ovx R	CTTGGAAAGTATTGCTGAAA

Table S5. Primer sequences for site-directed mutagenesis and recombinant plasmid amplification.

Primer	Sequence(5'-3')
*CtUGT52*Y21 F	AGCCAAGGCCACAT*CT*ACCCC*CT*CC*T*CCAATTCGC
*CtUGT52*Y21 R	TAGATGTGGC*CT*TGG*CT*TGGATAAGGTAACAC
*CtUGT52*Q142 F	TTCAGCCAGATATACGCAGGGACGTTGAGGTT
*CtUGT52*Q142 R	GCGTATATCTGGCTGAAAATGGCCGACACCGC
*CtUGT52*H162 F	TGGTGCTGCACGGGATTCCGCCGTTGGATTTG
*CtUGT52*H162 R	AATCCCGTGCAGCACCACCGGACAATCCTCCA
*CtUGT52*A213 F	TTGGAAGATGCGGTGGTGCAAGGATTGGAGGA
*CtUGT52*A213 R	ACCACCGCATCTTCCAATGCTTGGAAAGTATT
*CtUGT52*E217 F	GGTGCAAGAATTGGAGGAGCAATGGCCAGCAA
*CtUGT52*E217 R	CCTCCAATTCTTGCACCACCTCATCTTCCAAT
*CtUGT52*P221 F	AGCCATGGCCAGCAAAACTAATAGGTCCAATG
*CtUGT52*P221 R	TTTTGCTGGCCATGGCTCCTCCAATCCTTGCACC
*CtUGT52*F246 F	CAAAGGGTTTGGTGCAAGTCTATGGAAACCAC
*CtUGT52*F246 R	TTGCACCAAACCCTTTGTCACCTTCAATTCTTT
*CtUGT52*A247 F	AGGGTATGCTGCAAGTCTATGGAAACCACTTGG
*CtUGT52*A247 R	GACTTGCAGCATACCCTTTGTCACCTTCAATTCT
*CtUGT52*N277 F	CTTTGGGACCATGGTGTCCTTAAGCCAGCAAG
*CtUGT52*N277 R	ACACCATGGTCCCAAAGGAAATGTAGATAACTGAG
*CtUGT52*L332 F	TGTGCAACCAATTAGAGATACTAGCTCGTAAA
*CtUGT52*L332 R	CTCTAATTGGTTGCACAAATTTACTATCATACCCTTTTCTTGATTTT
*CtUGT52*F333 F	GGTTCAACCAATTAGAGATACTAGCTCGTAAA
*CtUGT52*F333 R	CTCTAATTGGTTGAACCAATTTACTATCATACCCTTTTCTTG
*CtUGT52*T334 F	TTGGTGCAGCCAATTAGAGATACTAGCTCGTAAATCA
*CtUGT52*T334 R	CTAATTGGCTGCACCAATTTACTATCATACCCTTT
*CtUGT52*P335 F	GTGCAACCCATTAGAGATACTAGCTCGTAAATCAGTGG
*CtUGT52*P335 R	TCTCTAATGGGTTGCACCAATTTACTATCATACCC
*CtUGT52*S336 F	GTGCAACCAATCAGAGATACTAGCTCGTAAATCAGTGGG
*CtUGT52*S336 R	TCTCTGATTGGTTGCACCAATTTACTATCATAC
*CtUGT52*G358 F	ACTGGGAGGGTTGAGTTTGGGTGTGCCGATGG
*CtUGT52*G358 R	AACTCAACCCTCCCAGTGTCGAGTTCCACCCA
