# Supplementary material for: BSA-seq integrated with transcriptomics and metabolomics revealing the candidate genes associated with safflower colors and flavonoid glycosides biosynthesis
Source: Hortic Res. 2026 Mar 4;13(6):uhag068. doi: 10.1093/hr/uhag068 (PMC13253347; doi:10.1093/hr/uhag068)
Supplement: Web_Material_uhag068 [file web_material_uhag068.zip › Supplementary Table.docx]

Table S1. The sequencing depth of BSA analysis.

| **Chromosome** | **Mapped_Reads** | **Unmapped_Reads** | **Mean Depth** | **Coverage_Rate(1x)** | **Coverage_Rate(5x)** | **Coverage_Rate(10x)** |
| --- | --- | --- | --- | --- | --- | --- |
| *Ct*AH01 | 28948550 | 126240 | 43.82350436 | 0.993411693 | 0.989375291 | 0.98305224 |
| *Ct*AH02 | 37248822 | 156989 | 50.94440091 | 0.99791496 | 0.996371872 | 0.99325474 |
| *Ct*AH03 | 35533302 | 150209 | 44.0878565 | 0.964848942 | 0.94860019 | 0.937483661 |
| *Ct*AH04 | 29734192 | 126390 | 43.75995377 | 0.974338499 | 0.963317227 | 0.955214219 |
| *Ct*AH05 | 31569938 | 152656 | 43.2410419 | 0.989669719 | 0.984514549 | 0.977560011 |
| *Ct*AH06 | 31130943 | 144127 | 44.62487386 | 0.986705871 | 0.980800525 | 0.975282466 |
| *Ct*AH07 | 26377179 | 114545 | 48.84638 | 0.99362107 | 0.989906338 | 0.985823956 |
| *Ct*AH08 | 30994100 | 161549 | 46.06381545 | 0.977694318 | 0.969725984 | 0.960722522 |
| *Ct*AH09 | 27926170 | 119584 | 43.30891965 | 0.981427176 | 0.972279326 | 0.964879397 |
| *Ct*AH10 | 34047853 | 141723 | 52.5526991 | 0.992409212 | 0.98781207 | 0.977805817 |
| *Ct*AH11 | 24927606 | 108938 | 44.78087573 | 0.979755805 | 0.970034434 | 0.962434065 |
| *Ct*AH12 | 38658572 | 226805 | 58.73450645 | 0.972856791 | 0.959594085 | 0.949910155 |

Table S2. Basic information of target *CtUGTs.*

| Name |  |
| --- | --- |
| *CtUGT50*（*Ct*AH10G0075800） | Chrom: *Ct*AH10（Location: 12692989-12695463） |
|  | Molecular Weight: 50891.24 Daltons; 454 Amino Acids |
| *CtUGT51*（*Ct*AH03G0193500） | Chrom: *Ct*AH03（Location: 78922619-78925082） |
|  | Molecular Weight: 53484.66 Daltons; 486 Amino Acids |
| *CtUGT52*（*Ct*AH02G0117700) | Chrom: *Ct*AH02（Location: 20128343-20131008） |
|  | Molecular Weight: 51602.31 Daltons; 463 Amino Acids |
| *CtUGT53*（*Ct*AH06G0198500） | Chrom: *Ct*AH06（Location: 61643406-61649347） |
|  | Molecular Weight: 54038.73 Daltons; 471 Amino Acids |
| *CtUGT54*（*Ct*AH08G0149200） | Chrom: *Ct*AH08（Location: 18915565-18916974） |
|  | Molecular Weight: 52939.94 Daltons; 469 Amino Acids |
| *CtUGT55*（*Ct*AH01G0016500） | Chrom: *Ct*AH01（Location: 2035727-2053783） |
|  | Molecular Weight: 53229.51 Daltons; 491 Amino Acids |
| *CtUGT56*（*Ct*AH10G0065700） | Chrom: *Ct*AH10（Location: 10537371-10539956） |
|  | Molecular Weight: 50911.42 Daltons; 454 Amino Acids |
| *CtUGT57*（*Ct*AH09G0287800） | Chrom: *Ct*AH09（Location: 84723105-84728932） |
|  | Molecular Weight: 48188.96 Daltons; 429 Amino Acids |

Table S3. Sequence details of target *CtUGTs.*

|  | **Nucleic acid sequence (CDS)** |
| --- | --- |
| ***CtUGT50*** | ATGAATCAAGTCGTAATGATACCGTTTCCAGGCAGAGGTCACATAAACCCTATGTTAAACCTCTGCAAATCACTCTCCTCTCGAGTAAATCAATCCAATCGCACCACCGTCTTCACCGTCGTCGTCACCGAGGAGTGGCTCGGAATACTTAATCCGGATCTGAATCAACCTACCGTTCGTTTCGCTACCATTCCGAACGTCCTTCCATCGGAGCTCCATCGTGGATCTGACATGATCGCCTTCCTCACCGCCATCTGTACCAAGATGAAGCGTCCTTTCGAGGAGGTTCTAGATCGGATGGAGACGGAGATGGAGGTTCCGGTGGAGCTCATCATCGCCGATGCCGATATGCTTTGGCCGTTTGAGGTCGCTAACGAGAGGAAGATTCCGGTAGCGGCGTACTGGCCGTTGCCTGCCTCGGCGTTTTCCATGATGCACCATGCTGATCTACTTCGGTCGCACGGTCACATCGGCATTGATGTATCAGCAAGAGGAAAGGAGTGCATAGACTACATTCCTGGATTATCCCCATTCACAGTAGCAGAAATTCCAATGATACTTCATGGTGGCATCTTCAAACACTTAACAACTGATCTTTTCACCATAGCCCAAAAAGCAAATTGTCTTCTAATATCCACCCTTTACAACTTGGAATCCAAACCCATTGATGCCCTCAAATCCAGACTAAAAATACCCATCTTCACATCAGGCCTAAACATACCACCATCCCCCCAAATCAACAACCCCATTAACAACAACCCTCCTCAACCCATTTACATAAACTGGTTAAACTCCAAACCCCCCAAAACAGTCTTGTATATCTCTTTCGGCAGCTTTTTACCGATCCCCGATCACGAATTCGAAGAGATTGCCGCCGGTTTACTATCGAGCGGAGTTAGCTTCTTATGGGTCGGCCGAGGGAAAACCGAGAATTTGAAGGAAACTTTATGTCGTGGTGGAGATGGGAAAGCGATGGTGGTGGAGTGGTGTGATCAGCTGGCGGTTTTGTCGAACCCGGCGGTGGGTGGGTTTTGGACGCATTGCGGGTGGAATTCCGTTAAGGAAGGATTGTTTTCGGGTGTGCCGATGCTGACTTTTCCGTTGTTTCTCGATCAGCCGCTTAATGAGAAAATGATCGTTCGAGATTGGGGGGTTGGGAGGAAGGTGAGGGCGAACAGGGCGGAGGGGTTTACGAGGGGTGAGATTGTGGAGGTTGTTAGAGGGTTTATGGATACGGAGAGTGTTGAGAGGGTCGGGATGGTGGAGAGGGCGGAGAGGGTTCGGGAAATTTGTCGGGATGGTTTAACGGTGGACAGAGATCTTGAGGTCTTCGTTAGGGAGTTTGTAACGGACCGAAAGAAA |
| ***CtUGT51*** | ATGCCGAATACAGTCGCAGAGCTGGTGTTCATCCCTGCACCCGCGGTCGGTCATATCACGTCGACGGTCGAGATCGCAAAACTACTCGTGAACCGAGATCAACGCCTCTCGATAACCATCCTTGTCATCAAGCTGCCTCTGAGATTGGCTTCCGGCTCGGCTATCAAAGCCTACATCGATTCGTTGGCTAACAGGGCCTTGGACCGCGTATCCTTCGTCGAACTCCCGCAAGATGAAACCCCGCCATCTGGCGACCCAAAAGCTCCCATGACTTCTTTCGATGAATTCATCAACCGCCACTGCAAATACGTCAGAAACGTAGTGGCCGACATGATGAGTCAACCGGGTTCCGGCCGGGTGGTCGCCGGGTTCGTCATCGATATGTTTTGCACCGGCATGATCGATGTGGCGAACGAGTTTAACATTCCAACTTACGTGTTCTTTACTTCCAACGCCGCTTTTCTCGGATTTCAAATGCATATGTTGTCGCTCCGTGATGATCGGGACCGAGATCTCGCCGAATCGACCGACTCGGATGCTACGATACCCGTTCCGAGTTTCGTCAATCCGGTGCCAACGAAAGTATTTCCGTCAATGGTGAAGTCAGAAGGGCTGGATTTTGTTACGAGGATAGTCGGGAAAATGAGAGAGGCGAAGGCGATCGTGGTTAATACGTTCCTGGAATTGGAAACGCACGCGATCGAGTCGTTGTCTTGTGATAGCAGCGGACCGTTTGTGTATCCGGTGGGGCCGCTACTCAATCTTGAAGATGGTGCCGGAAAACAGTTGGACGATGAAGTGAAGAGGTGGTTGGACGGTCAACCGCCGTCCTCGGTGGTGTTCTTGTGTTTTGGGAGTATGGGAAGTTTCGAGGAGGTCCAAGTGAAGGAGATAGCGTATGCTCTCGAGCGGAGCGGCCACCGTTTCTTGTGGTCCCTACGTCGACCTCCGTTGGCAGAAAAAGGATTTGGGAATCCAAGTGATTACGAGGATCCACGAGTGGTGTTGCCGGAAGGATTCATGGACCGCACTGTCGGAATCGGAAAAGTGATCGGGTGGGCTCCACAGGTGGCGTTGCTGGCTCACCGTGCTATCGGGGGGTTTGTGTCCCACTGTGGATGGAACTCCGTGTTGGAGAGTTTATGGTTCGGTGTACCGATTGCGGCATGGCCAATGTACGCCGAGCAACAATTGAATGCATTTGCAATGGTGGTGGAGCTGGGATTGGCGGTGGAGATTAAGATGGATTACAAGAAAGATATGTTTAATCCTAAGGCCGAGATTGAGTTAGTGACGGCGGATGAGATCGAGGGCGGCATAAGACGGCTGATGGCGGATGAGACCATCAGAACACGAGCAAAAGAGATGAGCGAAAAGAGCAGAGCAGCGGTGGCGGAAGGCGGTTCTTCGTATGCTTCTGTTGGATCTCTTATCGAGGATTTTATGAGAAACATCTCATGA |
| ***CtUGT52*** | ATGGAGAAAAGCTACAAAGGTCATGTTTTGGTGTTACCTTATCCAAGCCAAGGCCACATCAACCCCCTCCTCCAATTCGCCAAAAGGCTCGCATCCAAAGGCCTCAAAGCCACCATCGCCACCACCCACTACACCCTCTCCTCCATCTCCGCCCCGTCCGTCGCGGTCGAACCCATCTCCGACGGCTTCGACCACGGCGGCTACGCCCAGGCCCAATCCGAAAAGCTCTTCCTCGACTCCTTCAAATCCAACGGCTCGCGTACGCTCGACCAGCTCATCAAGAACCACCGGACCACCGACCACCCGATCACTTGCATCGTCTACGATTCCTTTCTGCCTTGGGCCCTTGACGTGGCCAAGGAGAATGGGATCCTCGGGGGCCCGTTTTTCACGAACTCGGCCGCGGTGTCGGCCATTTTCAGCCGGATATACGCAGGGACGTTGAGGTTGCCGGTGAGAATGGAGGATTGTCCGGTGGTGCTGCCCGGGATTCCGCCGTTGGATTTGGAAGACTTGCCAAGCTTTTTGAATGCGCCGGAAAGTTATCCGGCGTACTTGGAGATGAAGCTGAATCAGTTTTCGAATCTGGAGAAGGCTGATTGGGTTTTCAGCAATACTTTCCAAGCATTGGAAGATGAGGTGGTGCAAGGATTGGAGGAGCAATGGCCAGCAAAACTAATAGGTCCAATGGTGCCATCAGCCTACTTAGATGAAAGAATTGAAGGTGACAAAGGGTATGGTGCAAGTCTATGGAAACCACTTGGCCATGAGTGCACCAAATGGCTTGAATCCAAGCCCCAAAACTCAGTTATCTACATTTCCTTTGGGAGCATGGTGTCCTTAAGCCAGCAAGAAATGGAAGAAATAGCATGGGGTTTACAAAAAAGCGGGTTTGACTTTCTTTGGGTAGTCAAAGATACAGAGAGACACAAGTTGCCAAAAGGGTTTCTTGATTTCATCACACAAAATCAAGAAAAGGGTATGATAGTAAATTGGTGCAACCAATTAGAGATACTAGCTCGTAAATCAGTGGGTTGTTTCGTGACACACTGTGGGTGGAACTCGACACTGGAAGGGTTGAGTTTGGGTGTGCCGATGGTCGGGATCCCAAAGTGGGCTGACCAATTGACTGATGCCAAGTTTATAAAAGATGTGTGGTGTGTGGGTAGTAGGGTCAAGGTGGATAGTGAAGTAGGGATTGTGAAAAGGGAAAATGTTATTATGTGTTTGAATGAAGTGATGAAAGAAGGAAAGAGGGGTTTAGAGATCAAGAAAAATGTGGGGAAATGGAGGGAGATGGCTAAAGAGGCAATTAGTGAAGGTGGGAGCTCAGATAAAGCAATTGATGAGTTTGTAGTGGCATTGAAGACATTTGCAAGAAAAAATAAC |
| ***CtUGT53*** | ATGGTATATGAAGTCAATGAATACATGGATAAGAAAGAACATATTGCGATTTTTACCACAGCTAGCCTTCCATGGATGACTGGAACCTCTGTTAATCCTCTATTCCGTGCAGCGTATCTTGCCAAAGATGGACATAGAAAGGTTACTTTGGTCATTCCTTGGCTATCGAAGAGCGATCAAGAATATCTATATCCCAACAAAATAACATTTAATTCGCCCAAGGAACAAGAGAAATATGTCCATGAGTGGATTGAGCAAAGGACTGAGTTTTTGCCTAGTTTCAATATACGTTTTTATCCAGGGAAGTTTTCTAGAAGTAAAAGAAGCATTCTTGCTCTTGGGGATATAACGGAAAGCATTCCGGATGAAGAATCTGATATTGCCGTCCTTGAGGAACCCGAACATCTAACGTGGTACCACCATGGTAAAAGATGGAAGATCAAATTCCGCCTTGTTATAGGAATTGTTCACACGAACTATTTGGAGTACGTCAAAAGAGAGAAAAATGGACGTGCCTATGCCTTTCTTCTCAAGTACATGAATAATTGGGTGGTCGATATATACTGTCATAAGGTAATACGATTATCTGGAGCAACACAAGAACTCCCAAGATCCGTTATTTGCAATGTTCATGGCGTTAATCCTAAGTTTCTGGAAATCGGGATGAAAAAGAGGGAAGAACAGAAGCTCGGAAAGCAGGCGTTTACGAAAGGTGCATACTTTATTGGGAAAATGGTGTGGAGCAAAGGCTACACGGAGCTGCTTAAACTTCTTCGTGATCACCAAAAGGAACTTGAAGGACTTGAGGTTGATTTATTTGGTACCGGTGAGGATTCTGCTGAAGTACAAGAAGCTGCAGAAAAGTTGAATTTAACCATCAGGGTTAATCCAGGACGCGATCACGCAGACCCTTTATTTCACGATTACAAAGTGTTCCTGAATCCAAGCACCACAGATGTGCTCTGCACAACCACCGCCGAAGCTCTTGCAATGGGCAAAATCGTCCTTTGCGCAGATCACGTCTCTAACGAGTTCTTCAAACAGTTTGCCAATTGCCGAACCTTCAAGGACGGAGAGAGTTTCGTTAATGTCACCCGCCAATCGTTGACCGAACAGCCGGCCCCACTGACCAATTCGGCAATGCACGAGCTTTCATGGGATGCTGCAACCTCGAGGTTTCTAAAAGCGGCTGAACTCAATAAGACTCCCGAGAAGAAACTAACGAAATCACATTCCAAGAGCTTTTTGTCATCGTCATTGAGTTTTCAACGGAATCTCGAGGACGCGTCTGCGTTTATGCATTTCGTTGGAACCGGTTTCGTGAGTCCAGTGCCGAACGAGCAACAATGTGAAGAGCTCGGGCTGAACGTCCCAAGTAAAAGATTCGGATATCCACGTTCAAAACACGCATCT |
| ***CtUGT54*** | ATGGAGGAGAAAGTAGAGGTGTTTTTCATCCCATCGCCACTTATGGGACATGTTGGCCAGATGGTTCAGCTGGCCAACCTCATGGTCACCCGGTTTCACCATCTCACCATCACCATACTCGTCATGCACCTCCCCACCGACCCCATCGGCACCGATTACACCAACTCCCTCGACGACCACCACGATCGAATCAAATTCATCCAATTCCCTCCGATGGATCCCGACTCTTTTCCGGATTGCCCGACTGTCGGTTTCATGGCCGATGCCATCATTGAACGCCATAAGCCCATCGTCGGAGAACTCGTGGCTGCTCGCTTCAACGGGTCCAATCGCACCCCTCGACTCGGCGCCTTGGTCGTTGACATGTTTTGCACGCCGATGATCGATGTCGGCAAGGAATTCGGTGTCCCCACCTACGTGTTCTTCACCTCAAATGCGGCTTTTCTCGGGATTATGTTATATTTCCAGACCCTTGAAGACGAACACGGCCAGGAGACACCCGAATTGGCGAATCCGGGTCCTCCGTTGATCATCCCGAGCTATGCTGAACCGGTTCCACCAAGTGTCTTGCCCTATGTGCTTTCGGACCAGGACACTTGGTACAAAAGGTTTATTCGTTATACCCGAAAATACAGAGAAGCCAAGGGTATAATCGTAAACACGTTTCGAGAGTTAGAGCCTCATGCGCTCCTTTCCTACGACGATAAAACACCACCTGTTTACACGGTGGGTCCCATGCTAAAACCCGAAAAGCCTACACCAAACAACGAGTTGCTTCAGTGGTTGGATGGTCAACCGAAGTCATCGGTCTTGCTCCTATGCTTCGGATCCCGGGGGTGGTTCGAGGTGGACCAAGTGAAACAAATAGCGATTGCTATAGAAAGGAGTGGATACAGGTTCGTATGGTCCCTACGCCAACCTCCAACCGAGAACCAAAAAGGGTTCCCAAGGGAGTACACAGACTACAACGAAGTCCTGCCAGATGGGTTTCTTGACCGTACAGCTGGAAAGGGCAAAGTGGTTGGGTGGGTCCCGCAAACGGCATTATTGGCTCATGTGGCAGTTGGTGGGTTCGTATCCCACTGTGGGTGGAACTCCATACTGGAGAGCCTCTGGTACGGAGTTCCAATTGCCACATGGCCAATATACGCGGAGCAACAGTTAGATGCGCATCAATTGGTTAAAGAACTGGGTTTGGCAGTCGAAATCTCGTTGGATTATAACCAATTAAACAAGAATCAAAGGTTGGTGTTGGCTGAAGAGATTGAAAAGGGAATACGGCAGGTGATGGATAGCAATAGTGAGGTTCGAGCAAAGGTGGCACAAATGAAAGCAAAGAGCCGAATGGCACTCGAAGAAGGTGGTTCATCGATTAACAGTTTGAAAGACCTTGTAGACGATTTCATG |
| ***CtUGT55*** | ATGCCGACCGCCACCGTCCGCCACCACCAGCCACCGCATATCGCCCTCTTCTCGAGCGCCGGAATGGGCCATTTGACCCCACTCCTCCGCGTTGCTTCCATGCTCGCCTCCCGCAGCTGCCACGTAACTCTCGTCACCGCCGAACCCGCCGTCTCCGCCGCGGAAACCGCCCACATCACCGCCTTCCTGGCGGCGTATCCCGCCGTCAACCGCCTGCCCTTCCGAACCCTTCCGTTCACACCGCCAGCCACCGCTGACCCTTTCTTCGTCCAATTCGAAGCCATCAACCGCTCCGTCCACCTCCTCGCCCCGACCTTGTCCTCCGCATCGCCGCCGGTCTCCGCCGTCTTCTCCGACATGGCGTCGGCTGCCGGGGTCCGCCGGGTGGCCGACGAGCTTCGGGTCCCGATCTACATCGTCTCGACCACCTCCGCCAGGTTCACGGCCCTGGTGGCGAGCATCCCGGCCCTGATCGGGGCCGGAAGCTCCATAACAGCGACGGCGGAGGGAGCTTCCTCCGCCGTCTTTGGGATCCCTGGTCTGGATCCGTTTGAGATCTCGGCGCTTCCGCCGGCGCTGTTCGTACCGGATAACCTTTTCACGAAGACTTTGGCTGCGAACGCGCTTGCGATGAGAAAGGCGAAAGGCATTTTGACGAACACGTTCACTACATTCGAACCGGAAACGATTGCAGCGGTTAACGGCGGCAAATCTTTACCGGATTTCCCGCCCCGGACCGCCCTGTCGCAACCCCAAATCGTCGAGCTCCGAAACGGGCTGGAGGAGAGCGGGCGGAGCTTCTTGTGGGTCTTCAAGTCCACGGTCGTCGATAGAGACGACACCGGATCCGACCTCGGGGAATTGCTCGGCGGGGATCCGACGAAACCGTCGAACGGGATGGTGGTGAAAGGGTGGGTGAACCAAGAGGCGATCCTGTCGCACCCCGCGATCGGGTGCTTCGTGAGCCACTGCGGGTGGAACTCGGCAGTGGAGGCCGCGGCGGCCGGGGTGCCGGTGGTGGCGTGGCCGCTGGCGGGGGACCAGAAGGTGAACGCGGAGGTCGTGGCGGGGGCGGGGTTGGCGCGGTGGGAGAAGGGGTGGGGGTGGATGGGGGAGAGATTGGTGAAG |
| ***CtUGT56*** | ATGGCGGACTCCATCACCTCCTCCCAGTCCCATGTGGTGGCGATACCATACCCCGGCAGAGGCCACATCAACCCAATGCTCAACCTCTGCAACCTCATGTCCCTCCGCCGCCCTTCCGACCTCCTCATCACCGTCGTCGTCACCGAAGAATGGCTCGGATTCATCGGATCCGACCCGAAACCGACAAACGTCCGCTTCGCCACCATCCCTAACGTCATCCCGTCGGAGCTCGACCGCGCCTCCGACTTCGCCGGCTTCATCAAATCCATTCACACAAAACTAGTAGACCCGGTCGAGAGATTACTCCGCCGGATGGAAATTCCGGCGACCGTAATCATATACGATACCTACCTCATGTGGATCATAGATCTCGGAAAACGGTTGAACATTCCGGTGGCTTCCTTCTTCACGATGTCGGCCACGGTGTTCTCCATGTGTTATCATCACGATCTCCTCCTCCAAAACGGCCATGTCGGAGATGATTATTTCTCAGAAAAAGGTGAGGAAGTGATCGATTACATACCTGGAGTGCCTCCCATGCGCGTGGCTGATCTCGTGACAGGCTTCAATGGCAAAGGAAAAGAGGTTTTTCCGTTAGCTCTGCAAGCTATTTTAATGGCGGACAAAGCTCGGTTTCTGCTTTTCGTGTCGGTTTACGAGTTGGAAGATAAAGTGATCGATGCCTTAAAATCGGAGCTTTCGGTGCCCGTTTACGCTATTGGGCCGTCCATACCCTACTACTTCAATGTCCAAAATGACCAAAATACCCCTGACTATCTGGAATGGTTAGACCGTCAGCCGGAGGCCTCGGTGTTGTACATCTCGCAAGGGAGTTTTCTCTCGGTCTCGAATGCGCAGTTGGAGGAGATCGTCGCGGGCGTGCATGAGAGCGGTGTACGGTACGTGTGGGTTGCACGTGGCGAGACGTCTCGGTTTGGACGCGAAAATGACGAAAGTGGGCTTGTCATACCTTGGTGTGACCAATTACGGGTGCTGTGTCATGGTTCGGTAGGGGGATTTTGGTCACACTGCGGGTGGAATTCGACGAAAGAAGGTGCGTATGCGGGGGTGCCGATGCTCACGTTTCCCATATCTATCGATCAAGTTCCGAACAGTAAGATGATCGTTGAAGATTGGAAAACGGGAAGGAGGGTGAGCGTTGACGAGGGTGTTTTGGTCACTCGAGACGAAATCGCAAAACTCATAAAGGGTTTCATGGACGAGGAGAGCGAAGAAGGAAAAGAGATGCGCAAAAGGGCAAGAGAAATCAAGAAAATCTGTCGACACGCGACCGATGAAGGAGGGTCCGCTCAGAAAGATATCGATTTATTCATCGATGACATTTTGATCAGTCGAAACAAT |
| ***CtUGT57*** | ATGGAAGAAGGTGGAAGACGACTGGTGGTCCTAACTTCATCCCCATTTCATGGCCACATGACTCCAACTCTCCAGCTAGCCACCGCCCTTCATGCCAAAGGCTTCTCCATAGCCATAGCTCACTCCACCTTAAACCCTCCTCACCCATCCAATCACTTCACCTTCCTCCCGCTTTCCGACAACTTATCCGCCATCGATGCCTCCTCAAGCTTCACCGGTTTCGTCCAAACCCTCAACGCCAACTGCCGACCATCATTCCGTCAACACTTGGTTCGGTTGATCGCCCAAGGAGATTATGAATCGATCGTTGTCATCTATGATTTCCTTATGTTTTTTGCAGGAGGGGTTGCCGTCGATCTGAACCTTGGTTCGATCATCTTTCGTAGTAACAGTGCTACGTACTTTACGGCTTTTCTTGCCCGTCGGCAGCTGATTCAAAAAGGCCGGTACAAAGATCTGCCATTTTCGAAATCGCCTATCGAAGATTGGCACCAACTGCTTGCTATCTTCAGCCAACAAAGCAACCCCTCCGCAGTTATCTGGAACACTCTCGAATTTCTCGAACACGAAGCCTTATCCCAAATCCACCAGCACTACAAGGCTCCGGTCTTCGCAGTCGGACCTCTTCACAAGATAACGCCAAGTCCACCTACTAGTTTTCTCGAAGAGGACACCCGCTGCATAGCGTGGCTAGATAAACAAGCCCCCAAATCCGTGGTTTACGTAAGCTTTGGAAGTCTAGTTAGCGTAGATGCAAAAGTGTTGGTCGAGATGGCATGGGGTCTGGCCAAAAGTAAGCAGCCGTTCCTATGGGCGGTTAGGCCTGGTTCGGTTAGGGACTCCGAATGGAGCGAGTTCTTGCCGGATGGTTTTTGGGAAGAAACAAGCGGACGAGGTCTAGTTGTGAAATGGGCGCCCCAAAAGGAAGTTTTGGCGCATTCCGCGGTCGGTGGGTTTTGGAGTCATTGTGGTTGGAATTCGACGTTGGAGGGTATTTCGGAAGGGGTTCCGTTGATATGCCAACCGATTAACGTAGACCAAGGGGTGAACGCGCGATACGCGAGTTACGTGTGGAAGATAGGGGTGGAGTTGGAGGTTTTGGAGAGAGGGGAGATGGAAAGCATGATCAAAAGAGTTATGGTGGATGAAGAAGGGAAGGAGATGAGATTGAAAGTAGCTAAAATGAAGGAAATGGTTAAAGATGCAGTGAAAAATGGAGGTTCTTCCCATGATTCATTGGAGAGTTTAGTG |

Table S4. Amplification primer sequences of *CtUGTs* recombinant plasmid.

| Primer | Sequence(5'-3') |
| --- | --- |
| pET28a-*CtUGT50* F | ATGGGTCGCGGATCCGAATTCATGAATCAAGTCGTAATGATACCGTT |
| pET28a-*CtUGT50* R | GCAAGCTTGTCGACGGAGCTCTTTCTTTCGGTCCGTTACAAACT |
| pET28a-*CtUGT51* F | ATGGGTCGCGGATCCGAATTCATGCCGAATACAGTCGCAGAG |
| pET28a-*CtUGT51* R | GCAAGCTTGTCGACGGAGCTCTGAGATGTTTCTCATAAAATCCTCGA |
| pET28a-*CtUGT52* F | ATGGGTCGCGGATCCGAATTCATGGAGAAAAGCTACAAAGGTCATG |
| pET28a-*CtUGT52* R | CAAGCTTGTCGACGGAGCTCGTTATTTTTTCTTGCAAATGTCTTCAA |
| pET28a-*CtUGT53* F | ATGGGTCGCGGATCCGAATTCATGGTATATGAAGTCAATGAATACATGG |
| pET28a-*CtUGT53* R | GCAAGCTTGTCGACGGAGCTCAGATGCGTGTTTTGAACGTGG |
| pET28a-*CtUGT54* F | ATGGGTCGCGGATCCGAATTCATGGAGGAGAAAGTAGAGGTGTTTTT |
| pET28a-*CtUGT54* R | GCAAGCTTGTCGACGGAGCTCCATGAAATCGTCTACAAGGTCTTTCA |
| pET28a-*CtUGT55* F | ATGGGTCGCGGATCCGAATTCATGCCGACCGCCACCGTC |
| pET28a-*CtUGT55* R | GCAAGCTTGTCGACGGAGCTCCACCTTATGAGAGCTCCCTCCC |
| pET28a-*CtUGT56* F | ATGGGTCGCGGATCCGAATTCATGGCGGACTCCATCACCTC |
| pET28a-*CtUGT56* R | GCAAGCTTGTCGACGGAGCTCATTGTTTCGACTGATCAAAATGTCA |
| pET28a-*CtUGT57* F | ATGGGTCGCGGATCCGAATTCATGGAAGAAGGTGGAAGACGACT |
| pET28a-*CtUGT57* R | GCAAGCTTGTCGACGGAGCTCCACTAAACTCTCCAATGAATCATGG |
| pMT39-*CtUGT52* F | CCACCGCGGCCGCCACCATGGATGGAGAAAAGCTACAAAGGTCATG |
| pMT39-*CtUGT52* R | CCTCGCCCTTGCTCACCATGGGTTATTTTTTCTTGCAAATGTCTTCAA |
| *CtUGT52* qF | GTGAGAATGGAGGATTGT |
| *CtUGT52* qR | CTTGGAAAGTATTGCTGAAA |
| *CtUGT52*-ovx F | CGACAGTGGTCCCAAAGAT |
| *CtUGT52*-ovx R | CTTGGAAAGTATTGCTGAAA |

Table S5. Primer sequences for site-directed mutagenesis and recombinant plasmid amplification.

| Primer | Sequence(5'-3') |
| --- | --- |
| *CtUGT52*Y21 F | AGCCAAGGCCACAT*CT*ACCCC*CT*C*CT*CCAATTCGC |
| *CtUGT52*Y21 R | TAGATGTGGC*CT*TGG*CT*TGGATAAGGTAACAC |
| *CtUGT52*Q142 F | TTCAGCCAGATATACGCAGGGACGTTGAGGTT |
| *CtUGT52*Q142 R | GCGTATATCTGGCTGAAAATGGCCGACACCGC |
| *CtUGT52*H162 F | TGGTGCTGCACGGGATTCCGCCGTTGGATTTG |
| *CtUGT52*H162 R | AATCCCGTGCAGCACCACCGGACAATCCTCCA |
| *CtUGT52*A213 F | TTGGAAGATGCGGTGGTGCAAGGATTGGAGGA |
| *CtUGT52*A213 R | ACCACCGCATCTTCCAATGCTTGGAAAGTATT |
| *CtUGT52*E217 F | GGTGCAAGAATTGGAGGAGCAATGGCCAGCAA |
| *CtUGT52*E217 R | CCTCCAATTCTTGCACCACCTCATCTTCCAAT |
| *CtUGT52*P221 F | AGCCATGGCCAGCAAAACTAATAGGTCCAATG |
| *CtUGT52*P221 R | TTTTGCTGGCCATGGCTCCTCCAATCCTTGCACC |
| *CtUGT52*F246 F | CAAAGGGTTTGGTGCAAGTCTATGGAAACCAC |
| *CtUGT52*F246 R | TTGCACCAAACCCTTTGTCACCTTCAATTCTTT |
| *CtUGT52*A247 F | AGGGTATGCTGCAAGTCTATGGAAACCACTTGG |
| *CtUGT52*A247 R | GACTTGCAGCATACCCTTTGTCACCTTCAATTCT |
| *CtUGT52*N277 F | CTTTGGGACCATGGTGTCCTTAAGCCAGCAAG |
| *CtUGT52*N277 R | ACACCATGGTCCCAAAGGAAATGTAGATAACTGAG |
| *CtUGT52*L332 F | TGTGCAACCAATTAGAGATACTAGCTCGTAAA |
| *CtUGT52*L332 R | CTCTAATTGGTTGCACAAATTTACTATCATACCCTTTTCTTGATTTT |
| *CtUGT52*F333 F | GGTTCAACCAATTAGAGATACTAGCTCGTAAA |
| *CtUGT52*F333 R | CTCTAATTGGTTGAACCAATTTACTATCATACCCTTTTCTTG |
| *CtUGT52*T334 F | TTGGTGCAGCCAATTAGAGATACTAGCTCGTAAATCA |
| *CtUGT52*T334 R | CTAATTGGCTGCACCAATTTACTATCATACCCTTT |
| *CtUGT52*P335 F | GTGCAACCCATTAGAGATACTAGCTCGTAAATCAGTGG |
| *CtUGT52*P335 R | TCTCTAATGGGTTGCACCAATTTACTATCATACCC |
| *CtUGT52*S336 F | GTGCAACCAATCAGAGATACTAGCTCGTAAATCAGTGGG |
| *CtUGT52*S336 R | TCTCTGATTGGTTGCACCAATTTACTATCATAC |
| *CtUGT52*G358 F | ACTGGGAGGGTTGAGTTTGGGTGTGCCGATGG |
| *CtUGT52*G358 R | AACTCAACCCTCCCAGTGTCGAGTTCCACCCA |
