# Supplementary figures and images for: BSA-seq integrated with transcriptomics and metabolomics revealing the candidate genes associated with safflower colors and flavonoid glycosides biosynthesis
Source: Hortic Res. 2026 Mar 4;13(6):uhag068. doi: 10.1093/hr/uhag068 (PMC13253347; doi:10.1093/hr/uhag068)

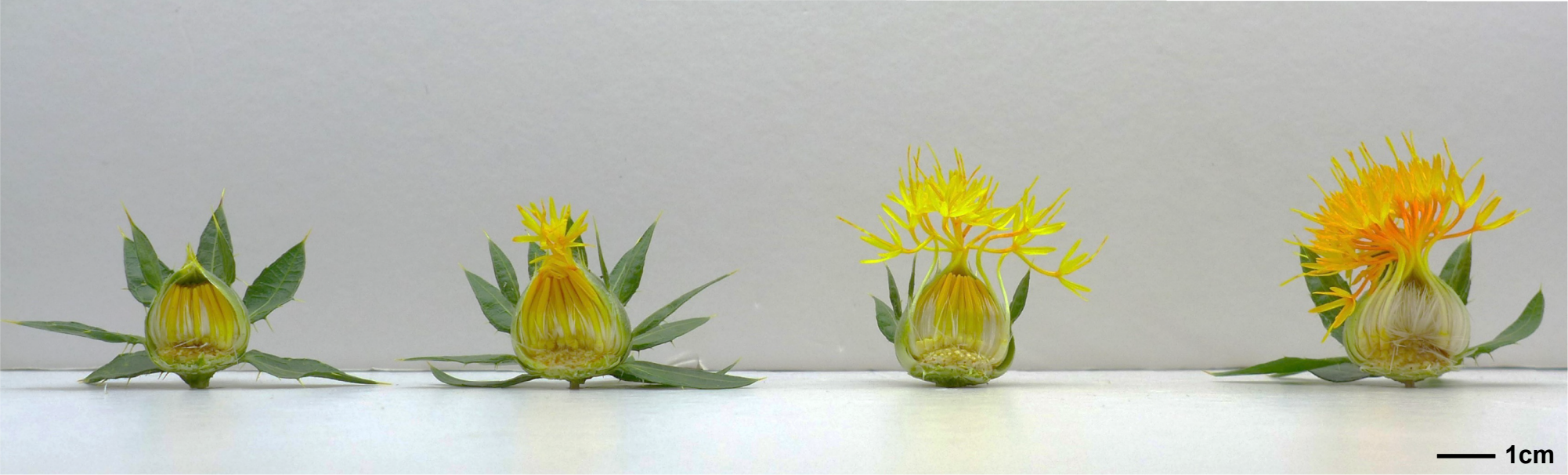

— 1cm

Supplement: Web_Material_uhag068 [file web_material_uhag068.zip › Figure S1. Morphological description of four different growing stages of yellow safflower (from left to right Y_â. - Y_â.£)..pdf]

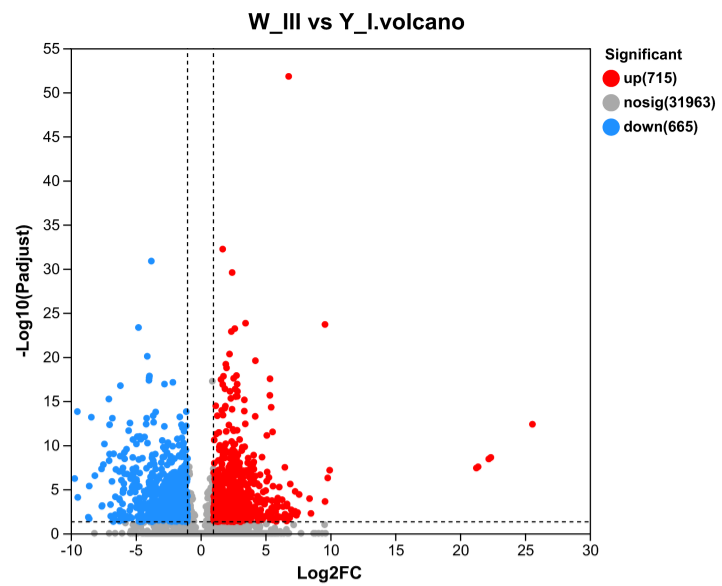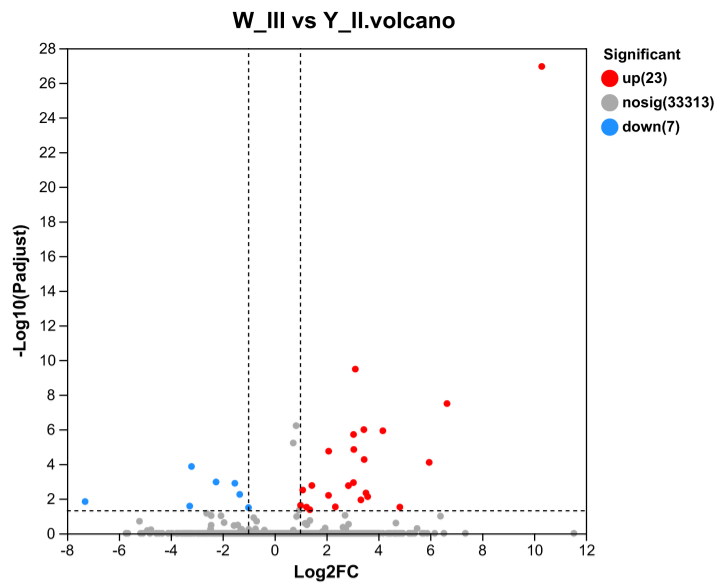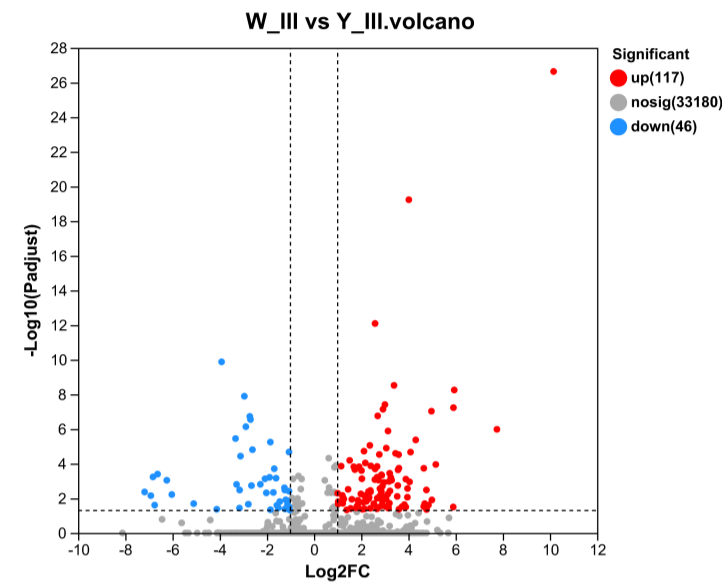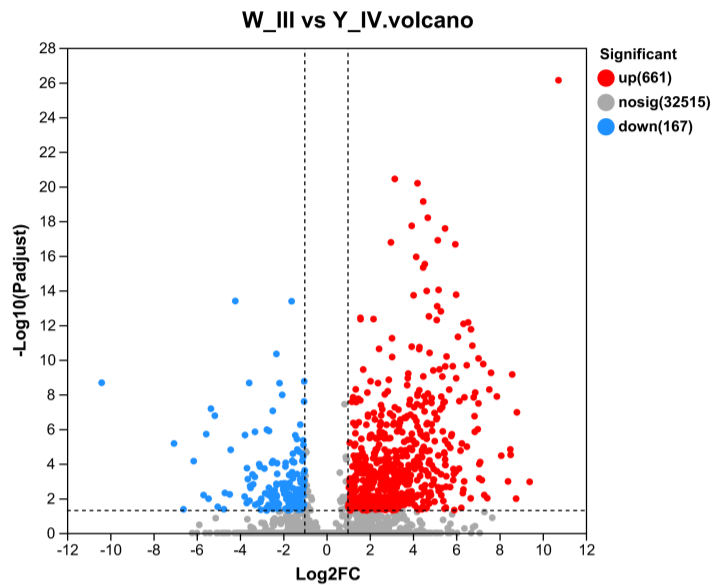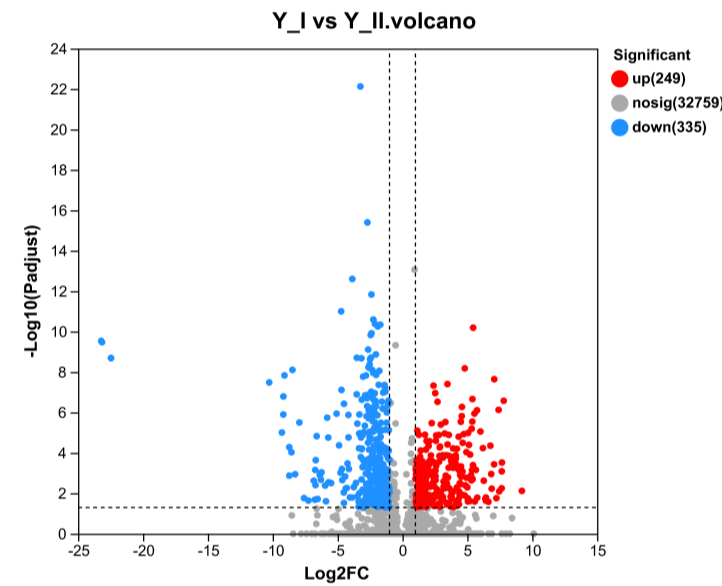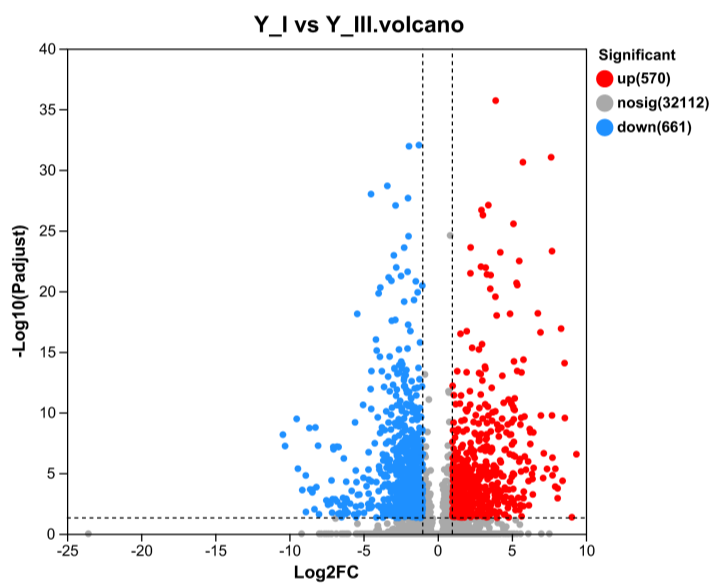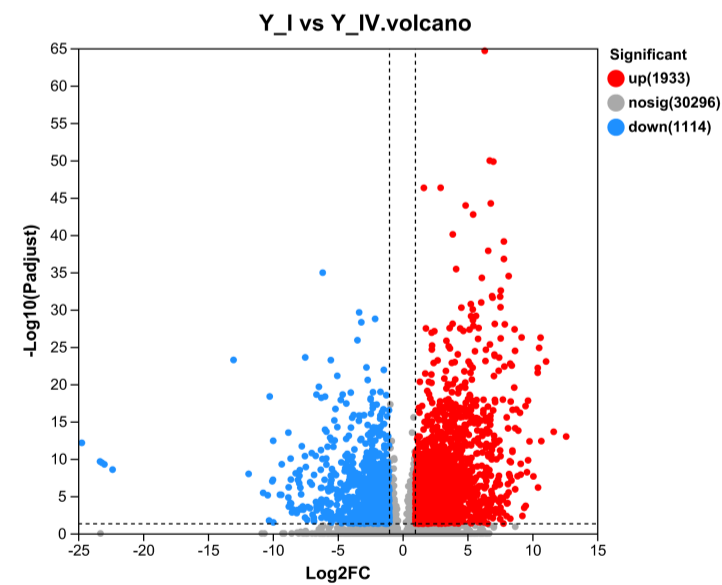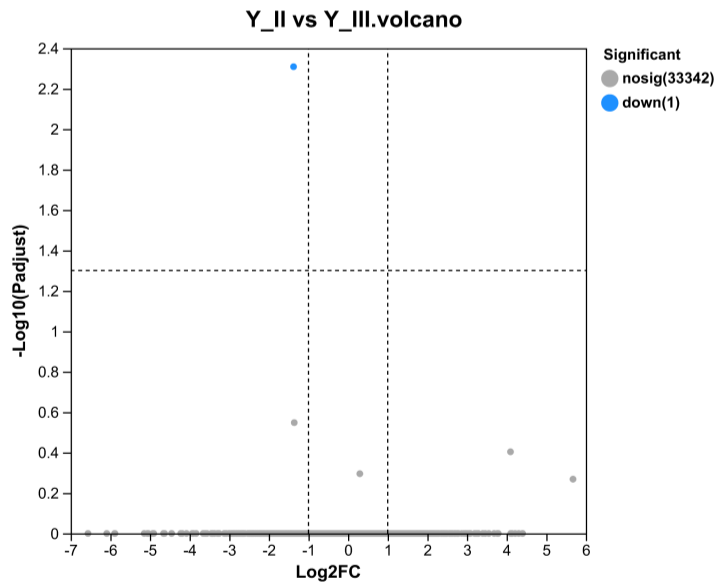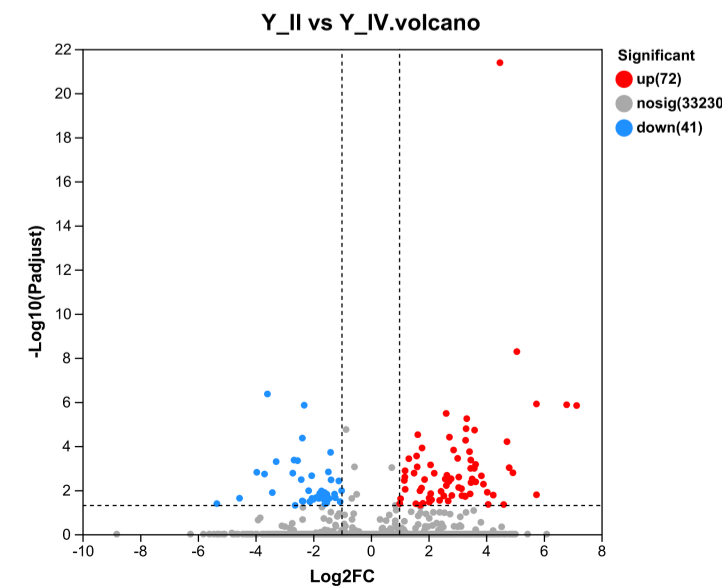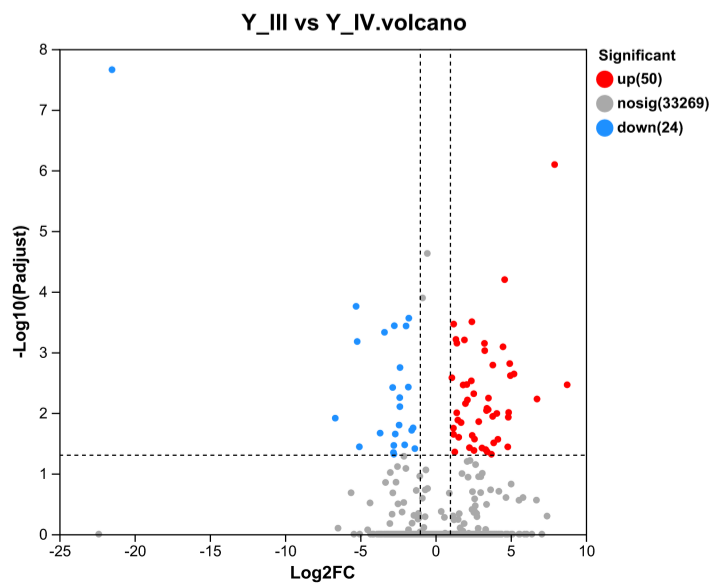

Supplement: Web_Material_uhag068 [file web_material_uhag068.zip › Figure S2. Volcano plot of differential expressed genes among different accessions and growing stages..pdf]

**a**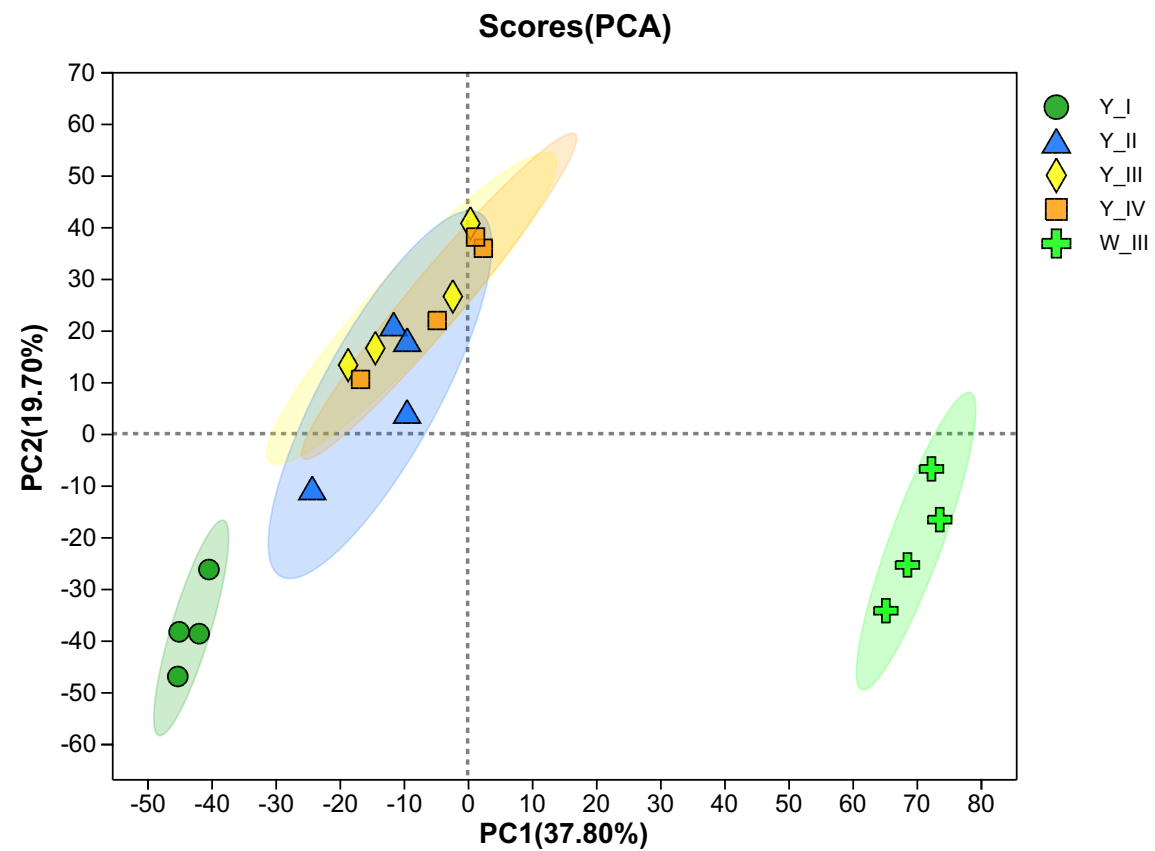**b**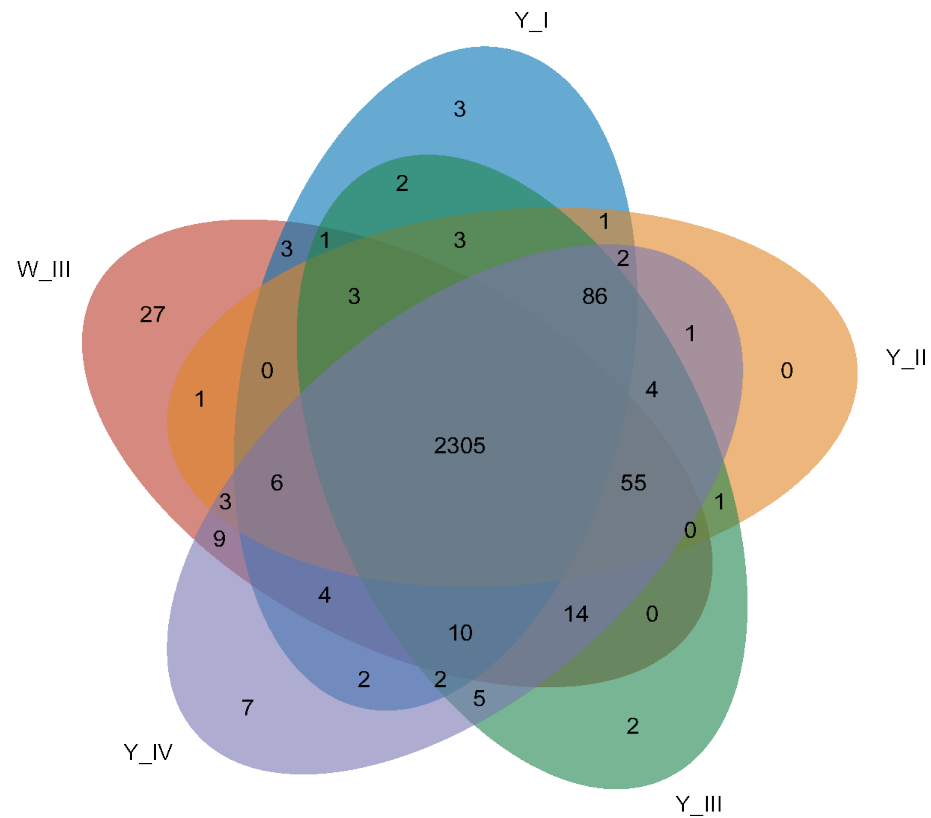

Supplement: Web_Material_uhag068 [file web_material_uhag068.zip › Figure S4. PCA and Venn diagram between different groups..pdf]

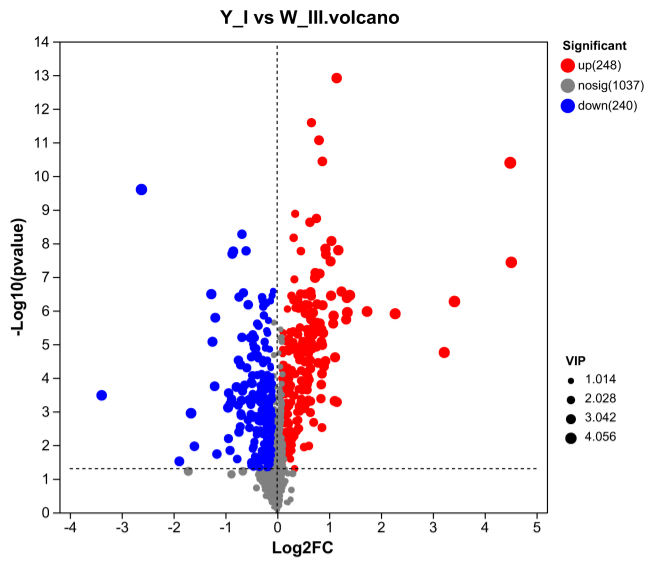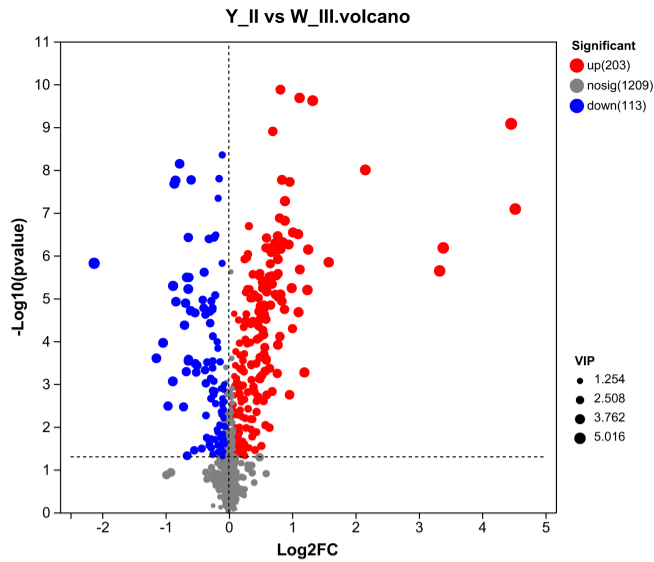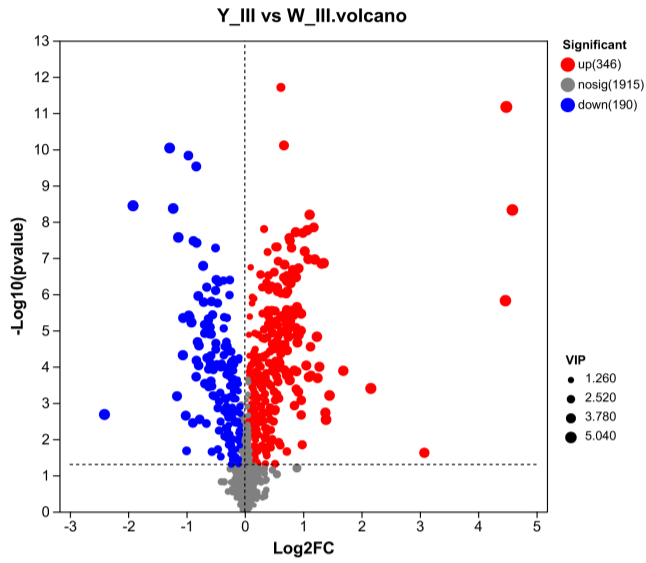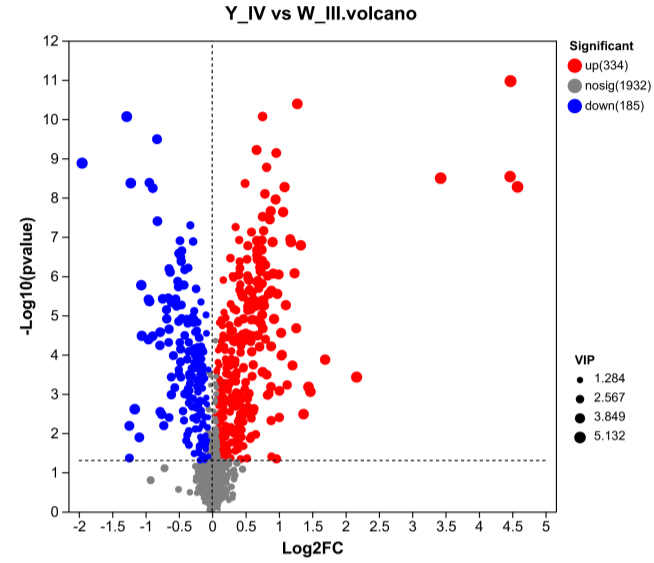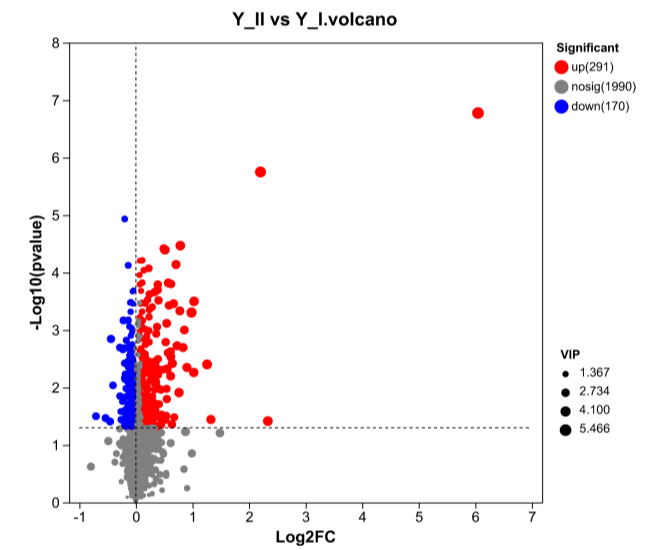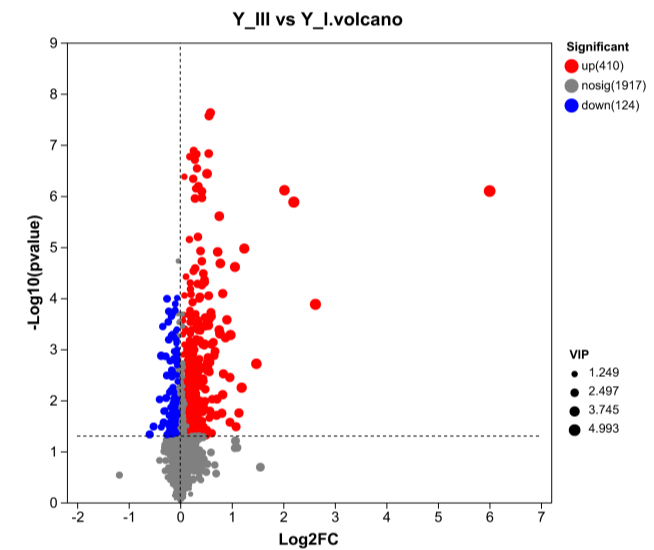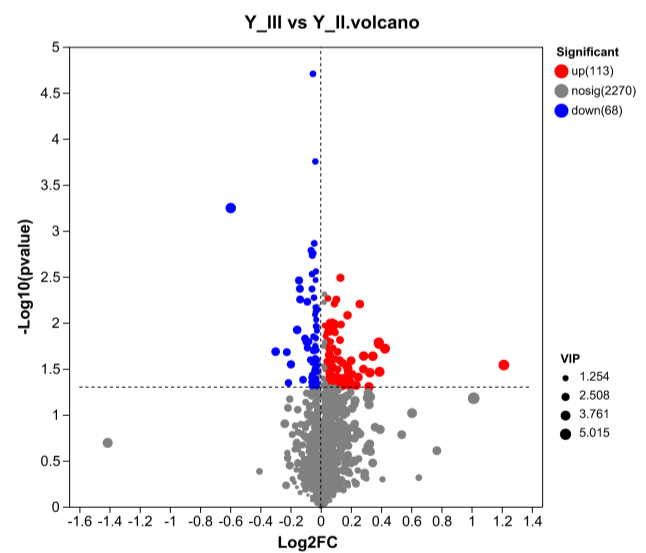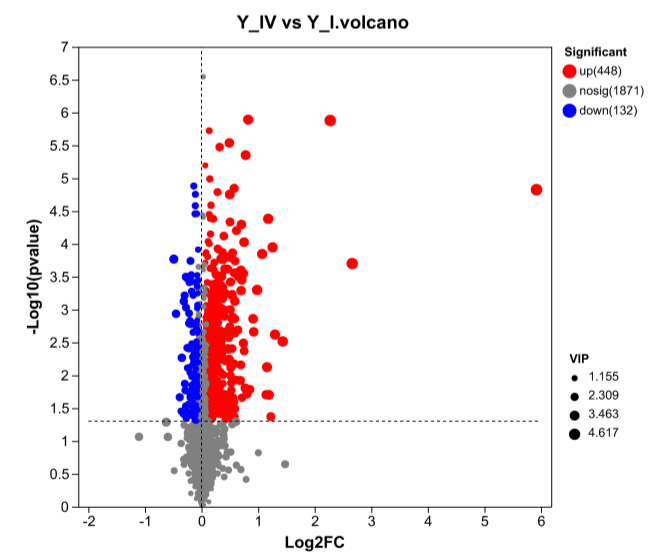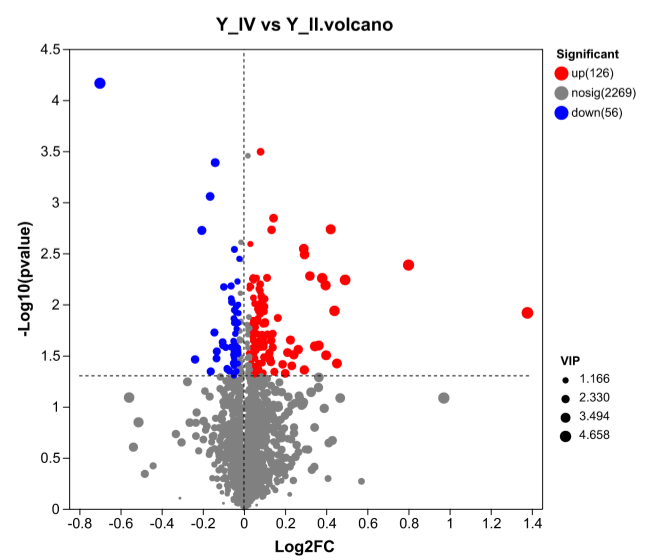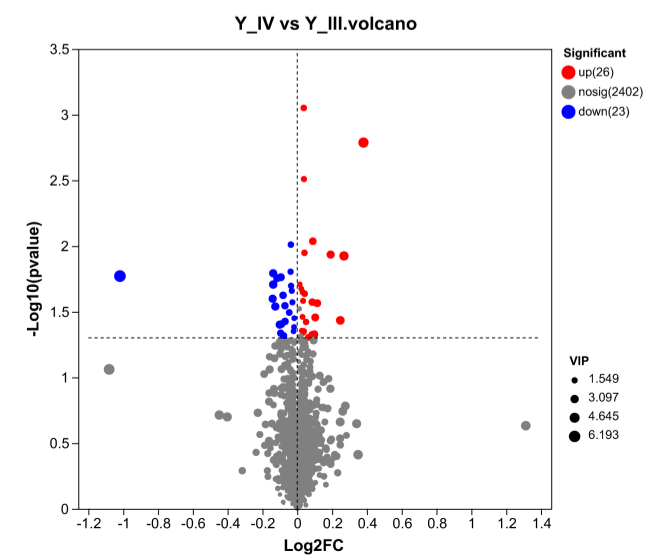

Supplement: Web_Material_uhag068 [file web_material_uhag068.zip › Figure S5. Volcano plot of differential expressed metabolites among different accessions and growing stages..pdf]

Metabolites Content (%)

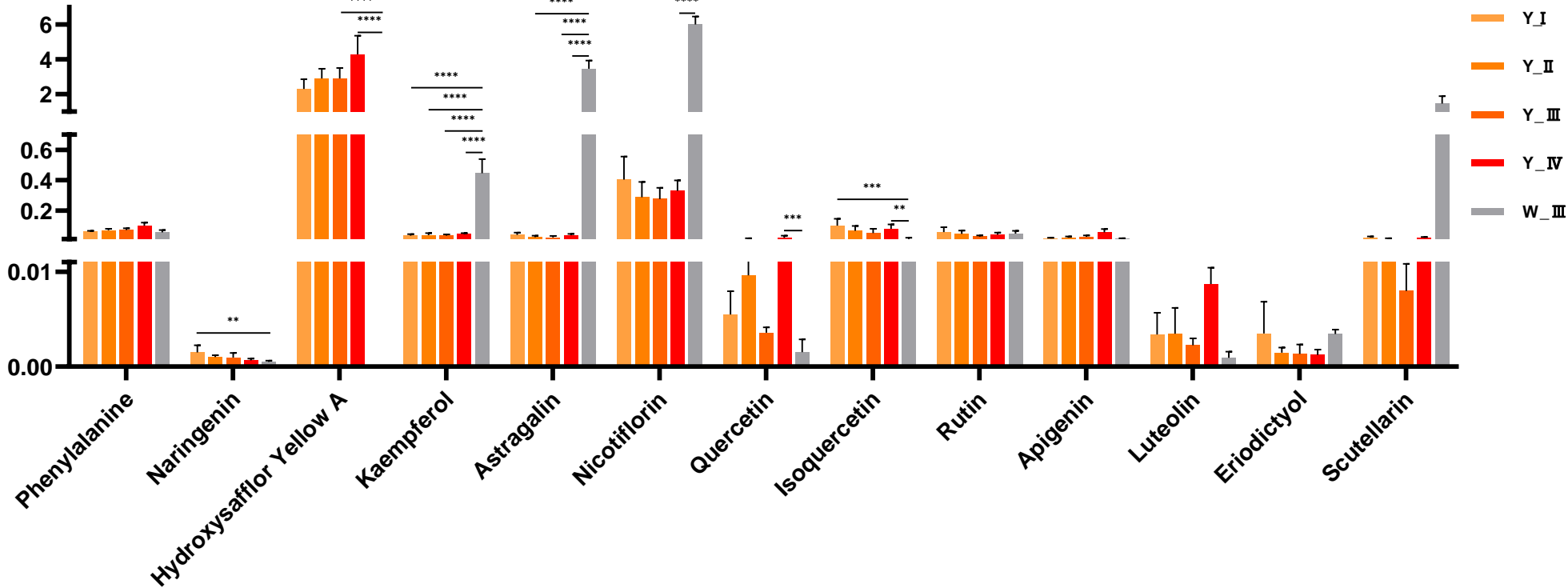

Supplement: Web_Material_uhag068 [file web_material_uhag068.zip › Figure S6. Analysis of targeted metabolites accumulation..pdf]

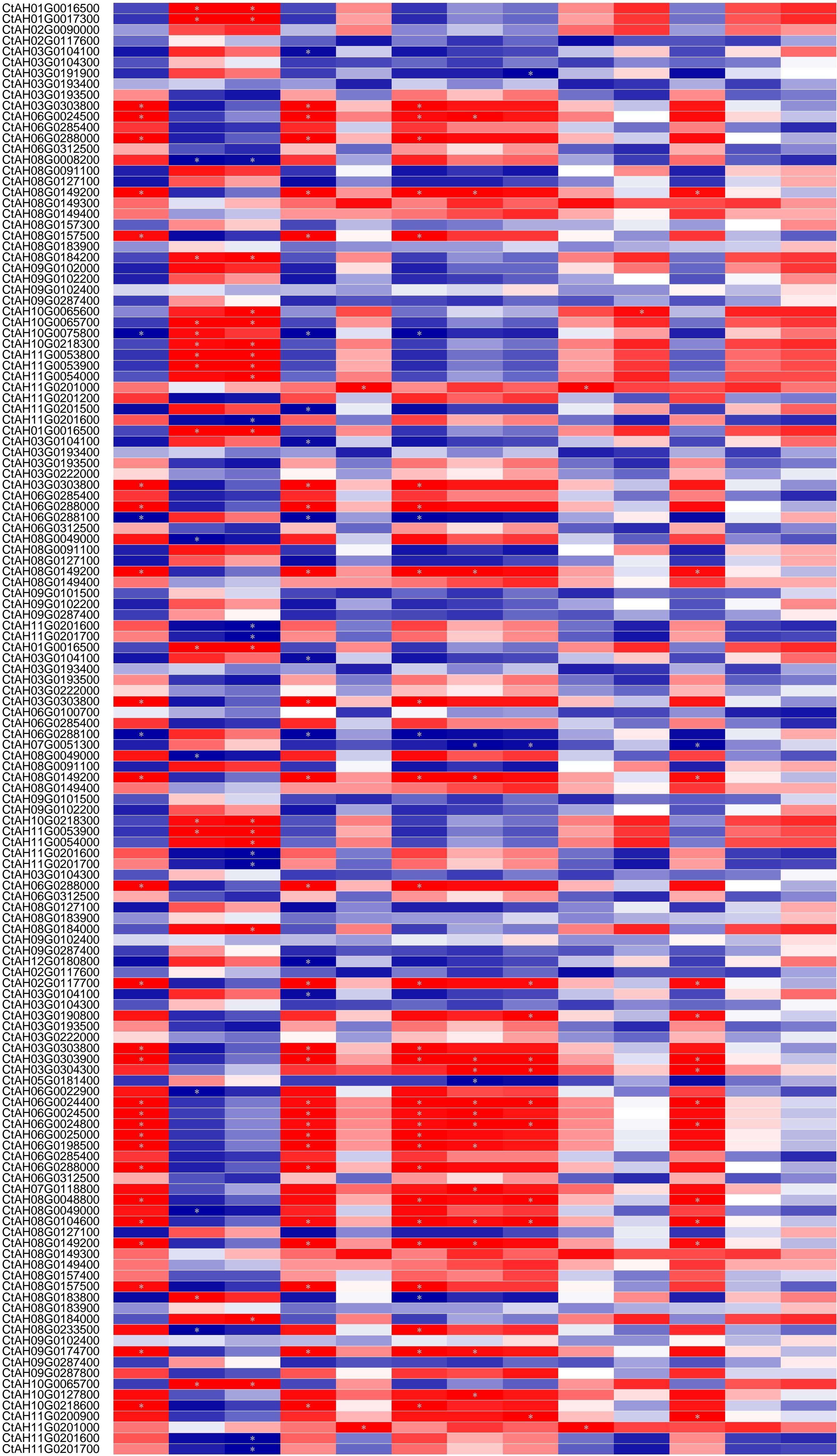

\* p<=0.05

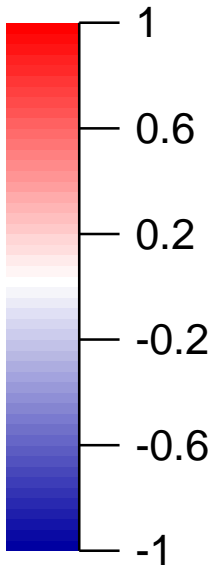

Supplement: Web_Material_uhag068 [file web_material_uhag068.zip › Figure S7. Correlation analysis of differentially expressed CtUGTs with targeted metabolites..pdf]

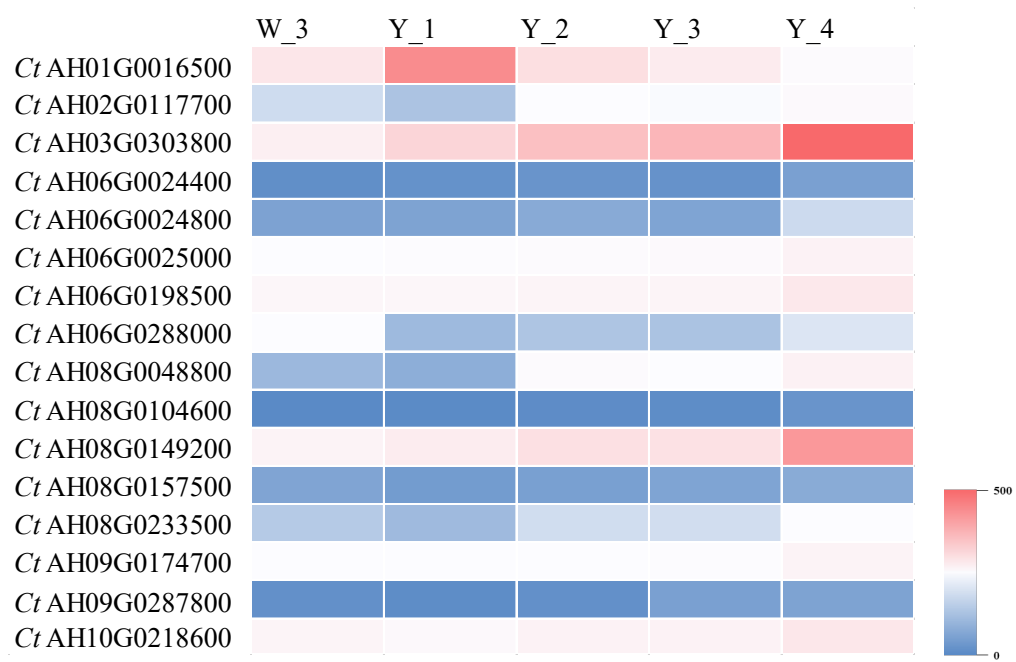

Supplement: Web_Material_uhag068 [file web_material_uhag068.zip › Figure S8. The expression profiles of the 16 screened CtUGTs..pdf]

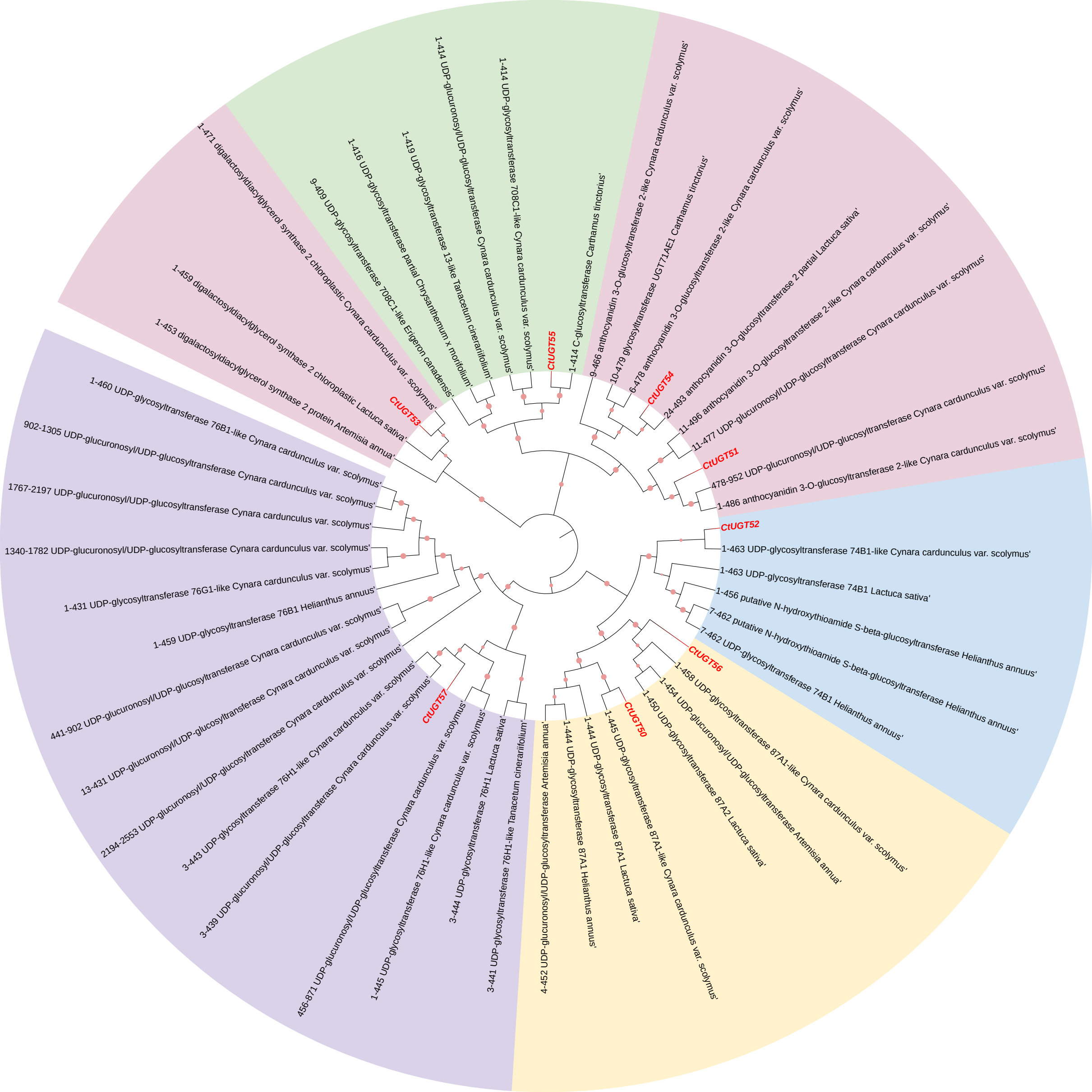

Supplement: Web_Material_uhag068 [file web_material_uhag068.zip › Figure S9. Phylogenetic analysis of target CtUGTs..pdf]

Relative mRNA level  
adjusted to 60S

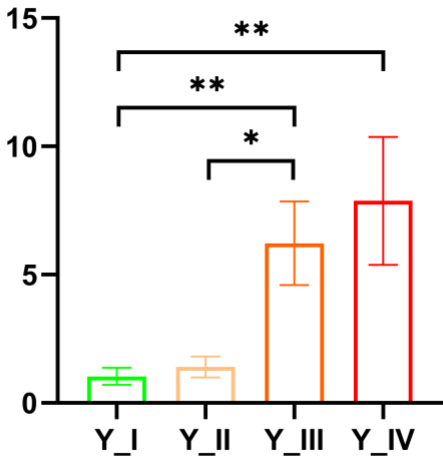

Supplement: Web_Material_uhag068 [file web_material_uhag068.zip › Figure S12. Relative expression Levels of CtUGT52 during different growing stages..pdf]

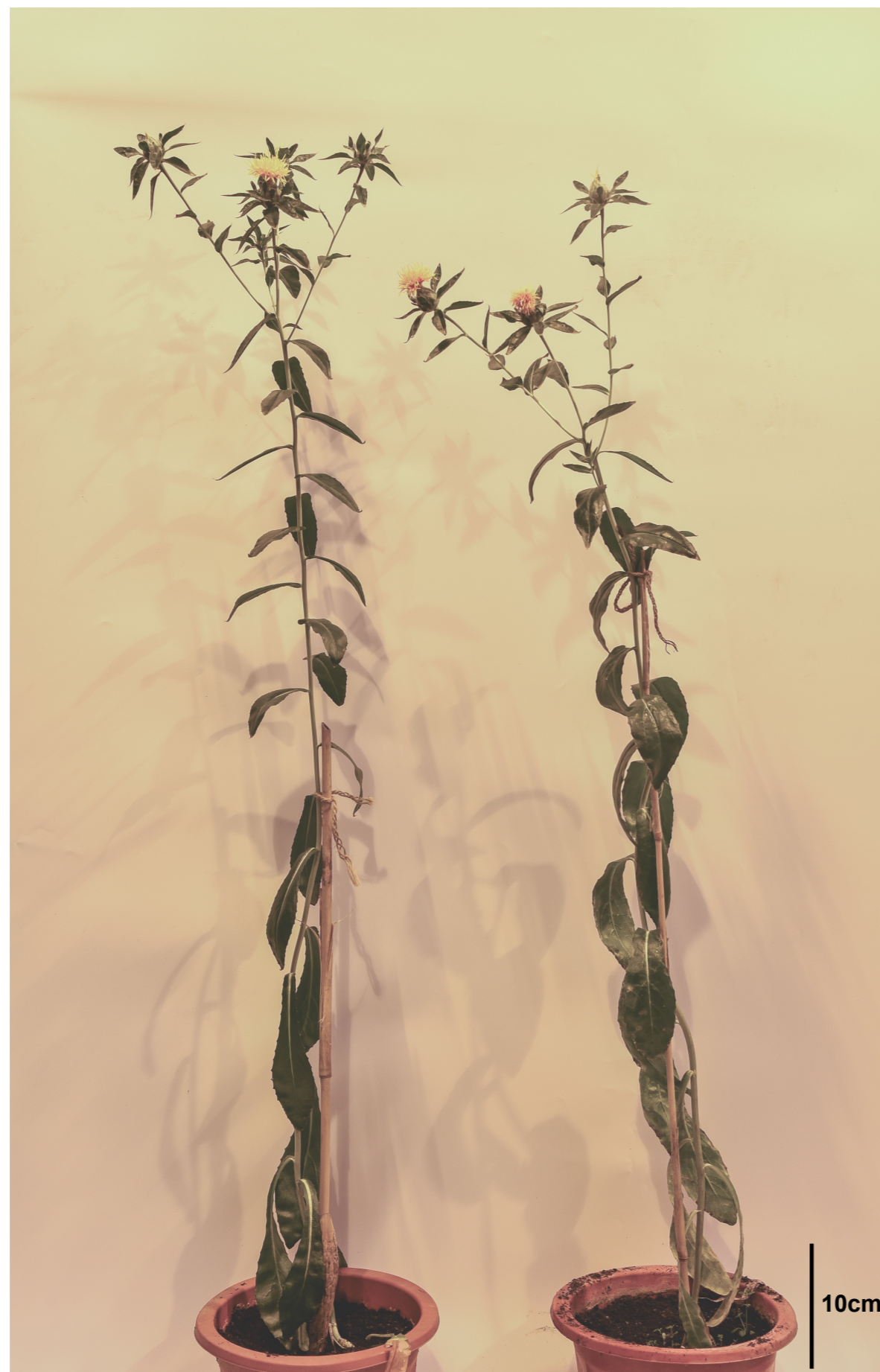

Supplement: Web_Material_uhag068 [file web_material_uhag068.zip › Figure S13. Comparison of wild-type (left) and CtUGT52 overexpressed safflower (right)..pdf]

**a**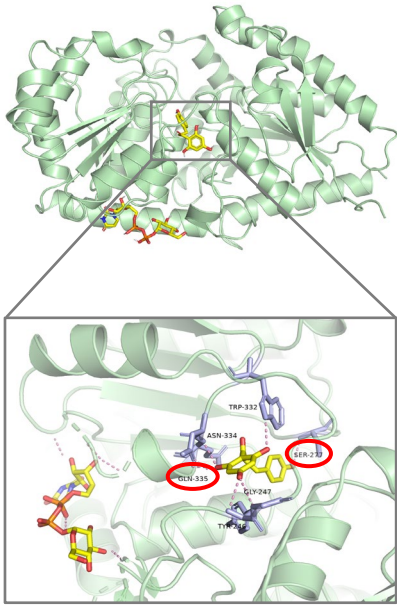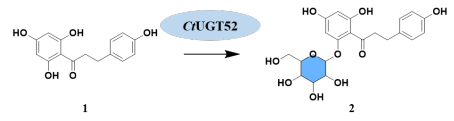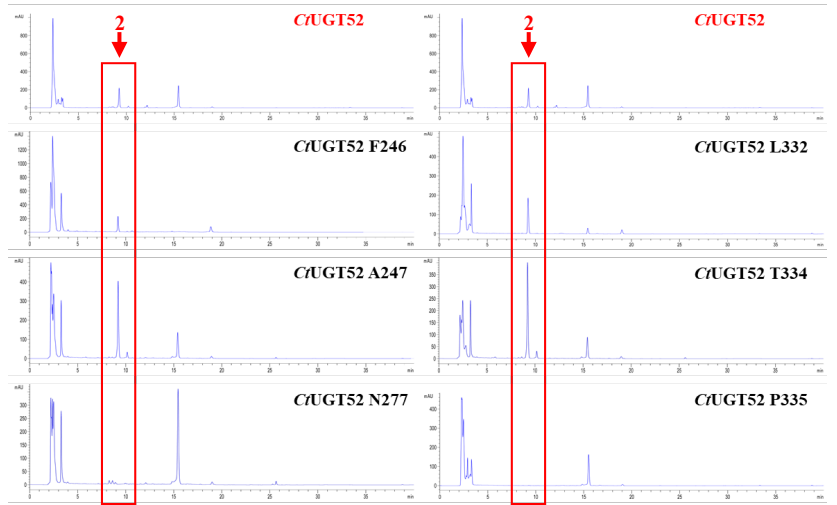**b**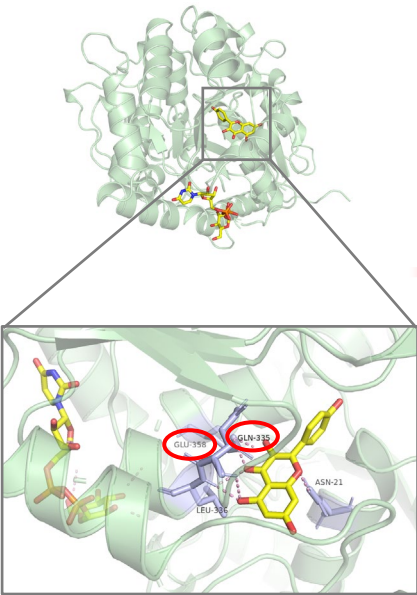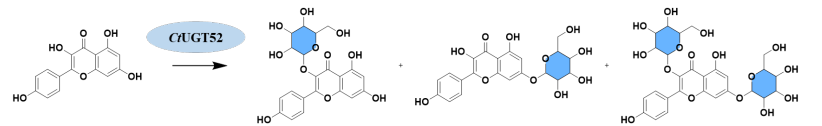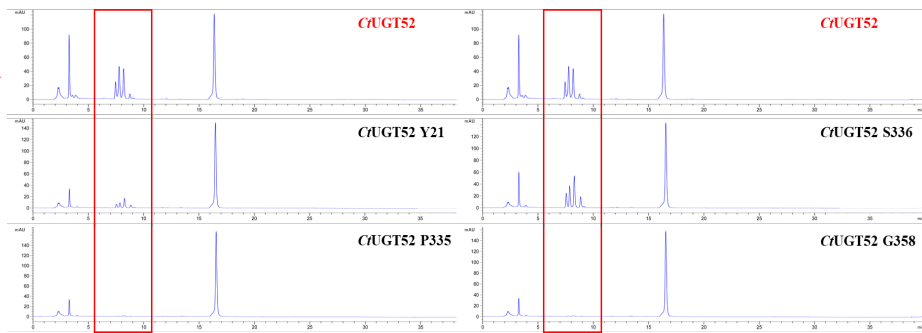**c**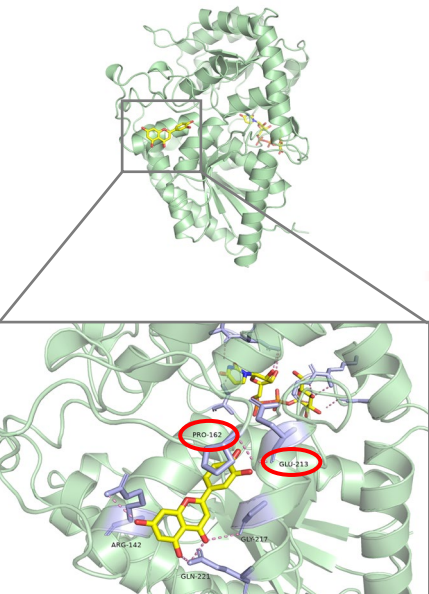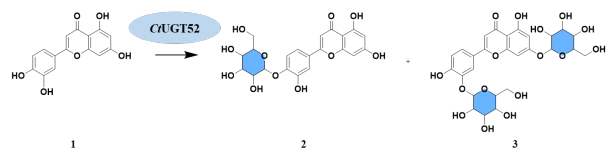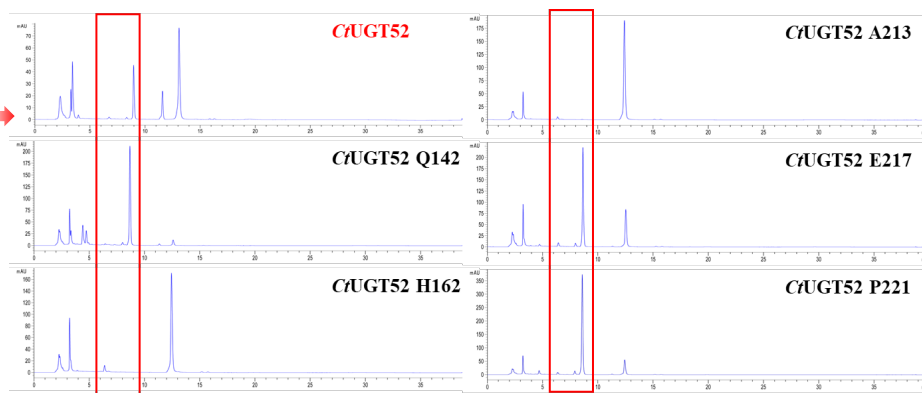

Supplement: Web_Material_uhag068 [file web_material_uhag068.zip › Figure S14. Analysis of the key binding sites of CtUGT52 with Phloretin (a), Kaempferol (b), Luteolin (c) and UDP-Glc.pdf]

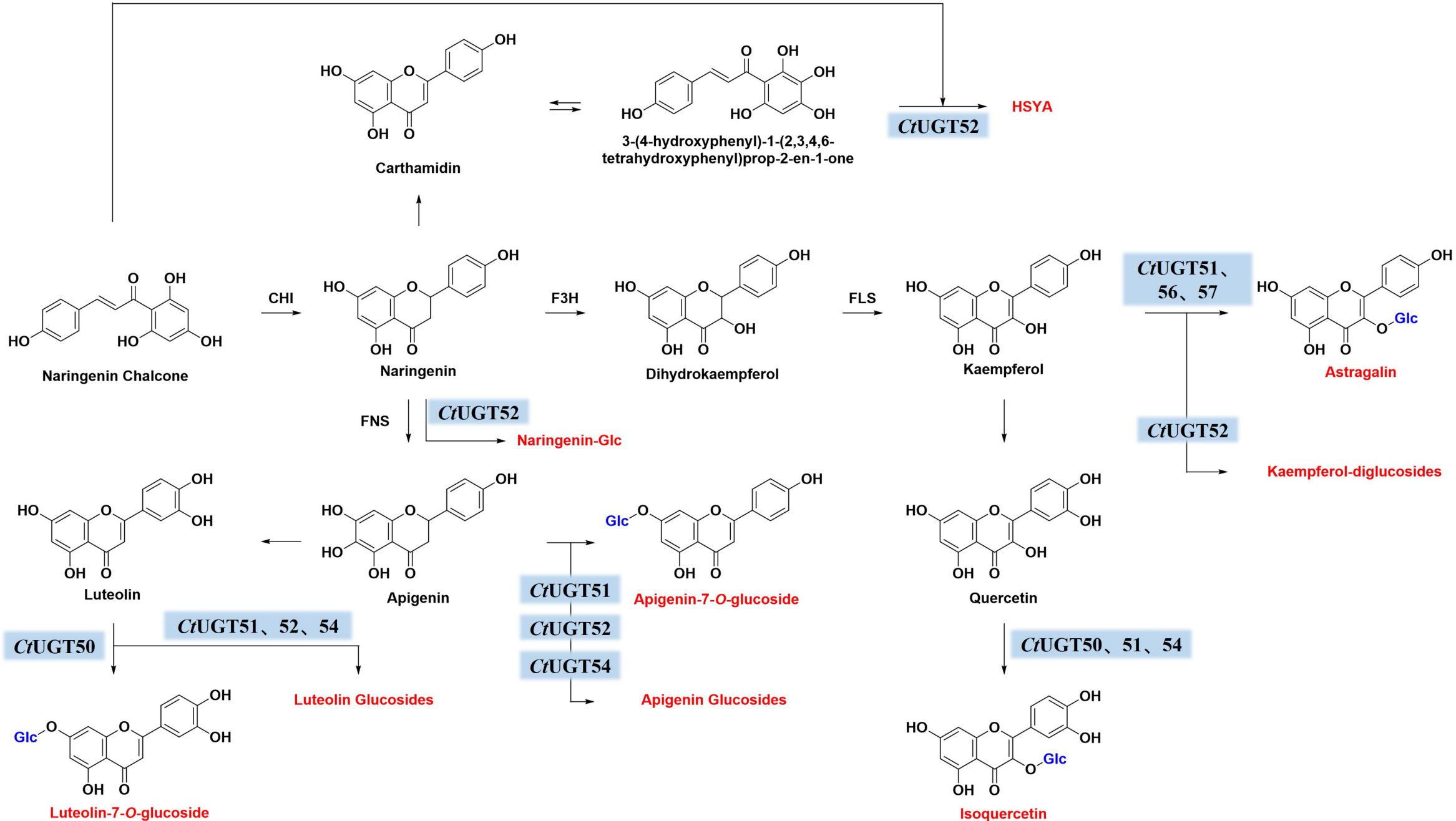

Supplement: Web_Material_uhag068 [file web_material_uhag068.zip › Figure S15. Identified CtUGTs of the different flavonoid glycosides biosynthesis in yellow safflower..pdf]
